# Effect of transcranial pulsed electromagnetic fields (T-PEMF) on functional rate of force development and movement speed in persons with Parkinson’s disease: A randomized clinical trial

**DOI:** 10.1371/journal.pone.0204478

**Published:** 2018-09-25

**Authors:** Anne Sofie Bøgh Malling, Bo Mohr Morberg, Lene Wermuth, Ole Gredal, Per Bech, Bente Rona Jensen

**Affiliations:** 1 Department of Neurology, Odense University Hospital, University of Southern Denmark, Odense, Denmark; 2 Department of Clinical Research, University of Southern Denmark, Odense, Denmark; 3 The Danish Rehabilitation Centre for Neuromuscular Diseases, Taastrup, Denmark; 4 Psychiatric Research Unit, Psychiatric Centre North Zealand, University of Copenhagen, Hillerød, Denmark; University of Toronto, CANADA

## Abstract

**Background:**

Parkinson’s disease is caused by dopaminergic neurodegeneration resulting in motor impairments as slow movement speed and impaired balance and coordination. Pulsed electromagnetic fields are suggested to have neuroprotective effects, and could alleviate symptoms.

**Objective:**

To study 1) effects of 8-week daily transcranial pulsed electromagnetic field treatment on functional rate of force development and movement speed during two motor tasks with different levels of complexity, 2) if treatment effects depend on motor performance at baseline.

**Methods:**

Ninety-seven persons with Parkinson’s disease were randomized to active transcranial pulsed electromagnetic field (squared bipolar 3 ms pulses, 50 Hz) or placebo treatment with homebased treatment 30 min/day for 8 weeks. Functional rate of force development and completion time of a sit-to-stand and a dynamic postural balance task were assessed pre and post intervention. Participants were sub-grouped in high- and low-performers according to their baseline motor performance level. Repeated measure ANOVAs were used.

**Results:**

Active treatment tended to improve rate of force development during chair rise more than placebo (P = 0.064). High-performers receiving active treatment improved rate of force development during chair rise more than high-performers receiving placebo treatment (P = 0.049, active/placebo: 11.9±1.1 to 12.5±1.9 BW/s ≈ 5% / 12.4±1.3 to 12.2±1.3 BW/s, no change). No other between-treatment-group or between-treatment-subgroup differences were found. Data on rate of force development of the dynamic balance task and completion times of both motor tasks improved but did not allow for between-treatment differentiation.

**Conclusion:**

Treatment with transcranial pulsed electromagnetic fields was superior to placebo regarding functional rate of force development during chair rise among high-performers. Active treatment tended to increase functional rate of force development while placebo did not. Our results suggest that mildly affected persons with Parkinson’s disease have a larger potential for neural rehabilitation than more severely affected persons and indicate that early treatment initiation may be beneficial.

## Introduction

Parkinson’s disease (PD) is a neurodegenerative disease primarily affecting the dopaminergic neurons in the basal ganglia resulting in functional motor impairments such as slow movement speed (bradykinesia), rigidity, tremor and impaired balance and coordination of movements. The disease influences the ability to activate the muscles fast and without co-activation of inappropriate muscles [1, 2]. This is reflected in a lower voluntary rate of force development (RFD) in both isometric and functional setups compared to age-matched healthy peers [2–5] in spite of an intact capacity of force generation at the muscular level [4]. RFD is important in performing daily activities where the time available for force generation is short, e.g. managing balance challenging tasks, response to sudden mechanical perturbations, and safe locomotion.

According to the UK Parkinson’s Disease Society Brain Bank Diagnostic Criteria, the criteria for being diagnosed with PD is bradykinesia along with at least one of the following: muscular rigidity, rest tremor (4–6 Hz) and/or postural instability. Although the diagnostic criteria are clear, the progression of the disease and the relative severity of a given symptom are quite heterogenic between patients [6, 7]. This results in heterogeneity of specific motor function. Therefore, investigations of subgroups based on motor performance may be valuable.

In vitro (cell-line studies) and in vivo (animal studies) treatment with pulsed electromagnetic fields (PEMF) has been suggested to have potential neuroprotective effects. For example, PEMF were shown to regulate neutrophic factors such as BDNF, S100 and NGF [8, 9], enhance cell proliferation and differentiation [8, 10], enhance neurite outgrow [11], reduce apoptosis [10], stimulate angiogenesis [12], increase microvascular perfusion and tissue oxygenation [13], and stimulate neurogenesis in the hippocampal dentate gyrus [14] and in the sub ventricular zone after lesion of substantia nigra [15]. The molecular mechanisms initiated by the applied PEMF are not yet fully understood. However, PEMF may affect the tissue directly by the interaction mechanism between the electromagnetic fields and conductive tissue, and indirectly by initiating biological events leading to a physiologic response [16].

In healthy humans, PEMF applied transcranially (T-PEMF) has been suggested to acutely induce enhanced excitatory neurotransmission and/or decreased inhibitory neurotransmission, though resting or active motor threshold was not affected [17]. This could increase the capability to activate muscles fast, since an enhanced excitatory neurotransmission may facilitate a larger neural drive to the muscles during explosive force production, and thus increase RFD.

To our knowledge, the effect of T-PEMF on motor function in PD has not previously been studied, whereas other non-invasive neuromodulation techniques have been shown to improve motor function in persons with PD. For example, repetitive transcranial magnetic stimulation was shown to induce positive effects on motor function that last at least a month [18, 19]. However, transcranial magnetic stimulation usually involves stimulation of the cortical neurons at intensities close to or above the resting motor threshold, and it has to be performed in a clinical setup. On the contrary, the T-PEMF technique uses low intensity stimulation [16] and may be applicable as a homebased treatment.

Based on the literature, we hypothesized that treatment with T-PEMF has the potential to improve RFD and movement speed in persons with PD. Our aims were 1) to study the effect of an 8-week daily homebased T-PEMF treatment on functional RFD and movement speed during two lower extremity motor tasks with different levels of movement complexity, and 2) to study if the treatment effect is dependent on motor performance level at baseline.

## Methods

### Study design

All persons with idiopathic Parkinson’s disease (PDPs) included in the present study were participants in a double-blinded block randomized clinical trial investigating the effect of T-PEMF on PD (clinicaltrials.gov registration# NCT02125032) [20, 21]. PDPs were recruited from Odense University Hospital and private neurologists in Denmark from May 2014 to August 2015. They were randomized to receive 8 weeks of active or placebo treatment. The active and placebo group were of equal size. A third party person conducted the allocation. PDPs were allocated to a number on the allocation sequence list in order of inclusion. Sample size calculation for the trial was based on the primary outcome of the trial, The Unified Parkinson’s Disease Rating Scale (UPDRS) [20], which is a partly subjective clinical measure of disease severity based on observations and patient reports. The mean difference on the UPDRS between active and placebo treatment was estimated to be 3 points from baseline to endpoint with a pooled standard deviation of this mean difference of 7. With a significance level of 0.05 and a power of 0.80, the sample size was estimated to be 90 participants, i.e. 45 in each treatment group. For details, see S2 Text. The trial was approved by The Regional Scientific Ethical Committees for Southern Denmark (S-20130114) and The Danish Health Authority (CIV-14-01-011780), and was conducted in accordance with the Declaration of Helsinki. All participants provided written informed consent prior to participation. No statistical detectable effect of T-PEMF relative to placebo treatment was found for the primary outcome [20]. The present study reports on secondary outcomes.

### Participants

**Fig 1:**
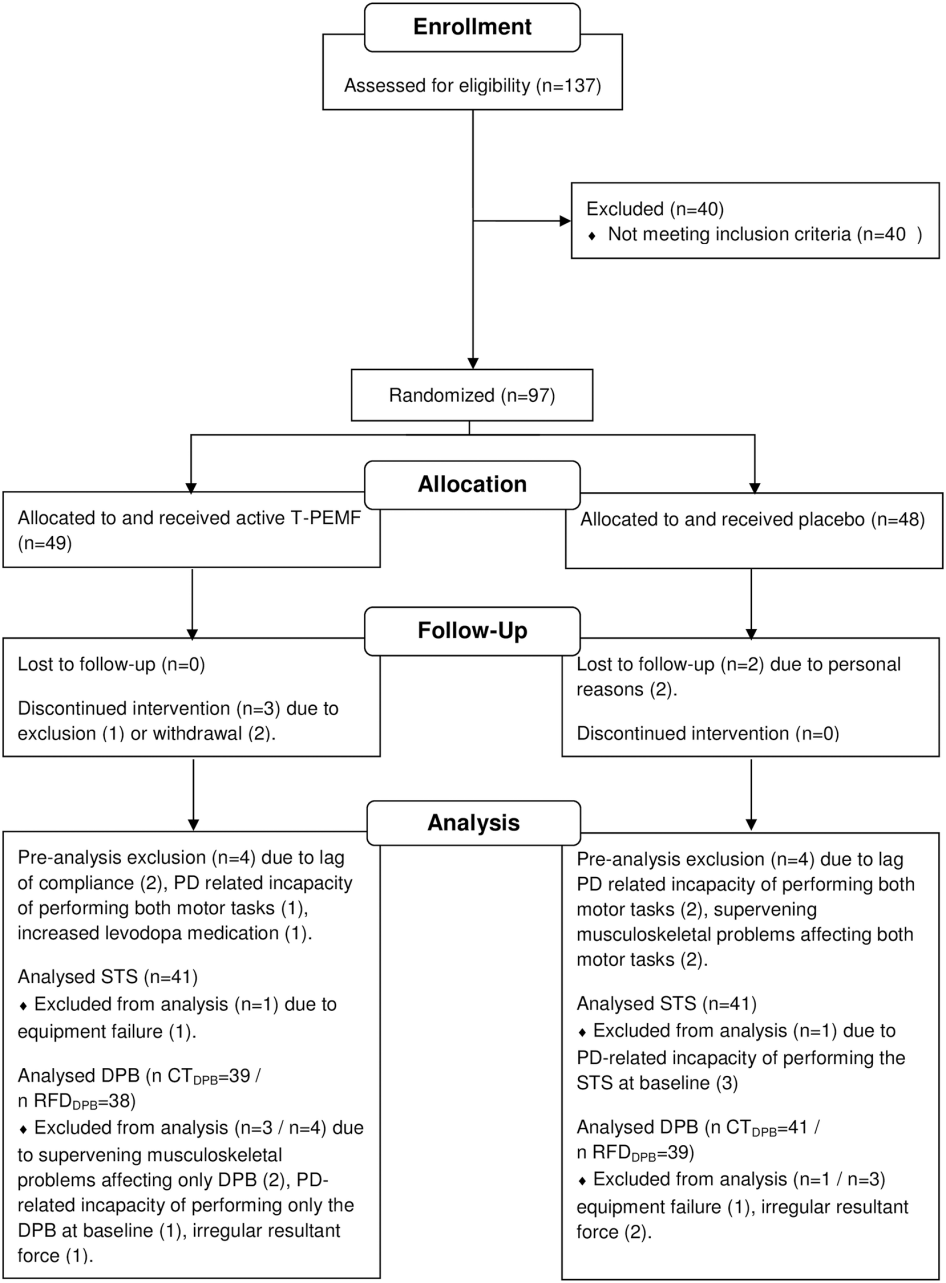
Participant flow diagram. Flow of participants with Parkinson’s disease (PD) during enrollment, allocation to active or placebo treatment with transcranial pulsed electromagnetic fields, follow-up after 8 weeks of treatment, and analysis. Sit-to-stand (STS). Dynamic postural balance (DPB). Completion time of DPB (CT_DPB_). Functional rate of force development during DPB (RFD_DPB_).

Ninety-seven PDPs were included in the clinical trial (Fig 1). Inclusion criteria were a diagnosis of idiopathic PD (Hoehn & Yahr I-IV) according to United Kingdom Brain Bank Criteria; stable and optimal medical treatment regarding PD six weeks prior to and during the intervention period; Mini Mental State Examination >22; age >18 and cognitive skills enabling certification in the use of the T-PEMF device. Exclusion criteria were structural brain damage affecting the ability to give consent; severe psychopathological disturbances; substance abuse; active medical implants; pregnancy or nursing; current or previous cancer in the brain, neck or head region; leukemia; autoimmune disease; epilepsy; and open scalp wounds. In addition, participants with a post interventional treatment compliance of less than 80% were excluded from the analyses.

To describe the severity of the disease and medication level, the Unified Parkinson’s Disease Rating Scale Total score (UPDRS Total) and Motor score (UPDRS Motor) [22] were assessed and daily levodopa equivalent dose (LED) were calculated according to Tomlinson et al. [23].

In addition, 43 healthy participants matched on age and sex distribution were included from Dec 2015 to May 2016 as a reference group. The reference group was tested once and used as a reference frame for subgrouping.

To explore if the baseline performance level of each evaluation parameter had an influence on the effect of T-PEMF, we divided the PDP group into two subgroups according to their baseline performance level of each parameter in each task. Since RFD is influenced by age [24] and all the performance parameters correlated with age (S1 Table), we conducted a linear regression analysis on each parameter as a function of age in the reference group to gain expected values of each parameter at a given age. The standard deviation of the parameter of the whole reference group was used as tolerance. Thus, if a PDP had a baseline RFD value below the expected mean according to age minus 1 SD (higher values of RFD represent better performance) he/she was considered a low-performer (PDP_Low_) of the particular RFD-parameter. Equally, if a PDP had a baseline completion time (CT) above the expected mean according to age plus 1 SD (lower values of CT represent better performance) he/she was considered a PDP_Low_ of the particular CT-parameter. Otherwise, a PDP was considered a high-performer (PDP_High_) of the particular parameter.

### T-PEMF treatment

The PDPs received one daily 30-min session of homebased active T-PEMF or placebo treatment for 8 weeks. The T-PEMF device (Re5 NTS Parkinson Treatment System, Re5, Frederiksberg, Denmark) consisted of a pulse generator and a head applicator with 7 circular coils located as follow: one in the central occipital region, one in the frontal-parietal region (bilateral), and two in the anterior-temporal and posterior-temporal region (bilateral). During active T-PEMF treatment, the pulse generator supplied the coils with squared bipolar pulses of ±50 V at 50 Hz with a pulse duration of 3 ms. The stimulation intensity depends on distance from coil and also the closeness to the coil in horizontal position. At the periphery of the coil and close to the skull maximal stimulation was 2.5 mV/cm and decreased with distance [25]. During placebo treatment, no pulsed electromagnetic fields were generated (sham stimulation). The treatment type (active or placebo) was determined by a chip-card inserted in the pulse generator. A third party person encoded the chip cards. The interface on the pulse generator had the same appearance no matter if the chip card was encoded with an active T-PEMF or a placebo treatment. All use of the T-PEMF device was stored on the chip-card and these records were used to determine treatment compliance. During generation of electromagnetic fields, the T-PEMF device produced a very faint humming sound (6.1 dB, ~50 Hz, below the level of detection according to ISO 266:2003) but no heat or skin sensation [26]. Thus, it was not possible to see or feel difference between active and placebo treatment, and both PDPs and investigators were blinded to the treatment allocation until all PDPs had completed their treatment period and endpoint assessments.

The PDPs were certified in use of the equipment at baseline. They received a home visit during the first week of the treatment period to ensure correct use. Furthermore, they received two telephone calls each week from an investigator to encourage high compliance and register potential adverse events.

The reference group did not receive any treatment.

### Assessment of motor function

The participants were evaluated on RFD and CT of two motor function tasks: a six-cycle sit-to-stand (STS) and a dynamic postural balance (DPB) task assessed in a movement laboratory. Assessments were performed at baseline before the initiation of treatment later the same day, and at endpoint the day after the last treatment session. PDPs followed their usual medication schedule throughout the study and all assessments were performed in self-reported ON-state at baseline and at endpoint.

#### Sit-to-stand (STS)

**Fig 2:**
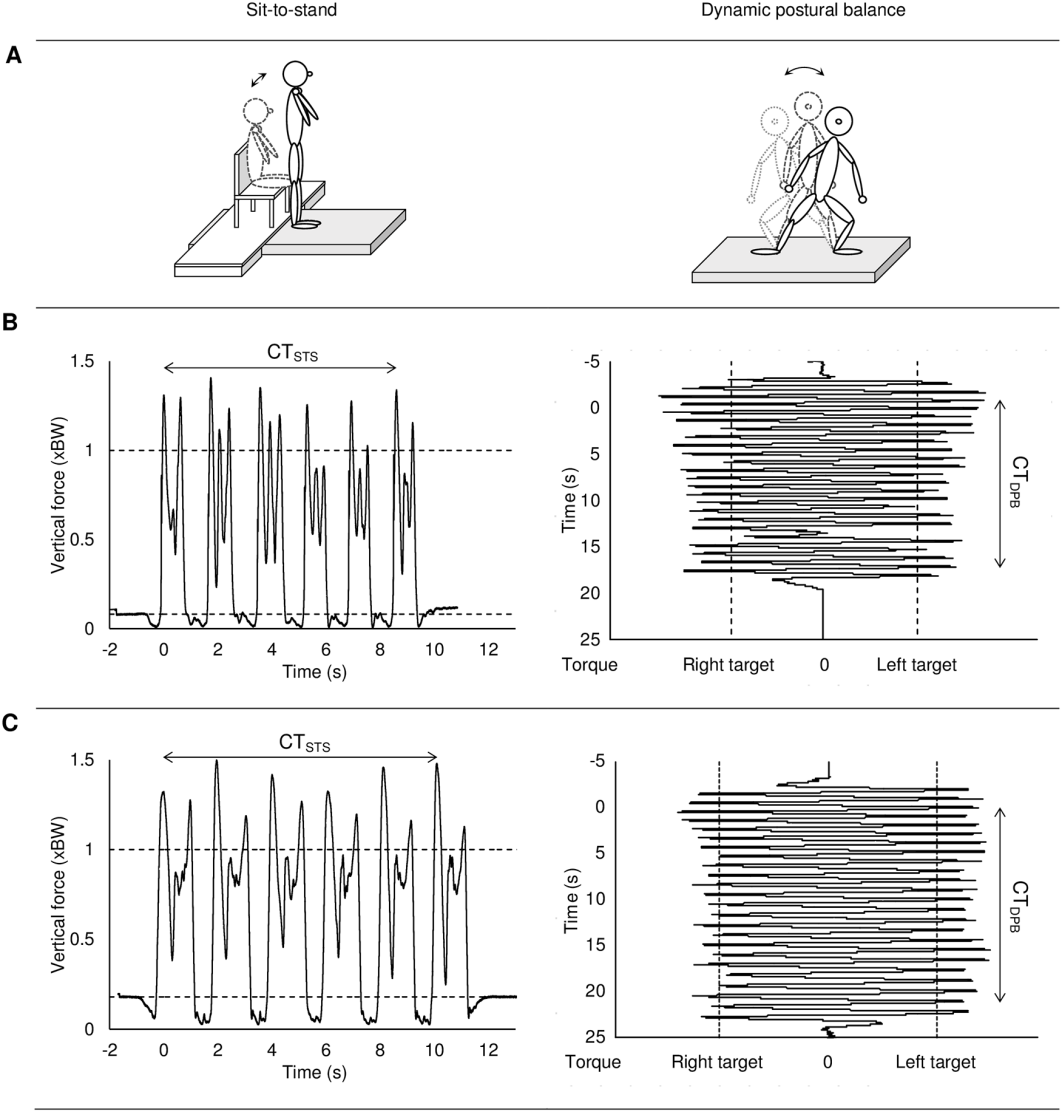
Illustration of the sit-to-stand (left) and dynamic postural balance task (right). **A** Schematic illustration of the tasks. **B** Example of raw data from a representative healthy reference participant. **C** Example of raw data from a representative person with Parkinson’s Disease. Completion time for dynamic postural balance (CT_DPB_). Completion time for sit-to-stand (CT_STS_).

While seated in a custom-built chair allowing only the bare feet to exert force on a force plate (AMTI, USA, 1 kHz sampling), the participant performed trials of six STS cycles as fast as possible [3, 27] (Fig 2A). The seat height was 120% tibia length [28]. The inter-feet distance was the width of the shoulders, the knee angle was 90°-100°, and the arms were placed across the chest. At least three approved trials were performed after task familiarization. A trial was approved if the knees were fully extended while standing, the back touched the backrest while seated, and the feet and hands retained their positions throughout the trial. To ensure maximal performance, an additional trial was performed if the last approved trial was the fastest. The test-retest reliability of the completion time conducted by the described protocol has previously been shown to be high among persons with PD (intraclass correlation coefficient of 0.97 [27]).

#### Dynamic postural balance (DPB)

While standing barefooted on a force plate, 46 side-to-side movements were performed as fast as possible producing sufficient alternating ground reaction torque around the anterior-posterior axis to exceed predetermined target torques without lifting the feet [3, 29] (Fig 2A). Standardized feet positions with 50% leg length (trochanter major to ground, barefooted) between the posterior midpoints of the calcanei and a 10° outward rotation of the feet were used. The target torque was 90% of the torque produced by one-legged still stance in the standardized foot-positions. Visual on-line feedback on produced torque and target torques were provided. Standardized verbal pacing was given. The arms were used as desired by the participant. At least three approved trials were performed after task familiarization. A trial was approved for analysis if at least 35 of the sixth to the 46^th^ torque peaks exceeded 95% of the target torque. To ensure maximal performance, an additional trial was performed if the last approved trial was the fastest. The test-retest reliability of the completion time conducted by the described protocol has previously been found to be high among persons with PD (intraclass correlation coefficient of 0.92 [27]).

### Data analysis

The analyses below were performed for the whole active and placebo groups as well as for the PDP_Low_ and PDP_High_ subgroups of the active and placebo groups.

Raw data from the force plate of the STS test were A/D converted (16-bit, Data Translation Inc., USA) and filtered with a zero phase second order 25 Hz low pass Butterworth filter. The vertical ground reaction force was analyzed according to a previous study [3]. In short, the completion time (CT_STS_) was calculated as the time-period from the 1^st^ vertical ground reaction force peak of the first repetition to the first peak of the 6^th^ repetition (Fig 2B and 2C). Thus, CT_STS_ reflected the time period of five repetitions. The rate of force development (RFD_STS_) was determined as the mean slope of the rising force of the first force peak of each of the 2^nd^ to the 6^th^ repetition in the interval of 30–70% exerted peak force. Results are reported as the mean of the two fastest approved trials in body weight per second (BW/s).

The analyses of the DPB test were performed in line with previously described principles [3]. In short, the completion time (CT_DPB_) was calculated as the time from the 6^th^ to the 46^th^ torque peak (Fig 2B and 2C). The reported CT_DPB_ was calculated as the average of the two fastest approved trials. The rate of force development (RFD_DPB_) was determined as the mean slope of the rising resultant force in the coronal plane in the interval of 30–70% of maximal exerted force for each side-to-side movement. Results are reported as the means of the two fastest approved trials averaged between left and right leg in BW/s.

### Statistics

Group differences between active, placebo and REF at baseline were assessed by a one-way ANOVA for age, height and weight, and by χ^2^ test for sex distribution. Group differences between active and placebo at baseline were assessed by a t-test for UPDRS Total, UPDRS Motor, disease duration and LED.

The four parameters CT_STS_, RFD_STS_, CT_DPB_ and RFD_DPB_ were tested for normal distribution (Anderson-Darling). Since CT_DPB_ did not show a normal distribution, the log10 transformation of these data were used for further analysis. All four parameters had equal variances across group (active and placebo) and time levels (baseline and endpoint) as assessed by the Levene’s test. The interventional effects on CT_STS_, RFD_STS_, CT_DPB_ and RFD_DPB_ were analyzed by repeated measure two-way ANOVAs (2 groups × 2 time points) performed as general linear models to identify the differences between groups (active and placebo) and between time points of testing (baseline and endpoint). The model was: Response parameter = group + subject(group) + time + group×time. Post hoc analysis of significant or a tendency of significant interaction term (group×time) was performed by paired t-test within each group across time. Statistics were performed in Minitab (Minitab Release 13.32). The level of significance was set at P ≤ 0.05, and 0.05<P≤0.10 was presented as a tendency.

## Results

### Participants

Of the 97 participants originally included in the clinical trial, two withdrew, one were excluded during the intervention, and two were lost to follow up (Fig 1). In addition, two subjects were excluded because of lag of compliance, three subjects were incapable of performing both the STS and DPB at baseline because of PD related motor deficits, two had developed supervening musculoskeletal problems at follow-up and could not perform STS or DPB, and one had changed the daily levodopa intake. Thus, 84 participants (42 active, 42 placebo) completed baseline and endpoint testing with a treatment compliance above 80%. Of these, some had minor supervening musculoskeletal problems making them uncomfortable in performing one of the tasks. In total, 82 subjects (41 active, 41 placebo) were included in the STS analysis and 80 subjects (39 active, 41 placebo) were included in the DPB analysis. In addition, three subjects (1 active, 2 placebo) were excluded from the analysis of RFD_DPB_ since the data analysis could not be conducted due to irregularity of the resultant force (Fig 1). Group descriptive variables are presented in Table 1. No differences regarding age, height, body weight, disease duration, LED, UPDRS Total, or UPDRS Motor were found between groups at baseline (Table 1). The baseline UPDRS Total and UPDRS Motor grand average scores for the PDP_Low_ subgroups were 47 and 27, respectively, and 42 and 23, respectively, for the PDP_High_ subgroups. The treatment compliance for all 84 participants included in the analyses of at least one of the motor assessments was 98.2%.

**Table 1:** Group descriptive variables.

	All PDP	Active PDP	Placebo PDP	REF	P
N	84	42	42	43	-
Females/males	37/47	18/24	19/23	20/23	0.943
Age (years)	66±8.3	67±6.5	65±9.6	66±8.1	0.775
Height (cm)	173±8.4	173±9.2	174±7.7	174±9.2	0.861
Weight (kg)	77±15.4	77±15.2	76±15.8	75±11.4	0.792
Disease duration (years)	4.9±4.0	5.4±4.6	4.3±3.6	-	0.242
LED (mg/day)	509±335	533±376	485±292	-	0.512
UPDRS Total	44±12.8	45±13.4	43±12.1	-	0.441
UPDRS Motor	25±8.1	26±8.7	24±7.4	-	0.321

Group descriptive variables at baseline for all persons with Parkinson’s disease (PDP), the active group (Active PDP), the placebo group (Placebo PDP), and the healthy reference group (REF). Disease duration is expressed in whole years from diagnose to inclusion. Daily levodopa equivalent dose (LED). Unified Parkinson’s Disease Rating Scale total score (UPDRS Total) and motor score (UPDRS Motor). Data are presented as the mean ± SD.

The adverse events were benign, mild and transient and the frequency did not differ between treatment groups [21].

### Effect of treatment on motor performance

**Fig 3:**
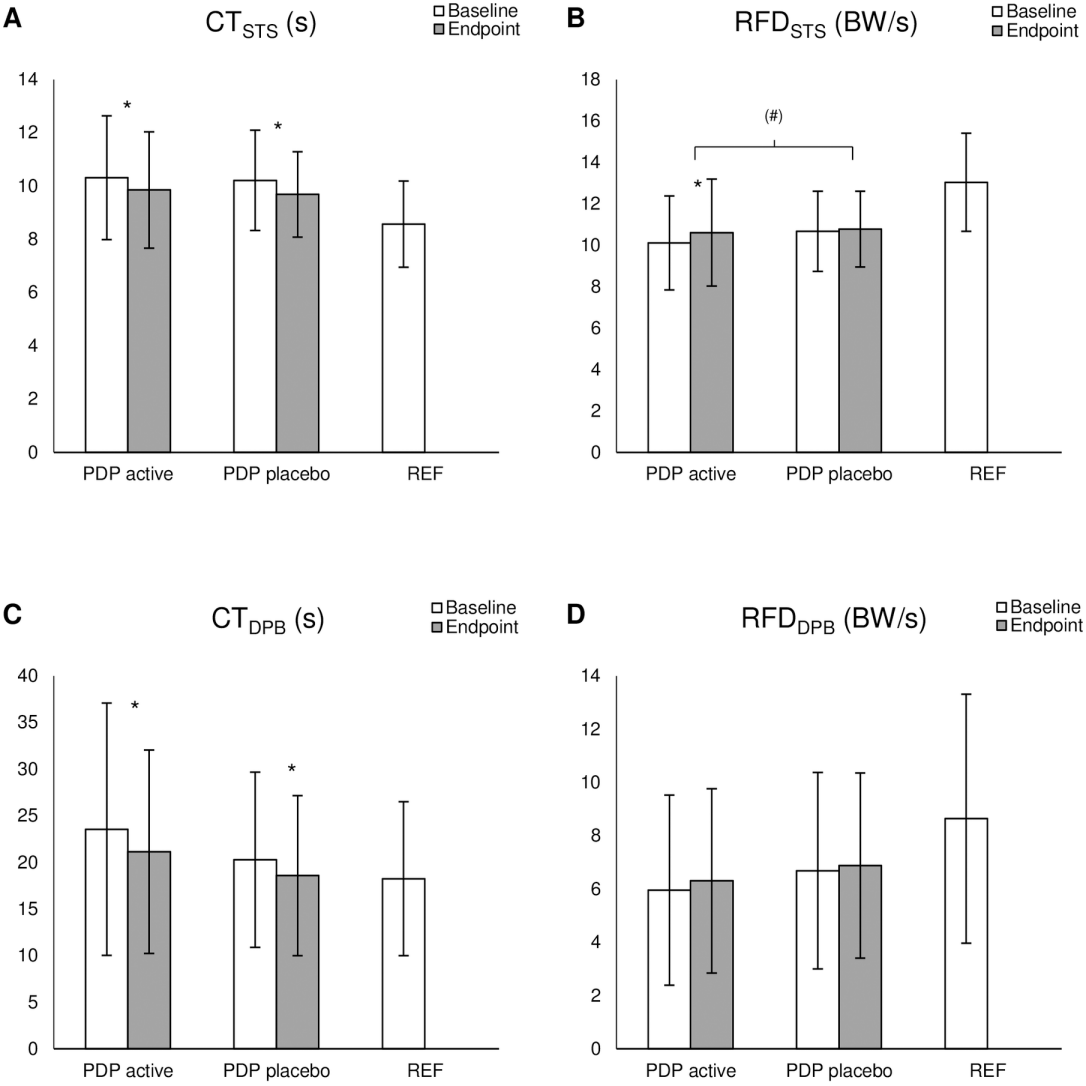
Results for all persons with Parkinson’s disease. **A** Completion time of the sit-to-stand task (CT_STS_), **B** rate of force development of the sit-to-stand task (RFD_STS_), **C** completion time of the dynamic postural balance task (CT_DPB_), and **D** rate of force development of the dynamic postural balance task (RFD_DPB_) for all participants with Parkinson’s disease receiving active (PDP active) or placebo (PDP placebo) transcranial pulsed electromagnetic fields and for healthy reference participants (REF). Bodyweight (BW). Data presented as the mean ± SD. * P≤0.05 for main effect of time. ^(#)^ 0.05 < P < 0.1 for the group×time interaction effect.

Results from the STS showed a tendency of group×time interaction effect on RFD_STS_ (F_1,80_ = 3.53, P = 0.064) and a significant main effect of time (F_1,80_ = 8.32, P = 0.005). Post hoc analysis showed that the active group improved their RFD_STS_ by 5% from 10.1±2.3 BW/s at baseline to 10.6±2.6 BW/s at endpoint (T = -3.17, P = 0.003), whereas the placebo group did not change from baseline to endpoint (baseline: 10.7±1.9 BW/s, endpoint: 10.8±1.8 BW/s, T = -0.76, P = 0.450) (Fig 3B). Further, we found a significant effect of time on CT_STS_ as the two groups combined improved 4.8% from 10.3±2.1 s at baseline to 9.8±1.9 s at endpoint. (F_1,80_ = 17.08, P<0.001). No difference between groups (F_1,80_ = 0.09, P<0.760) or group×time interaction effect (F_1,80_ = 0.08, P<0.773) was found (Fig 3A).

Concerning the DPB, no significant interaction or main effects were found for RFD_DPB_ (Fig 3D). However, the ANOVA showed a significant effect of time in CT_DPB_ (F_1,78 =_ 19.09, P<0.001) across groups but no significant main effects of group (F_1,78 =_ 1.57, P = 0.214) or group×time interaction effects (F_1,78 =_ 0.12, P = 0.734). The mean CT_DPB_ for both groups combined improved 9.4% (baseline: 21.9±11.6 s, endpoint: 19.8±9.8 s) (Fig 3C).

### Effect of T-PEMF on low- and high-performers

**Fig 4:**
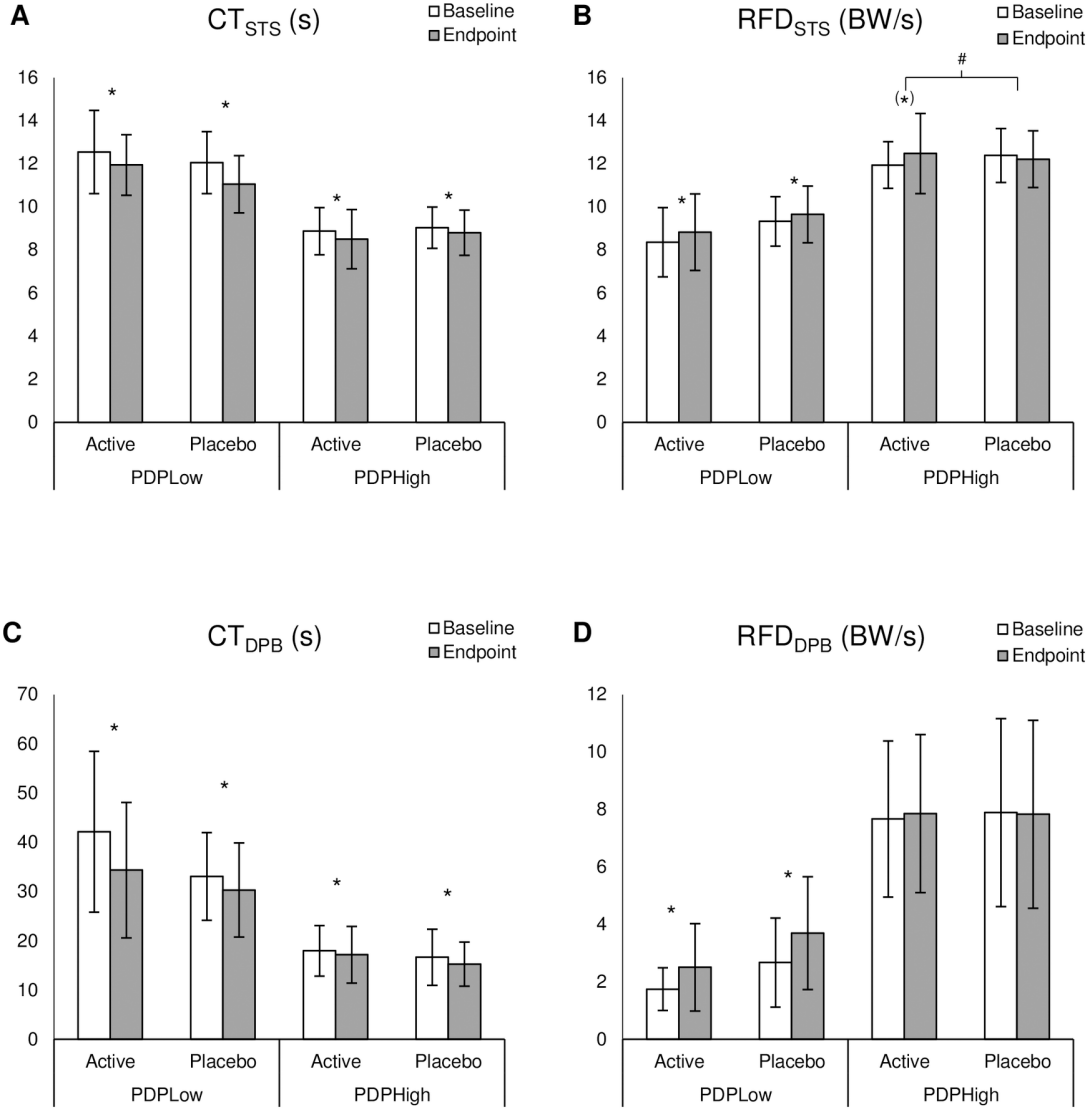
Results for subgroups. **A** Completion time of the sit-to-stand task (CT_STS_), **B** rate of force development of the sit-to-stand task (RFD_STS_), **C** completion time of the dynamic postural balance task (CT_DPB_), and **D** rate of force development of the dynamic postural balance task (RFD_DPB_) for low-performers (PDP_Low_) and high-performers (PDP_High_) of the persons with Parkinson’s disease receiving active or placebo transcranial pulsed electromagnetic fields. Bodyweight (BW). Data presented as the mean ± SD. * P≤0.05 for main effect of time. ^(^*^)^ 0.05 < P < 0.1 for the main effect of time. ^#^ P≤0.05 for the group×time interaction effect.

For the RFD_STS_ we found a significant group×time interaction (F_1,36_ = 4.16, P = 0.049) among PDP_High_. Thus, the active PDP_High_ group increased functional RFD during chair rise superiorly to the placebo group. The active PDP_High_ group had a tendency towards improvement of 5% from 11.9±1.1 BW/s at baseline to 12.5±1.9 BW/s at endpoint (T = -1.95, P = 0.066), whereas the placebo PDP_High_ group did not change (baseline 12.4±1.30.30 BW/s, endpoint 12.2±1.30.31 BW/s, T = 0.86, P = 0.404) (Fig 4B). In contrast, in the PDP_Low_ group we found significant improvement with treatment across the active and placebo groups (F_1,42_ = 10.02, P = 0.003) along with a difference between groups across time (F_1,42_ = 4.45, P = 0.041), but no difference in improvement between groups (Fig 4B).

For the CT_STS_ and CT_DPB_ we found improvements with treatment across groups in both PDP_Low_ (CT_STS_: F_1,30_ = 10.75, P = 0.003; CT_DPB_: F_1,16_ = 10.05, P = 0.006;) and PDP_High_ (CT_STS_: F_1,48_ = 7.07, P = 0.011; CT_DPB_: F_1,60_ = 10.15, P = 0.002) (Fig 4A & 4C). For RFD_DPB_ the improvement with treatment across groups was only found in the PDP_Low_ (F_1,18_ = 9.88, P = 0.006). In addition, we found a tendency of difference between groups across time in the PDP_Low_ (F_1,18_ = 3.09, P = 0.096) (Fig 4D). For PDP_High_ no differences across time or groups were found (Fig 4D).

## Discussion

The main finding of this study indicates a probable positive effect of treatment with T-PEMF on RFD during chair rise, and that this positive effect was present among the participants with high performance levels at baseline. In addition, we found improved motor performance in PDP from baseline to endpoint independently of the treatment received.

Our data showed that the T-PEMF treatment was superior to placebo in the PDP_High_ group for RFD_STS_, whereas this was not the case among the PDP_Low_ group indicating a group specific effect. Muscle activation deficit is common in PD and maximal voluntary activation of the quadriceps muscle during isometric knee extension has been shown to be negatively associated with disease severity in terms of UPDRS Motor score [1]. Furthermore, the UPDRS Motor score has been shown to correlate with the degree of dopaminergic degeneration in PD [30]. Thus, it seems plausible that the PDP_High_ group is less affected by dopaminergic degeneration than the PDP_Low_ group. It is therefore likely that the PDP_High_ group has a higher capacity for neuro repair than the PDP_Low_ group. This emphasizes that early initiation of T-PEMF treatment in PDP is important. However, the present results do not exclude the possibility of a positive effect of T-PEMF in the PDP_Low_ group if the treatment period is longer than 8 weeks. Therefore, a study on the effect of long-term treatment with T-PEMF is warranted.

The rate of force development depends on the corticospinal drive to the muscles along with the muscle morphology. In healthy subjects, studies have shown that early explosive force generation is primarily determined by neural components in terms of agonist activation, whereas the later phase of explosive force generation is primarily determined by muscle mechanical components including muscle strength [31]. Both maximal voluntary contraction force and RFD measured during electric stimulation of the quadriceps femoris muscles are intact in persons with mild motor symptoms of PD. However, these patients have a lower voluntary RFD [4] indicating that mechanical muscle performance is intact, that the muscle can be activated maximal voluntarily if the time frame is sufficient, and that what most likely constitutes the problem in voluntary RFD in PD is insufficient rate of corticospinal drive to the muscles in the early muscle contraction phase. This early phase determined by the rate of corticospinal drive may be of utter importance, since the maximal voluntary contraction level is usually not reached during activities of daily living. For example, around 70% of maximal knee extensor strength is produced during self-paced rising from a chair in healthy older adults [32].

Indeed, the agonist drive was shown to be decreased in PD and to be related to the RFD during explosive isometric knee extension [2]. In addition, functional RFD can be improved in PD, for example, by physical training [3]. Thus, the observed increased functional RFD in the present study may be explained by increased rate of agonist drive to the muscles, driven by increased corticospinal output.

Based on the literature, an increased rate of corticospinal drive could be explained by an increased thalamocortical input to the motor cortex regulated by the basal ganglia or an increased excitability of motor cortex. Increased intercortical facilitation, suggested to reflect enhanced excitatory neurotransmission, has been shown as an acute effect of T-PEMF with an intensity of 1.8 mT (75 Hz, mono pulses of 1.3 ms) in healthy subjects [17] indicating a potential of electromagnetic fields of very low intensity to induce cortical changes. To our knowledge, the present clinical trial has applied daily T-PEMF stimulation for the longest period to date, and the influence of repeated stimulation on cortical excitability in this context has not been previously investigated. High frequent rTMS (> 1 Hz) of motor cortex in healthy participants was shown to increase the cortical excitability [33]. Whether this was also the case for the T-PEMF regime used in the present study is uncertain since it implied electromagnetic fields of very low intensity and did not particularly target the motor cortex. Thus, we hypothesize that the observed increased RFD_STS_ can be explained by an increased thalamocortical input.

Furthermore, increased co-contraction has been reported among persons with PD during both isometric [2] and dynamic assessments [34, 35]. Although an increased rate of corticospinal drive seems to be a likely explanation for the increased functional RFD we cannot exclude that reduced co-contraction of the thigh muscles play a role as well, since we did not perform an electromyography assessment in the present study.

If the corticospinal drive has been increased by T-PEMF, it is peculiar that no significant between-group difference in the RFD_DPB_ was observed. This may, however, reflect the considerable higher complexity of the DPB in terms of the demand for rapid integration and implementation of online visual feedback on timing and coordination of a novel, non-routine task [36]. When evaluating the rate of resulting ground reaction force development, we measure how fast the whole body exerts force to the ground. The more complex a task the more components are influencing the rate of force development besides the force development of the muscles. Further, the more components affecting performance the more within subject variability is expected. Thus, the fact that no change in RFD_DPB_ was found is not necessarily in conflict with the finding of increased RFD_STS_ in PDP_High_.

The 5% increase in RFD_STS_ in PDP_High_ is considered to be clinically relevant as any systematic improvement in functional rate of force development reflect improved motor control and motor ability during daily living, although the improvement may not be perceived by all the participants. The 5% increase may not represent the maximal obtainable effect of T-PEMF as 8 weeks of treatment is a relatively short period available to initiate structural changes in the brain. The Danish Health Authority did not permit a longer treatment period in this first study of homebased T-PEMF treatment in PD. We suggest the use of even longer treatment periods in future studies to determine if the improvement can be further amplified.

For RFD_STS_, CT_STS_, and CT_DPB_, we found a significant improvement from baseline to endpoint when disregarding the treatment groups. These improvements could reflect placebo effects, learning effects, or a mix of both. We aimed at reducing the potential effect of learning by familiarizing the participants to the tests before measuring and by continuing measurements until reaching a plateau of performance. Test-retest reliability of the tests in PDPs are substantial [27]. Considerable placebo effects were also reported in clinical trials of PD when investigating invasive and non-invasive neuromodulation techniques (e.g. see [37] for review). For example, sham rTMS was shown to reduce [^11^C] raclopride binding potentials in the striatum of patients with PD, which indicated a placebo induced increase in dopaminergic neurotransmission [38]. Non-motor placebo effects of T-PEMF performed in other patient populations have also been reported [26, 39]. Therefore, although we cannot determine the cause of improvement across groups, we expect the learning effect to be negligible and consider the improvements on CT_STS_ and CT_DBP_ to be a result of the placebo effect.

### Strengths and limitations

A strength of this clinical trial was the high treatment compliance and the large amount of participants relative to previous studies of brain stimulation. To our knowledge, the present clinical trial applied daily T-PEMF stimulation for the longest time period to date, which is a strength. However, further studies on even longer treatment periods are warranted to enable structural neural changes and thereby to get further insight concerning the neuro-mechanical mechanisms associated with T-PEMF in PD.

Placebo effect among PDPs participating in clinical trials is a well-known phenomenon due to the pathophysiology of the disease. In the present study, we have accounted for this by including a placebo group receiving sham stimulation.

Three participants were not able to conduct the motor tasks because of PD-related motor deficits. These participants were among the most severely affected PDPs.

We did not perform adjustment of the P-value in the sub group analysis as we aimed at reducing the risk of confirming a false null-hypothesis. Thus, the reported effects of T-PEMF on subgroup level should be interpreted as probable effects.

All assessments were performed in ON-state. Thus, we did not determine an eventual effect of T-PEMF treatment on motor function in OFF-state. This should be considered in future studies.

## Conclusion

In this study, which is the first on the effect of long-term T-PEMF treatment on functional rate of force development and movement speed in PD, we found that T-PEMF treatment was superior to placebo treatment to increase functional RFD during chair rise in the PDP_High_ group. Specifically, the functional RFD tended to increase in the PDP_High_ group receiving T-PEMF treatment, whereas no effect was found in the PDP_High_ group receiving placebo treatment. Thus, our results support the idea that mildly affected persons with PD have a larger potential for neural rehabilitation than more severely affected PDP. Our data on functional RFD during the more complex DPB task and completion times of both the STS and DPB task improved but did not allow for differentiation between T-PEMF and placebo treatments. In perspective, long-term treatment with T-PEMF could have a potential as an add-on treatment for PD, and the results of the present study suggest that an early treatment initiation may be beneficial. However, studies with even longer treatment periods and in vivo mechanisms of action are recommended.

## Supporting information

S1 Table Correlation between age and outcome measures. (DOCX)

S1 Data (XLSX)

S1 Text Explanation to S2 Data. (DOCX)

S2 Text Sample size calculation. (DOCX)

S3 Text Study protocol. (PDF)

S1 File CONSORT checklist. (DOC)

## Abbreviations

BW: bodyweight
CT: completion time
DPB: dynamic postural balance
LED: daily levodopa equivalent dose
PD: Parkinson’s disease
PDP_High_: high-performers
PDP_Low_: low-performers
PDPs: Parkinson’s disease participants
PEMF: pulsed electromagnetic fields
REF: healthy reference group
RFD: rate of force development
STS: sit-to-stand
T-PEMF: transcranial pulsed electromagnetic fields
UPDRS: Unified Parkinson’s Disease Rating Scale
