# Supplementary material for: Effect of transcranial pulsed electromagnetic fields (T-PEMF) on functional rate of force development and movement speed in persons with Parkinson’s disease: A randomized clinical trial
Source: PLoS One. 2018 Sep 25;13(9):e0204478. doi: 10.1371/journal.pone.0204478 (PMC6155540; doi:10.1371/journal.pone.0204478)
Supplement: S1 Table — (DOCX) [file pone.0204478.s001.docx]

S1 Table

**Correlation between age and outcome measures.**

Results of Pearson correlations between age and completion time of the sit-t-o-stand task (CT_STS_), functional rate of force development during chair rise (RFD_STS_), completion time of the dynamic postural balance task (CT_DPB_), and functional rate of force development during the dynamic postural balance task (RFD_DPB_) for participants with Parkinson’s disease at baseline.

|  | Correlation coefficient | P-value |
| --- | --- | --- |
| Age * CT_STS_ | 0.389 | < 0.001 |
| Age * RFD_STS_ | -0.385 | < 0.001 |
| Age * CT_DPB_ | 0.279 | 0.012 |
| Age * RFD_DPB_ | -0.263 | 0.021 |
