# Supplementary material for: Effect of transcranial pulsed electromagnetic fields (T-PEMF) on functional rate of force development and movement speed in persons with Parkinson’s disease: A randomized clinical trial
Source: PLoS One. 2018 Sep 25;13(9):e0204478. doi: 10.1371/journal.pone.0204478 (PMC6155540; doi:10.1371/journal.pone.0204478)
Supplement: S1 Text — (DOCX) [file pone.0204478.s003.docx]

S1 Text

**Explanation to S1 Data**

Data is presented in an excel project file.

Data from persons with Parkinson’s disease are located in the sheet named “PDP”.

Data from healthy reference persons are located in the sheet named “REF”.

Each row represents one person.

The columns in sheet “PDP” contains the following data (the labels are identical for the data in the “REF” sheet):

| **Column** | **Label** | **Description** |
| --- | --- | --- |
| A | Allocation | Treatment allocation (a is active, p is placebo, r determines a healthy reference person not receiving any treatment) |
| B | UPDRS Total_0 | Total score of the Unified Parkinson’s Disease Rating Scale at week 0 (baseline) |
| C | UPDRS Motor_0 | Motor score of the Unified Parkinson’s Disease Rating Scale at week 0 (baseline) |
| D | LED_0 | Daily levodopa equivalent dose at week 0 (baseline) |
| E | CTsts_0 (s) | Completion time of the sit-to-stand test in seconds at week 0 (baseline) |
| F | CTsts_8 (s) | Completion time of the sit-to-stand test in seconds at week 8 (endpoint) |
| G | sgCTsts | Subgroup allocation according to completion time of the sit-to-stand test at week 0. |
| H | RFDsts_0 (BW/s) | Functional rate of force development during the sit-to-stand test in bodyweight/seconds at week 0 (baseline) |
| I | RFDsts_8 (BW/s) | Functional rate of force development during the sit-to-stand test in bodyweight/seconds at week 8 (endpoint) |
| J | sgRFDsts | Subgroup allocation according to functional rate of force development during the sit-to-stand test at week 0. |
| K | CTdpb_0 (s) | Completion time of the dynamic postural balance test in seconds at week 0 (baseline) |
| L | CTdpb_8 (s) | Completion time of the dynamic postural balance test in seconds at week 8 (endpoint) |
| M | sgCTdpb | Subgroup allocation according to completion time of the dynamic postural balance test at week 0. |
| N | RFDdpb_0 (BW/s) | Functional rate of force development during the dynamic postural balance test in bodyweight/seconds at week 0 (baseline) |
| O | RFDdpb_8 (BW/s) | Functional rate of force development during the dynamic postural balance test in bodyweight/seconds at week 8 (endpoint) |
| P | sgRFDdpb | Subgroup allocation according to functional rate of force development during the dynamic postural balance test at week 0. |

NB: Age, sex, height, and weight have been omitted as part of the anonymization of data.
