# Supplementary material for: Effect of transcranial pulsed electromagnetic fields (T-PEMF) on functional rate of force development and movement speed in persons with Parkinson’s disease: A randomized clinical trial
Source: PLoS One. 2018 Sep 25;13(9):e0204478. doi: 10.1371/journal.pone.0204478 (PMC6155540; doi:10.1371/journal.pone.0204478)
Supplement: S2 Text — (DOCX) [file pone.0204478.s004.docx]

S2 Text

**Sample size calculation**

We conducted a randomized clinical trial with various outcome measures. We chose the Unified Parkinson’s Disease Rating Scale (UPDRS) total score for sample size calculation and considered it a continuous response variable. We wanted two equal sized treatment groups (active and placebo) and to asses them pre and post treatment. Prior data indicate, that the minimal clinically important change of the UPDRS total score is 3 points (SD of change = 7, corresponding to an effect size of 0.43) [1, 2]. To be able to reject the null hypothesis that a true change in mean of 3 points from baseline to endpoint in the active or placebo group with probability (power) 0.8, we would need a group size of 45 subjects. Thus, a sample size of 90 subjects were estimated to be sufficient (45 subjects receiving active and 45 receiving placebo treatment). The Type I error probability associated with the paired t-test test of this null hypothesis is 0.05.

1. Schrag A, Sampaio C, Counsell N, Poewe W. Minimal clinically important change on the unified Parkinson's disease rating scale. Movement disorders : official journal of the Movement Disorder Society. 2006;21(8):1200-7. Epub 2006/05/05. doi: 10.1002/mds.20914. PubMed PMID: 16673410.

2. Hauser RA, Auinger P. Determination of minimal clinically important change in early and advanced Parkinson's disease. Movement disorders : official journal of the Movement Disorder Society. 2011;26(5):813-8. Epub 2011/03/26. doi: 10.1002/mds.23638. PubMed PMID: 21437987.
