# Supplementary material for: Effect of transcranial pulsed electromagnetic fields (T-PEMF) on functional rate of force development and movement speed in persons with Parkinson’s disease: A randomized clinical trial
Source: PLoS One. 2018 Sep 25;13(9):e0204478. doi: 10.1371/journal.pone.0204478 (PMC6155540; doi:10.1371/journal.pone.0204478)
Supplement: S3 Text — (PDF) [file pone.0204478.s005.pdf]

S3 Text

**Study Protocol**

Below are

- the original Danish study protocol (S-20130114, version of March 12 2014) approved by The Regional Scientific Ethical Committees for Southern Denmark March 24th 2014
- a revised edition of the Danish study protocol (version of Dec 4th 2015) approved by The Regional Scientific Ethical Committees for Southern Denmark Dec 8th 2015 which additionally approve testing of a healthy reference group.
- a translated English version of the revised study protocol (version of Dec 4th 2015)

Only the revised version is translated, as this is the most comprehensive version. Nothing from the original version has been deleted in the revised version – only additions have been made.

# Original Danish study protocol (S-20130114)

Version of March 12 2014

Approved by The Regional Scientific Ethical Committees for Southern Denmark March 24th 2014

Forsøgsprotokol:

**Forsøgets formål:**
Formålet med forsøget er at undersøge, om 8 ugers behandling med transkranielle pulserende elektromagnetiske felter (T-PEMF) kan reducere sværhedsgraden af såvel de motoriske som nonmotoriske symptomer hos patienter med idiopatisk Parkinsons sygdom(IPD).
Endvidere er formålet, at afklare hvorvidt patienter med Idiopatisk Parkinsons sygdom oplever en klinisk relevant og statistisk signifikant effekt af den aktive T-PEMF behandling når de behandles dagligt i 8 uger, i forhold til en gruppe der modtager inaktiv T-PEMF.
I forsøget anvendes 3 forskellige effektmål: Kliniske, neuropsykologiske og motoriske. Disse gennemgås senere i protokollen.

**Hypotese**
Vi forventer, af behandlingen med aktiv T-PEMF er signifikant bedre end placebo T-PEMF i forhold til at nedsætte sværhedsgraden af de motoriske og non-motoriske symptomer, som patienter med Parkinsons sygdom oplever. Vi forventer, at regressionen af symptomerne vil kunne give patienterne en markant bedre livskvalitet. Vi forventer, at kunne få et validt statisk materiale der kan understøtte vores hypotese.

## Baggrund og litteratur

### Parkinsons sygdom
Parkinsons sygdom (PD) er en fremadskridende neurodegenerativ sygdom, hvor der sker et omfattende tab af dopaminproducerende nerveceller i bestemte områder i hjernen, specielt i substantia nigra. Årsagen til idiopatisk PD kendes ikke, men det formodes at den skyldes en kombination af genetisk disposition og udefra kommende faktorer som eksempelvis livsomstændigheder, opvækstvilkår, påvirkning fra pesticider, tungmetaller og andre mulige toksiske stoffer. Der er påvist en familiær ophobning af PD i visse familier, specielt hvis sygdommen debuterer meget tidligt, før 40 års alderen. På nuværende tidspunkt er der beskrevet mere end 10forskellige ændringer i arveanlæggene, som alle kan medføre at sygdommen kommer til udtryk. Der er en livslang risiko på 1 ‰ for at få PD, den stiger i takt med alderen (mere end 3 % i alderen +85) [1]. Gennemsnitsalderen for en nydiagnosticeret Parkinson-patient er 60 år. Patienterne har et gennemsnitligt sygdomsforløb på mere end 15 år, med kun lidt reduceret livslængde i forhold til baggrundsbefolkningen. Parkinson-patienter dør ofte af følgesygdomme
som for eksempellungebetændelse [2]. Da patienterne ofte skal leve mange år med sygdommen, vil en påvirkning af behandlingen og dermed sygdomsforløbet være essentiel for livskvaliteten.
Den nedsatte dopaminproduktion i hjernen er ofte fremskreden, når de typiske symptomer fra det motoriske nervesystem, i form af bradykinesi/akinesi (langsom/manglende bevægelse) ledsaget af muskulær rigiditet, stivhed og hvile tremor, præger sygdomsbilledet. Behandlingen af symptomerne ved PD er symptomatisk med dopaminerge lægemidler.
Sygdomsforløbet ved PD er progressivt, og der sker en aftagende virkning af den medicinske behandling efter års terapi. Patienterne oplever den aftagende effekt af medicinen som svingninger i symptombilledet. For nogle patienter ses en tendens til svingninger mellem dyskinesier (ufrivillige bevægelser) som tegn på overdosering af medicin, og parkinson-symptomer, som tegn på manglende effekt. Den medicinske behandling har store konsekvenser for parkinson-patientens motoriske funktionsniveau, og en forværring af PD manifesterer sig ved reduceret gangfunktion, faldtendens og nedsat finmotorik, alle forhold der påvirker ADL-funktioner.

De relativt få parkinsonpatienter med mere komplicerede motoriske symptomer kan behandles med Deep Brain Stimulation (f.eks. tremor-dominant Parkinsons sygdom eller patienter med svære svingninger i behandlingseffekt). En anden behandlingsmetode for disse patienter er anlæggelse af en mavesonde. Ved denne behandlingsmetode gives den dopaminerge medicin direkte ind i tarmen via en pumpe, således at det motoriske funktionsniveau udjævnes (on-off Parkinson, der veksler mellem ufrivillige bevægelser og Parkinson-symptomer).
Parkinson-patienter vil, sammen med de motoriske symptomer, have non-motoriske symptomer. Disse symptomer kan være meget forskellige fra patient til patient men ligeså invaliderende som de motoriske symptomer. De non-motoriske symptomer omfatter påvirkning af det autonome nervesystem med udvikling af for eksempel ortostatisk hypotension, obstipation, vandladningsgener, impotens samt en mere udbredt påvirkning af hjernens generelle funktion, der medfører træthed, søvnforstyrrelser, depression, hallucinationer og demensudvikling. De nonmotoriske symptomer responderer ikke på samme gunstige måde på den dopaminerge behandling, og derfor kan en række andre farmaka være indiceret, for eksempeltillæg af anti-depressiv medicin og demensmedicin. Denne polyfarmaci giver selvsagt øget risiko for interaktioner og bivirkninger. Selv med adækvat medicinsk behandling kan de non-motoriske symptomer være behandlingsrefraktære.
Parkinson-patienter beretter at de har nedsat helbredsrelateret livskvalitet. Livskvaliteten forringes ved forværring af de motoriske og non-motoriske symptomer. PD kan således have stor indflydelse for patientens og dennes pårørendes livsomstændigheder.

**Anvendelse af pulserende elektromagnetiske felter (PEMF) i lægevidenskaben**
Der har i litteratursøgningen været lagt vægt på at undersøge den fysiologiske virkning af T-PEMF, samt at undersøge i hvilke patologiske tilstande T-PEMF anvendes i klinikken herunder osteoartrose samt begyndende mod depression. Endvidere er der søgt litteratur på studier vedrørende TMS der grundlæggende anvender de samme behandlingsprincipper som T-PEMF. Magnetstimulation anvendes både indenfor psykiatrien og neurologien til diagnostik og behandling. Neurologien har anvendt transkraniel magnetisk stimulation (TMS) til undersøgelse af nerveledningshastighed. Magnetteknologi er også basis for anvendelsen af magnetskanneren MR scanner) til visualisering af indre organer. Ved transkraniel magnet stimulation (TMS) appliceres via en spole hurtige ændringer i et magnetfelt over det ønskede stimulationsområde hvorved større elektriske felter udløses i hjernen. I psykiatrien er TMS ved at vinde indpas i den kliniske hverdag i behandlingen af depressive lidelser [3-4], i neurologien er der påvist dokumenteret effekt af TMS på spasticitet [5-7]. Da apparaturet til TMS ikke er flytbart kan behandlingen kun gives i hospitalsregi og på grund af den korte virkningsvarighed, til f.eks. spasticitet, skal den gives flere gange om ugen for at være effektiv.
Transkranial pulserende elektromagnetisk feltbehandling (benævnt T-PEMF) er på mange måder sammenlignelig med TMS-behandling. Men hvor TMS metoden anvender kraftige pulserende elektriske felter nær en styrke som de der udløser aktionspotentialer i nerver, så bygger den her anvendte T-PEMF teknologi på anvendelsen af elektriske felter som er mange størrelsesordner mindre i styrke og ikke ændrer cellers excitabilitet. T-PEMF udnytter pulser på 50 Hz i længere tid, typisk 30 min, medens TMS metoden til sammenligning anvender få pulser. T-PEMF behandlingen er baseret på en aktivering af hjernebarken ved hjælp af små pulserende elektriske felter af en størrelse og med en frekvens, som er vist at aktivere en række celletyper og enzymatiske processer blandt andet i hjernevæv.

Elektromagnetiske felter (EMF) har vist effekt på endogene neurale stamceller i hjernen, hvor det ser ud som om EMF kan potentiere proliferation og migration af neurale stamceller og ændre reparationen af myelin ved demyelisering [8]. PEMF er endvidere undersøgt ved knogleheling [9] i klinikken for Parkinsons sygdom [10] og multiple sclerose [11] Ved anvendelse af repetitive Transkranial magnetisk stimulation (rTMS) til Parkinsons sygdom undersøgt i dyrestudier synes dette at kunne ændre neurotransmitter frigørelse, synapse effekt og kan inducere neurogenese [12]. Anvendelse af 5 Hz rTMS til Parkinsonpatienter givet over supplementary motor area (SMA) viser effekt og bedring af håndskriften. Denne effekt forklares som ændring af den corticale excitability i det neurale netværk [13]. Til patienter med depression er der i Danmark gennemført et klinisk studie på 50 patienter med behandlingsrefraktær depression, hvor behandlingen med T-PEMF var eklatant i forhold til placebo-behandlingen[14].
En re-analyse af dette T-PEMF arbejde [15] viser, at effekten ikke alene var på de egentlige depressionssymptomer, men også på de symptomer man ser ved den medicinsk terapiresistente patient, nemlig træthed, manglende initiativ, koncentrationsforstyrrelser og søvnforstyrrelser [16]. Disse non-motor symptomer er også dominerende hos parkinsonpatienter og har stor betydning for deres livskvalitet [17].
Der er belæg for at PEMF har en biologisk effekt på forskellige cellulære niveauer. Den biologiske virkningsmekanisme ved T-PEMF er specielt studeret på endothelcellerne i blodhjernebarrieren [18]. Endvidere synes T-PEMF at kunne øge angiogenese [19], neurit vækst [20], øge perifer nerve regeneration [21-23], proliferation af kondrocytter [24] og vasodilatation [25].
På nuværende tidspunkt udfører professor Per Bech yderligere et studie med T-PEMF på depressive patienter, hvor effekten testes på to patientgrupper. En gruppe behandles en gang om dagen og testes overfor en gruppe der behandles to gange om dagen (en såkaldt dosis-respons-undersøgelse).

**T-PEMF til Parkinson patienter**

Præliminære resultater med T-PEMF viser, at en positiv effekt kan forventes ved PD [26]I 2006 gennemførte overlæge Ole Gredal og overlæge Lene Wermuth et åbent pilotstudie på 8 patienter med PD. Formålet med pilotstudiet var at undersøge, om patienter behandlet med T-PEMF kunne opnå en bedre motorisk funktionsevne (målt ved UPDRS-skalaen), en forbedret livskvalitet (målt ved hjælp af et spørgeskema "PDQ-8") samt en reducering af depressionssymptomer (målt ved hjælp af spørgeskemaet "MDI") gennem behandling med T-PEMF.
Resultaterne i pilotstudiet viste at livskvaliteten blev signifikant forbedret (p = 0,009) og ligeledes blev depressionssymptomerne reduceret signifikant (p= 0,011). Endvidere kunne der konstateres en lille forbedring i den samlede score for den motoriske funktion (UPDRS). Resultaterne fra dette åbne pilotstudium, samt en statistisk analyse af data, førte til ønsket om at gennemføre en større klinisk kontrolleret og dobbeltblindet randomiseret undersøgelse på 120 patienter med PD som der nu søges godkendelse til.
Studiet vurderes at være klinisk relevant da behandlingen med T-PEMF til Parkinson-patienter mangler yderligere evidens, og vil såfremt behandlingen har signifikant effekt, have stor indflydelse på fremtidens behandling af Parkinson patienter.

**Forsøgets metode**

Undersøgelsen med T-PEMF udføres som dobbeltblindt placebokontrolleret forsøg, hvor halvdelen af parkinson-patienterne randomiseres til aktiv T-PEMF behandling via en randomiseringsproces hvor randomization.com anvendes. Randomiseringsprocessen foretages af en person der ellers ikke

er involveret i forsøget. Randomiseringen stratificeres ud fra randomiseringskriterierne: alder, køn, parkinsonstadiet (Hoehn and Yahr Scale) for at minimere bias i studiet. De aktive og inaktive TPEMF apparater vil fremtræde identiske, og under behandlingen vil en kontrollampe blinke. Placebobehandlingen foregår uden dannelse af et elektrisk felt. Deltagerne vil ikke under selve behandlingen kunne mærke eller høre om de modtager aktiv eller inaktiv behandling. Der vil i hvert apparat være et hukommelseskort der aflæser al aktivitet vedrørende anvendelse af apparatet, dette aflæses i et computerprogram hvorved det kan kontrolleres hvorvidt der er compliance i forhold til behandlingen.
Undersøgerne og patienterne er uvidende om hvilken behandling der gives. Patienterne må ikke omtale nogle forhold vedrørende apparatet eller behandlingen for undersøgerne. Overtrædelse af dette medfører eksklusion fra forsøget. Alle procedurer inklusiv information og instruktion i brug af apparaterne udføres af sundhedsfagliguddannet personale.
Statiske beregninger udført ud fra det ikke blindede pilotstudiets resultater, viste at 120 parkinsonpatienter skal inkluderes i undersøgelsen, for at det kan forventes at vise en signifikant effekt på effektmålet livskvalitet (målt med PDQ-8). De 120 patienter fordeles ligeligt henholdsvis i en interventionsgruppe og en placebogruppe. For at opnå en virkning på de enzymatiske processer i hjernen, og dermed en klinisk effekt for patienten af behandlingen, forventes en behandlingsvarighed på mindst 4-5 uger med 1 daglig behandling, hvorfor 8 uger er valgt for at være sikker på at der opnåsen maksimal effekt.
For at studiet skal kunne gennemføres praktiskunder hensynstagen til eventuel variation af specielt motoriske symptomer i løbet af dagen, vil grupper af 6 patienter blive randomiseret og indgå i undersøgelsen med ca. 6 ugers mellemrum. Der er for nuværende 14 apparater til rådighed således at der vil være 2 apparater i reserve.
Patienterne skal søges at være optimalt medicinsk behandlet for deres Parkinsons sygdom før de indgår i studiet. Medicinen tilstræbes at blive holdt uændret under undersøgelsen. Skulle der vise sig tegn til overdosering i løbet af studiet grundet en gunstig virkning af T-PEMF reduceres dosis.
I forsøget anvendes 3 forskellige effektmål: Kliniske, neuropsykologiske og motoriske.
Behandlingseffekten ønskes evalueret ved at registrere og analysere de ændringer der sker på det motoriske og non-motoriske område fra baseline til undersøgelsens afslutning, samt efter en followup periode. I projektet anvendes 3 forskellige effektmål: Kliniske, neuropsykologiske og motoriske.
Til måling af parkinsonpatienternes kliniske symptomer vil ”Unified Parkinson’s Disease Rating Scale” (UPDRS) blive anvendt. Udførelsen af ”Parkinson’s Disease Questionnary 39” (PDQ-39) blive benyttet til kategorisering af parkinsonpatienternes helbredsrelaterede livskvalitet.
Neuropsykologiske effektmål: Depressionstendens vil blive vurderet ud fra Major (ICD-10) Depression Spørgeskemaet, HAM-D6, Apati skalaen og WHO-5. Kognitionen vurderes via MoCA og MMSE. Til overordnet måling af non-motor symptomer anvendes NMS. Til måling af kognitive og neurologiske processer anvendes Stroop. Prøven bygger på antagelsen om, at mennesker læser ord hurtigere end de kan identificere farvenavne. De kognitive funktioner, der måles i testen kan anvendes til at give information om deltagerens evne til at håndtere kognitivt stress og komplekse kognitive stimuli.
Motoriske funktionsmål: Hvile tremor, gangkapacitet (f.eks. 6 Minute walk test), modificeret Timed Up and Go, dynamisk og statisk balance.
T-PEMF apparatet kan ligeledes dagligt opsamle subjektive patientoplysninger i forbindelse med de

to daglige behandlinger, og disse registreringer kan sammenholdes med data fra besøgene på klinikkerne. Dette for at vurdere om behandlingen rent faktisk har været anvendt. Ligeledes registreres patienternes antal skridt pr. dag med skridttællere.
Alle forsøgsdata registreres i patientmapper med angivelse af forløb, eventuelle bivirkninger og komplikationer. Umiddelbart efter foretages indtastning i Databasen under Dansk Selskab for Bevægeforstyrrelser (DANMODIS) eller anden database. I forbindelse med projektet udtages en 5 ml veneblodprøve før og efter behandlingsinterventionen. Denne analyseres for BDNF som markør for nervevækst og for VEGF som markør for angiogenese. Blodprøver analyseres løbende og der oprettes ikke en biobank. Blodprøven tages to gange i studiet af den forsøgsansvarlige og analyseres straks og destrueres øjeblikkeligt herefter.
De motoriske funktionsmål testes på Institut for idræt og ernæring Integreret fysiologi Københavns Universitet. Blodprøvetagning foretages ligeledes på instituttet.
På de to centre beliggende på OUH og i Taastrup foretages måling af de kliniske og de neuropsykologiske effektmål.

**Statistiske overvejelser**

Resultaterne fra forsøget bearbejdes statistisk, interventionsgruppen og placebogruppens resultater sammenlignes. Statistikerfunktionen ved institut for regional Sundhedsforskning vil bidrage med vejledning. De statistiske beregninger vil fastslå validiteten af forsøgsresultaterne.
Statistiske beregninger udført ud fra det ikke blindede pilotstudiets resultater, viste at 120 parkinson -patienter skal inkluderes i undersøgelsen, for at det kan forventes at vise en signifikant effekt på effektmålet livskvalitet (målt med PDQ-8). De 120 patienter fordeles ligeligt henholdsvis i en interventionsgruppe og en placebogruppe. For at opnå en virkning på de enzymatiske processer i hjernen, og dermed en klinisk effekt for patienten af behandlingen, forventes en behandlingsvarighed på mindst 4-5 uger med 1 daglig behandling, hvorfor 8 uger er valgt for at være sikker på at der opnås en maksimal effekt. Det estimeres at der skal være 60 projektdeltagere pr. center. I forhold de præliminære resultater fra effekten af T-PEMF på depression kan et deltager antal interventionsgruppen være 40-50 og stadigvæk vil kunne producere et validt statistisk datasæt. Der er lavet styrkeberegning for primære effektmål UPDRS i dette kommende studie der viser at ved en styrke på 90% og et signifikans niveau på 5% vil 84 deltagere være statistisk signifikant. Det vil sige 42 deltagere i placebogruppen og interventionsgruppen. Derfor vurderes det at en interventionsgruppe på 60 er sufficient for at sikre validiteten af data og samtidigt tage højde for dropouts der estimeres til 10%.
Det skønnes ikke nødvendigt at foretage interim analyse, da der ud fra tidligere studier med behandlingsvarighed på 8 uger ikke er observeret bivirkninger. Da behandlingstiden kun er 8 uger skønnes det ikke etisk uforsvarligt at undlade at udføre interimanalyse.
Designet af studiet er et randomiseret klinisk studie med ét kontinuerligt primært effektmål. Data vil blive anonymiseret og analyseret af en statistiker, som vil være blindet for hvilken behandling patienterne har modtaget dvs. aktiv eller placebo. Deskriptiv statistik for de to grupper vil blive genereret.
Ved intention to treat analyser anvendes LOCT og mixed model. Denne model sikrer at missing data ikke vil skabe bias, hvis missing data er tilfældigt fordelt. Således at der når studiet er afsluttet kan afgøres om ”missing data” er tilfældigt fordelt.
Da Re5 behandling er en ny indfaldsvinkel til behandling af Parkinsons sygdom vil det være værdifuldt at evaluere en mulig effekt af Re5behandling, hvis behandlingen tages som specificeret dvs. compliance ≥80%. Derfor vil per protokol analyser også blive udført.

For at reducere forekomsten af missing data vil primær investigator eller en kvalificeret stedfortræder sikre at alle spørgeskemaer udfyldes på de planlagte tidspunkter. Hvis projektdeltagerne ikke møder op til de aftalte testtidspunkter vil de blive kontaktet telefonisk af primær investigator eller en kvalificeret stedfortræder.
Data vil blive analyseret med SPSS og SAS. Succeskriterierne for afprøvningen er en signifikant forskel til den positive side hos den aktive gruppe sammenlignet med placebogruppen vedrørende primært og sekundære effektmål.

**Inklusion-og eksklusion**
*Inklusionskriterier til forsøget er:*

- Patienter med Idiopatisk Parkinsons sygdom H & Y 1-3 defineret efter standardkriterier.
- MMSE > 22 (anvendes til at screene for begyndende demensudvikling).
- Alder > 18 år.
- Patienten er i stand til at forstå, acceptere og gennemføre de planlagte procedurer.

*Eksklusionskriterier til forsøget er:*

- Patienten har på inklusionstidspunktet tidligere været i T-PEMF behandling.
- Ændringer i den farmakologiske anti-parkinson behandling indenfor de sidste 6 uger.
- Udtalt demens eller anden hjerneskade, der kan influere på evnen til at afgive informeret samtykke, eller vanskeliggøre vurderingen af patientens tilstand.
- Psykotisk lidelse, eller andre psykopatologiske tilstande, der nødvendiggør anden intervention.
- Misbrug af alkohol eller stoffer.
- I behandling med Deep Brain stimulation.
- Graviditet eller amning.
- Epilepsi.
- Aktive implantater i form af pacemakere og andre som f.eks. cochlear implantater.
- Aktivt medicinsk udstyr f.eks. insulinpumper, baklofenpumper og andet.
- Deltagelse i andre forsøg eller afprøvninger i projektperioden.
- Aktuel eller tidligere kræftsygdom i hjernen, leukæmi, modermærkekræft, pladecellekræft, eller andre kræftformer i hoved/hals region.
- Autoimmun sygdom.
- Åbne sår i hovedbund.

Der stilles ingen krav til patienternes køn og alder så længe at de opfylder inklusionskriterier.

**Bivirkninger, risici og ulemper**
Ved transkraniel magnetisk stimulation (TMS) givet i højere doser end dem, der anvendes i dette studie, har der ikke været observeret langtidsbivirkninger.
Behandling med elektromagnetiske felter herunder T-PEMF er stort set uden gener eller bivirkninger [19]. Dette bekræftes af tidligere undersøgelser der ikke har vist bivirkninger vurderet ved bivirkningsskalaen UKU (udvalg for kliniske undersøgelser). I de undersøgelser har antallet af behandlinger været mange og behandlingen har strakt sig over lang tid. Foreløbige rapporter om bivirkningsprofilen for T-PEMF behandling indikerer at behandlingerne kan give let og forbigående kvalme og hovedpine. Endvidere er der i nogle tilfælde rapporteret om ubehag i nakkemusklerne, grundet hjelmens vægt. Der er for nuværende ikke rapporteret om andre bivirkninger.
Da forsøgsdeltagerne kan modtage behandlingen derhjemme, og kan varetage rolige dagligdags funktioner, som for eksempel læsning og strikning, mens de modtager behandlingen og behandlingen har en varighed på 30 min1 gang om dagen, skønnes det ikke at behandlingen bliver til gene for patienten. Det er også vigtigt at understrege, at deltagelse i forsøget ikke har indflydelse på de rettigheder patienten har til behandling for sin Parkinsons sygdom.
Patienterne skal komme til kontrol og testning to gange på institut for idræt og ernæring, integreret fysiologi Københavns Universitet for alle patienter og tre gange på testcentrene henholdsvis i Taastrup og Odense (OUH) alt afhængigt af geografisk inklusionsområde. Det skønnes ikke til væsentlig gene for forsøgsdeltagerne. Transportomkostninger vil blive dækket også for én pårørende. Det skønnes ikke at en veneblodprøvetagning 2 gange i løbet af forsøget vil være til gene for forsøgsdeltagerne da indgrebet er lille og forsøgsdeltageren kun tappes for 10 ml blod samlet under hele forsøget, samt at den forsøgsansvarlige har rutine i blodprøvetagning.
I undersøgelsens forløb registreres ved alle kontakter eventuelle hændelser med en angivelse af, om disse er alvorlige eller mindre alvorlige, samt en angivelse af om de ifølge undersøgerens skøn er relateret til T-PEMF behandlingen. Oplysningerne indsamles ved hjælp af UKU. I tilfælde af alvorlige uønskede hændelser, hvor T-PEMF behandlingen er medvirkende årsag til hændelsen, vil den lokale Videnskabsetiske komité, Sundhedsstyrelsen samt styregruppen omgående blive underrettet.
Endvidere vil Sundhedsstyrelse, den lokale Videnskabsetiske komité og styregruppen ligeledes blive orienteret om alle alvorlige hændelser og næsten-hændelser, som er indtruffet i perioden som helhed, samt give oplysning om forsøgspersonernes sikkerhed. Ved alle hændelser forstås både de forventede og uventede hændelser.

Følgende definitioner gælder for medicinsk udstyr:

En hændelse, der opfylder følgende tre kriterier er indberetningspligtig til Sundhedsstyrelsen:

**Adverse Event (AE):** enhver uønsket hændelse hos en forsøgsperson i en klinisk afprøvning ved/efter brug af et medicinsk udstyr, uden at der nødvendigvis er sammenhæng mellem denne brug og den uønskede hændelse

**Adverse Device Effect (ADE):** en uønsket hændelse relateret til brugen af det medicinske udstyr

**Serious Adverse Event (SAE/næsten hændelse):** en hændelse der medfører
- død
- livstruende skade eller sygdom
- varig skade på kroppen eller kropsfunktioner
- hospitalsindlæggelse eller forlængelse af hospitalsophold
- at medicinsk eller kirurgisk behandling er nødvendig for at undgå ovenstående
- fosterdød, en medfødt anomali eller misdannelse hos fosteret eller anden negativ påvirkning af fosteret

En næsten-hændelse er en udstyrsrelateret hændelse, som ikke har en alvorlig udgang, fordi der er grebet ind, inden hændelsen har udviklet sig.

- **Herunder er også:** alvorlige hændelser og næsten-hændelser der skyldes unøjagtige eller mangelfulde resultater fra diagnostisk udstyr for eksempel:
- fejldiagnose
- forsinket diagnose
- forsinket behandling
- forkert behandling
- og hvor fejlen skyldes tekniske fejl eller mangler ved udstyret, brugsanvisningen, mærkningen, brugen eller vedligeholdelsen af udstyret

**Serious Adverse Device effect (SADE):** en alvorlig hændelse, som er relateret til brugen af det medicinske udstyr.

Studiet vil blive udført i henhold til ISO 14155 og monitoreret af GCP enheden ved Odense Universitetshospital.

**Respekten for forsøgspersonernes fysiske og mentale integritet samt privatlivets fred.**
Oplysninger om forsøgsdeltagerne beskyttes efter lov om behandling af personoplysninger samt sundhedsloven. Projektet anmeldes til datatilsynet og persondataloven overholdes i forsøget.
Den forsøgsansvarlige ønsker at anvendeoplysninger fra patientjournaler på de forsøgsdeltagere der deltager i forsøget. Det drejer sig om oplysninger vedrørende farmakologisk behandling, sygdomsstadie, almene helbredsoplysninger og speciale specifikke oplysninger her tænkes på Parkinsons sygdom. Oplysninger anvendes til at inkludere patienter i forsøget, samt for at kunne udarbejde statistisk valide data ved endpoint. Der vil kun blive læst de dele af journalen der omfatter ovenstående. Det skønnes at disse oplysninger er relevante og nødvendige for forskningsprojektet. Forsøgsdeltagere vil blive grundigt informeret om omfanget af journal gennemgangen og grundlaget for denne.

**Økonomiske forhold.**
Den forsøgsansvarlige og styregruppen modtager ingen vederlag for gennemførelse af forsøget. Hverken den forsøgsansvarlige eller styregruppen har økonomisk interesse i det firma der leverer apparaterne. Endvidere har den forsøgsansvarlige og styregruppen ingen tilknytning til støttegiveren

DEN A.P. MØLLERSKE STØTTEFOND.

Initiativet til forskningsprojektet er taget af en styregruppe bestående af:

Overlæge, klinisk lektor Lene Wermuth (Ph.d.-hovedvejleder)
Overlæge, dr. med Ole Gredal
Lektor, ph.d. Bente Rona Jensen (Ph.d.-vejleder)
Professor, dr.med. Per Bech (Ph.d.-vejleder)
Læge Bo Mohr Morberg (forsøgsansvarlig)

Der foreligger økonomisk bevilling til såvel gennemførelse af projektet samt Ph.d. forløb fra DEN A. P. MØLLERSKE STØTTEFOND med et beløb på kr. 4.962.000 se budget bilag 13.

Det kliniske studie ledes af Neurologisk forskningsenhed OUH. Bevillingen administreres af OUH og er under statsrevision.

Støtten udbetales som en fast sum og der oprettes to forskningskonti i OUH regi der vil være økonomisk administrator. Én konto der dækker lønomkostninger til den forsøgsansvarlige og én konto der dækker omkostninger til øvrig drift.

Et eventuelt overskydende støttebeløb vil blive tilbageført til fonden.

**Relevante klausuler i kontrakten mellem sponsor og forsøgsstedet**
Der er ingen klausuler mellem den A.P. Møllerske Støttefond og den forskningsvarlige og styregruppen vedrørende publicering. Den forsøgsansvarlige har fuld adgang til data og de fulde rettigheder til offentliggørelse af data, producenten af apparatet har ingen adgang til forsøgsdata.

**Vederlag eller andre ydelser til forsøgspersoner.**
Forsøgspersonerne modtager transportgodtgørelse efter gældende takst vedrørende kørselsgodtgørelse ved kørsel i egen bil eller billigste offentlige transport. Dette gælder for de gange patienter fra Fyn og Sjælland skal komme til testcentrene på OUH og Taastrup til kontrol og tests. På turen til Taastrup dækkes desuden transportudgifter således at hver forsøgsdeltager kan få én pårørende med.

**Hvervning af deltagere.**
Projektet forventes at rekruttere patienter fra to geografiske områder dels fra Region Syddanmark dels Region Hovedstaden. Via kontakt til de respektive neurologiske afdelinger, praktiserende speciallæger i neurologi samt Dansk Parkinsonforenings lokalkredse vil parkinsonpatienter blive inviteret til informationsmøder med henblik på en senere inklusion i undersøgelsen. Der planlægges to inklusions centre dels på neurologisk afdeling OUH og Handicaporganisationernes Hus i Tåstrup.

**Tilgængeligheden af oplysninger for forsøgspersoner.**
Forsøgspersoner får udleveret kontaktoplysninger på relevant sundhedspersonale der kan assistere med at besvare de forespørgsler som forsøgsdeltagerne måtte have. Forsøgsdeltagerne vil have adgang til kontakt data på ansat projekt sygeplejerske samt forsøgsansvarlig. Den enkelte forsøgsdeltager vil i den aktive del af forsøget være tilknyttet en af ovenstående kontaktpersoner.

Forsøgsdeltagerne vil, såfremt at de har givet samtykke til dette jævnfør anvendte samtykkeerklæring fra det videnskabsetiske komite system, blive informeret om resultaterne af forsøget skriftligt i let læseligt sprog, når forsøget er afsluttet og det videnskabelige arbejde er færdiggjort.

**Offentliggørelse af forsøgsresultater.**
Resultaterne fra projektet forventes at bidrage med 2-4 videnskabelige artikler, der kan publiceres internationalt peer-reviewed, samt ved møder såvel nationalt, som internationalt. Resultaterne søges offentliggjort uanset om resultaterne er positive, negative eller inkonklusive. Der vil løbende under forsøget, når resultaterne tillader det, blive udarbejdet præliminære artikler og endelige artikler efter afslutningen af forsøget.

**Videnskabsetisk redegørelse.**
Studiet udføres i henhold til gældende dansk lovgivning med anmeldelse til Datatilsynet og Videnskabs Etisk Komité samt sundhedsstyrelsen. Opbevaring af data sker forsvarligt efter gældende regler. Det sikres at persondataloven overholdes.
Forsøgsprotokollen fra 2006 er tidligere godkendt af Videnskabsetisk Komité (journalnummer 01 278045) og af Datatilsynet, men der skal genansøges ligesom Sundhedsstyrelsen skal ansøges om tilladelse til brug af T-PEMF apparatet.
Såfremt en behandling mod Parkinsons sygdom skal være klinisk effektfuld og forsvarlig skal behandlingen leve op til at følgende kriterier opfyldes: behandlingen vil have en effekt på sygdommen herunder at behandlingen samlet set kan øge pateintens livskvalitet, acceptable bivirkninger ud fra patientsynspunkt og behandlersynspunkt.
T-PEMF har ved et tidligere forsøg med Parkinsons sygdom [26] demonstreret, der synes at være en effekt på livskvalitet og depression. Det er væsentligt at påvise om de resultater fundet ved det tidligere forsøg kan verificeres ved en større og randomiseret undersøgelse. Det er tillige væsentligt at få et indblik i, om patienter, som én gang har profiteret af T-PEMF kan gøre dette igen. Samt hvorvidt at parkinson-patienter via behandlingen kan få afhjulpet de motoriske funktioner og de non-motoriske symptomer af både behandlelig samt behandlingsrefraktær karakter.
Undersøgelser indikerer at bivirkningsprofilen er positiv med relativt beskedne og forbigående bivirkninger. Indtil nu har T-PEMF behandlinger, således som også beskrevet i afsnittet om bivirkninger, kun resulteret i meget få og kortvarige bivirkninger. Imidlertid er det af afgørende betydning at undersøge, om de foreløbige rapporter om bivirkninger er i overensstemmelse med den faktiske forekomst af bivirkninger eller hændelser som følge af T-PEMF behandling.
Da studiet designet bygger på anvendelse af en interventionsgruppe og en placebogruppe er det meget relevant at undersøge hvorvidt anvendelse af placebo udgør et etisk dilemma. For at undersøge om en behandlingsmetode er virksom mod en sygdom er det nødvendigt at anvende placebobehandling. I om med at der ved T-PEMF behandling ikke hidtil er set alvorlige bivirkninger, og der er videnskabelig evidens for at T-PEMF har en fysiologisk effekt som også vurderes at have symptomlindrende effekt hos patienter med Parkinsons sygdom skønnes det at det er etisk forsvarligt at anvende en placebogruppe i studiet. Endvidere stilles de patienter der er med i forsøget ikke dårligere i deres rettigheder i forhold til specialiseret behandling mod Parkinsons sygdom. Således vil alle deltagere både i interventionsgruppe og placebogruppen modtage behandling som alle andre parkinson-patienter.

Det vurderes ikke at deltagelse i projektet vil have en betydning for hvorledes patienter kan udføre dagligdags gøremål da T-PEMF giver minimal negativ indflydelse i dagligdagen.
Alle forsøgsdeltagere bliver informeret om, at de ved tilfældig udvælgelse bliver allokeret enten til en placebo gruppe eller interventionsgruppe. Forsøgsdeltagerne har således mulighed for at fravælge deltagelse i studiet.
Såfremt resultaterne af denne undersøgelse viser at der er en effekt vurderet ud fra subjektive og objektive kriterier samtidig med at der er en acceptabel bivirkningsprofil, indebærer dette at et muligt nyt behandlingstiltag for patienter med Parkinsons sygdom.

**Oplysning om erstatnings-eller godtgørelsesordninger.**
Forsøgsdeltagerne er dækket af patientforsikringsordninger der dækker de skader der måtte blive påført forsøgspersoner i forbindelse med sundhedsvidenskabelige forskningsprojekter. Forsøget udføres under direkte ansvar fra OUH.

**Protokolresumé.**

**Titel**
T-PEMF (Transkranielle Pulserende ElektroMagnetiske Felter) ved Parkinsons sygdom.

**Forsøgsansvarlig**
Bo Mohr Morberg Neurologisk afdeling OUH.

**Forsøgets formål**
Formålet er at undersøge, om 8 ugers behandling med elektromagnetiske felter der gives i hjernen via en hjelm kan mindske sværhedsgraden af symptomer patienter med Parkinsons sygdom oplever. Her tænkes der på forstyrrelser at patientens bevægelser og psykiske påvirkninger samt problemer med tale og vandladning.
Endvidere er formålet at afklare hvorvidt patienter med Parkinsons sygdom oplever en statistisk bedring af symptomerne ved behandling med aktiv T-PEMF, når de behandles dagligt i 8 uger i forhold til en gruppe der modtager inaktiv T-PEMF.

**Forsøgets metode**
Undersøgelsen med T-PEMF udføres som dobbeltblindet placebokontrolleret, det vil sige at hverken patient eller undersøger er vidende om hvem der modtager den aktive behandling eller den inaktive behandling. Halvdelen af de 120 parkinson-patienter udvælges tilfældigt til aktiv T-PEMF behandling via et randomiseringsprogram resten vil modtage inaktiv T-PEMF behandling.
Randomiseringen foregår ud fra randomiseringskriterierne: alder, køn, parkinsonstadiet (Hoehn and Yahr Scale) for at minimere fejlkilder i studiet. De aktive og inaktive T-PEMF apparater vil fremtræde identiske, og under behandlingen vil en kontrollampe blinke. Den inaktive behandling foregår uden dannelse af et magnetfelt. Deltagerne vil ikke under selve behandlingen kunne mærke om de modtager aktiv eller inaktiv behandling.
Patienterne må ikke omtale nogle forhold vedrørende apparatet eller behandlingen for undersøgerne. Overtrædelse af dette medfører udelukkelse fra forsøget. Alle procedurer inklusiv information og instruktion i brug af apparaterne udføres af sundhedsfagliguddannet personale.

De 120 patienter fordeles ligeligt henholdsvis i en behandlingsgruppe der modtager aktiv T-PEMF og en placebogruppe der modtager inaktiv T-PEMF behandling. Varigheden af behandlingen er 8 uger da nervevævet i hjernen tager tid om at forbedre sig. Grupper af 6 patienter vil indgå i forsøget med ca. 6 ugers mellemrum.
I forbindelse med projektet udtages en 5 ml veneblodprøve før og efter behandlingsinterventionen. Denne analyseres for BDNF som markør for nervevækst og for VEGF som markør for angiogenese. Blodprøver analyseres løbende og der oprettes ikke en biobank. Blodprøven tages to gange i studiet af den forsøgsansvarlige og analyseres straks og destrueres øjeblikkeligt herefter.
Patienterne skal være optimalt medicinsk behandlet før de indgår i studiet. Medicinen tilstræbes at blive holdt uændret under undersøgelsen.

**Statistiske overvejelser**
Resultaterne fra forsøget bearbejdes statistisk, interventionsgruppen og placebogruppens resultater sammenlignes. Statistikerfunktionen ved institut for regional Sundhedsforskning vil bidrage med vejledning. De statistiske beregninger vil fastslå validiteten af forsøgsresultaterne.
Statiske beregninger udført ud fra det ikke blindede pilotstudiets resultater, viste at 120 parkinson-patienter skal inkluderes i undersøgelsen, for at det kan forventes at vise en signifikant effekt på effektmålet livskvalitet (målt med PDQ-8). De 120 patienter fordeles ligeligt henholdsvis i en interventionsgruppe og en placebogruppe. For at opnå en virkning på de enzymatiske processer i hjernen, og dermed en klinisk effekt for patienten af behandlingen, forventes en behandlingsvarighed på mindst 4-5 uger med 1 daglig behandling, hvorfor 8 uger er valgt for at være sikker på at der opnås en maksimal effekt. Det estimeres at der skal være 60 projektdeltagere pr. center. I forhold de præliminære resultater fra effekten af T-PEMF på depression kan et deltager antal interventionsgruppen være 40-50 og stadigvæk vil kunne producere et validt statistisk datasæt. Der er lavet styrkeberegning for UPDRS der viser at ved en styrke på 90% og et signifikans niveau på 5% vil 84 deltagere være statistisk signifikant. Det vil sige 42 deltagere i placebogruppen og interventionsgruppen. Derfor vurderes det at en interventionsgruppe på 60 er sufficient for at sikre validiteten af data og samtidigt tage højde for dropouts der estimeres til 10%.

**Forsøgspersoner, herunder inklusions-og eksklusionskriterier**

Krav til forsøgsdeltagerne i forhold til deltagelse i forsøgsprojektet

*Følgende skal opfyldes for at komme med i forsøget:*

- Patienter med Idiopatisk Parkinsons sygdom H & Y 1-3 defineret efter standardkriterier.
- MMSE > 22 (anvendes til at screene for begyndende demensudvikling).
- Alder > 18 år.
- Patienten er i stand til at forstå, acceptere og gennemføre de planlagte procedurer.

*Hvis én af følgende emner opfyldes kan man ikke deltage i forsøget:*

- Patienten har på inklusionstidspunktet tidligere været i T-PEMF behandling.
- Der må ikke være ændringer af den medicin mod parkinson patienten modtager indenfor de sidste 6 uger.
- Udtalt demens eller anden hjerneskade, der kan influere på evnen til at afgive informeret samtykke, eller vanskeliggøre vurderingen af patientens tilstand.

- Sindslidelse der nødvendiggør anden intervention.
- Misbrug af alkohol eller stoffer.
- Pacemaker.
- I behandling med Deep Brain stimulation.
- Graviditet eller amning.
- Epilepsi.
- Aktive implantater i form af pacemakere og andre som f.eks. implantater i ørene.
- Aktivt medicinsk udstyr f.eks. medicinpumper og andet.
- Deltagelse i andre forsøg eller afprøvninger i projektperioden.
- Aktuel eller tidligere kræftsygdom i hjernen, leukæmi, modermærkekræft, pladecellekræft, eller andre kræftformer i hoved/hals region.
- Autoimmun sygdom.
- Åbne sår i hovedbund.

Der stilles ingen krav til patienternes køn og alder så længe at de opfylder kriterierne for at deltage.

**Bivirkninger, risici og ulemper**
Behandling med elektromagnetiske felter herunder T-PEMF er stort set uden gener eller bivirkninger. Dette bekræftes af tidligere undersøgelser der ikke har vist bivirkninger vurderet ved bivirkningsskalaen UKU (udvalg for kliniske undersøgelser). I undersøgelserne har antallet af behandlinger været mange og behandlingen har strakt sig over lang tid. Foreløbige rapporter om bivirkningsprofilen for T-PEMF behandling indikerer at behandlingerne kan give let og forbigående kvalme og hovedpine. I nogle tilfælde et ubehag i nakkemusklerne, grundet hjelmens vægt. Der er for nuværende ikke rapporteret om andre bivirkninger.
Da patienten kan modtage behandlingen derhjemme og kan varetage dagligdags funktioner mens de modtager behandlingen, samt at behandlingen har en varighed på 30 min 1 gang om dagen skønnes det ikke at behandlingen bliver til gene for patienten. Det er også vigtigt at understrege, at deltagelse i forsøget ikke har indflydelse på de rettigheder patienten har til behandling for sin Parkinsons sygdom. Det skønnes ikke at en veneblodprøve hvor der samlet udtages 10 ml. blod, foretaget af den forsøgsansvarlige med rutine i dette, vil være til væsentlig gene for forsøgsdeltageren.
Ved andre behandlinger med elektromagnetiske bølger af større styrke end dem der anvendes ved PEMF, har der ikke været observeret langtidsbivirkninger.
I undersøgelsens forløb registreres ved alle kontakter eventuelle hændelser med en angivelse af, om disse er alvorlige eller mindre alvorlige, samt en angivelse af om de ifølge undersøgerens skøn er relateret til T-PEMF behandlingen. I tilfælde af alvorlige uønskede hændelser, hvor T-PEMF behandlingen er medvirkende årsag til hændelsen, vil den lokale Videnskabsetiske komité omgående blive underrettet.

Endvidere vil Sundhedsstyrelse, den lokale Videnskabsetiske komité og styregruppen ligeledes blive orienteret om alle alvorlige hændelser og næsten-hændelser, som er indtruffet i perioden som helhed, samt give oplysning om forsøgspersonernes sikkerhed. Ved alle hændelser forstås både de forventede og uventede hændelser.

**Økonomiske forhold**
Den forsøgsansvarlige og styregruppen modtager ingen vederlag for gennemførelse af forsøget. Hverken den forsøgsansvarlige eller styregruppen har økonomisk interesse i det firma der leverer apparaterne. Endvidere har den forsøgsansvarlige og styregruppen ingen tilknytning til støttegiveren DEN A.P. MØLLERSKE STØTTEFOND.

Initiativet til forskningsprojektet er taget af en styregruppe bestående af:

Styregruppen: Overlæge, klinisk lektor Lene Wermuth (Ph.d.-vejleder)
Overlæge, dr. med Ole Gredal
Lektor, ph.d. Bente Rona Jensen (Ph.d.-vejleder)
Professor, dr.med. Per Bech (Ph.d.-vejleder)
Læge Bo Mohr Morberg (forsøgsansvarlig)

Der foreligger økonomisk bevilling til såvel gennemførelse af projektet samt Ph.d. forløb fra Den A. P. Møllerske Støttefond.

Det kliniske studie ledes af Neurologisk forskningsenhed OUH.

Støtten udbetales som en fast sum og der oprettes to forskningskonti i OUH regi der vil være økonomisk administrator. Én konto der dækker lønomkostninger til den forsøgsansvarlige og én konto der dækker omkostninger til øvrig drift.

Et eventuelt overskydende støttebeløb vil blive tilbageført til fonden.

**Offentliggørelse af forsøgsresultater**
Resultaterne fra projektet forventes at bidrage med 2-4 videnskabelige artikler, der kan publiceres internationalt, samt ved møder såvel nationalt, som internationalt. Resultaterne søges offentliggjort uanset om resultaterne er positive, negative eller inkonklusive. Der vil løbende under forsøget når resultaterne tillader det blive udarbejdet data til fremlæggelse i offentlig regi og endelige artikler efter afslutningen af forsøget.

**Videnskabsetisk redegørelse.**
Studiet udføres i henhold til gældende dansk lovgivning med anmeldelse til Datatilsynet og Videnskabs Etisk Komité samt sundhedsstyrelsen. Opbevaring af data sker forsvarligt efter gældende regler. Det sikres at persondataloven overholdes.
Forsøgsprotokollen fra 2006 er tidligere godkendt af Videnskabsetisk Komité (journalnummer 01 278045) og af Datatilsynet, men der skal genansøges ligesom Sundhedsstyrelsen skal ansøges om tilladelse til brug af PEMF apparatet.

Såfremt en behandling mod Parkinsons sygdom skal være klinisk effektfuld og forsvarlig skal behandlingen leve op til at følgende kriterier opfyldes: behandlingen vil have en effekt på sygdommen herunder at behandlingen samlet set kan øge patientens livskvalitet, acceptable bivirkninger ud fra patientsynspunkt og behandlersynspunkt.
T-PEMF har ved et tidligere forsøg med Parkinsons sygdom [18] demonstreret, der synes at være en effekt på livskvalitet og depression. Det er væsentligt at påvise, at dette fund kan genfindes ved en ny undersøgelse. Det er tillige væsentligt at få et indblik i, om patienter, som én gang har profiteret af T-PEMF kan gøre dette igen. Samt hvorvidt at parkinson-patienter via behandlingen kan få afhjulpet de motoriske og non-motoriske symptomer af både behandlelig samt behandlingsrefraktær karakter.
Undersøgelser indikerer at bivirkningsprofilen er positiv med relativt beskedne og forbigående bivirkninger. Indtil nu har T-PEMF behandlinger, således som også beskrevet i afsnittet om bivirkninger, kun resulteret i meget få og kortvarige bivirkninger. Imidlertid er det af afgørende betydning at undersøge, om de foreløbige rapporter om bivirkninger er i overensstemmelse med den faktiske forekomst af bivirkninger eller hændelser som følge af T-PEMF behandling.
Da studiet designet bygger på anvendelse an en interventionsgruppe og en placebogruppe er det meget relevant at undersøge hvorvidt anvendelse af placebo udgør et etisk dilemma. For at undersøge om en behandlingsmetode er virksom mod en sygdom er det nødvendigt at anvende placebobehandling. I om med at der ved T-PEMF behandling ikke hidtil er set alvorlige bivirkninger, og der er videnskabelig evidens for at T-PEMF har en fysiologisk effekt som også vurderes at have symptomlindrende effekt hos patienter med Parkinsons sygdom skønnes det at det er etisk forsvarligt at anvende en placebogruppe i studiet. Endvidere stilles de patienter der er med i forsøget ikke dårligere i deres rettigheder i forhold til specialiseret parkinson behandling som helhed. Således vil alle deltagere både i interventionsgruppe og placebogruppen modtage behandling som alle andre parkinson-patienter.
Det vurderes ikke at deltagelse i projektet vil have en betydning for hvorledes patienter kan udføre dagligdags gøremål da T-PEMF giver minimal negativ indflydelse i dagligdagen.
Alle forsøgsdeltagere bliver informeret om, at de ved tilfældig udvælgelse bliver allokeret enten til en placebo gruppe eller interventionsgruppe. Forsøgsdeltagerne har således mulighed for at fravælge deltagelse i studiet.
Såfremt resultaterne af denne undersøgelse viser at der er en effekt vurderet ud fra subjektive og objektive kriterier samtidig med at der er en acceptabel bivirkningsprofil, indebærer dette at et muligt nyt behandlingstiltag for patienter med Parkinsons sygdom.

**Hvervning af deltagere**
Projektet forventes at rekruttere patienter fra to geografiske områder dels fra Region Syddanmark dels Region Hovedstaden. Via kontakt til de respektive neurologiske afdelinger, praktiserende speciallæger i neurologi samt Dansk Parkinsonforenings lokalkredse vil parkinsonpatienter blive inviteret til informationsmøder mhp en senere inklusion i undersøgelsen. Der planlægges 2 inklusions centre dels på neurologisk afd. OUH og Handicaporganisationernes Hus i Høje Tåstrup.

**Mundtlig deltagerinformation**
Den mundtlige information gives af den forsøgsansvarlige. Kontakten til mulige forsøgsdeltagere er

formidlet via brev indeholdt den skriftlige deltagerinformation, hvor de inviteres til et fælles mundtligt informationsmøde. Forsøgsdeltagerne har mulighed for at tage en pårørende med til informationsmødet som bisidder. De deltagere der accepterer at være med i forsøget vil modtage individuel mundtlig information inden de underskriverinformeret samtykke erklæringen.

**Følgende vil være indholdet i invitationen(udover indholdet af skriftlig deltagerinformation)**

- Dato og tid for informationsmødet
- Der informeres om at indholdet er vedrørende en forespørgsel om deltagelse i et sundhedsvidenskabeligt forskningsprojekt.
- Der informeres om at det er muligt for patienten at tage en bisidder med til mødet.

Der gøres opmærksomt på at der er efter informationsmødet vil være en betænkningstid på 2 dage til at tage stilling til om hvorvidt man ønsker at være med i projektet, samt at der ved accept på at være forsøgsdeltager vil være et individuelt informationsmøde med den forsøgsansvarlige hvor endelig accept gives fra forsøgsdeltageren. Således at der indhentes samtykke efter skriftlig og individuel mundtlig information.

Selve informationsmødet afholdes en et lokale på afdeling N OUH og i et lokale i Handicaporganisationernes Hus i Taastrup. Mødet afholdes sidst på eftermiddagen således at der er færrest mulige personer på afdelingen og i Handicaporganisationernes Hus derved sikres at forstyrrelser minimeres. Mødetidspunktet tilrettelægges ikke senere end deltagerne skønnes at være kognitivt friske. Der vil være forfriskninger til deltagerne på informationsmødet.

Efter det mundtlige informationsmøde kan patienterne tage hjem og tale med pårørende om hvorvidt patienten ønsker at deltage i forsøget. Patienten får samtykkeerklæringen fra Videnskabsetisk Komité udleveret til det mundtlige informationsmøde, og kan således tage det med hjem i betænkningsfasen efter informationsmødet. Der forventes at der vil være 2 dages betænkningstid. Forsøgsdeltagerne får udleveret kontakt data på den forsøgsansvarlige således at deltagerne kan kontakte denne ved tvivlsspørgsmål.

Deltagere vil få tilsendt den skriftlige deltagerinformation på skrift senest 14 dage ingen mødet således at der er god tid til at få læst materialet igennem således at mulige spørgsmål kan tages med til mødet. Patienterne skal melde tilbage senest 7 dage før mødet om de ønsker at deltage således at forplejning og det rette lokale størrelse kan bookes. Der vil efter mødet være mulighed for at tale med den forsøgsansvarlige under mere uformelle rammer såfremt at nogle patienter skulle ønske dette.

Informationen leveres via let forståelige og let læselige PowerPoint slides som også udleveres til deltagerne i starten af mødet. Således vil lix tallet være tilpasset patientgruppen samt vil der ikke være lægefagtermer, disse vil være oversat til almindelig dansk således tilpasset til denne patientgruppes formodede demografi.

Der vil blive informeret om mulige bivirkninger, muligheden for uforudsete hændelser, mulige ulemper ved deltagelse i forsøget, og at der stilles krav til deltagerne i forsøget i forhold til compliance.

Deltagerne i forsøget vil få gennemlæst deres journal i forhold til aktuel behandling samt andre sygdomme. Deltagerne vil få at vide, at de deltager i et videnskabeligt forsøg der kan afgøre om der

i fremtiden vil være en ny behandlingsmetode til dem.

Ved informeret samtykke anvendes skema fra Videnskabsetisk Komité.

Endvidere vil der i henhold til loven om medicinsk udstyr i tilfælde af inspektion gives fuldmagt til Sundhedsstyrelsen med henblik på at få adgang til patientens journal.

Findes der i løbet af forsøget informationer om effekten af behandlinger, bivirkninger fra behandlingen eller komplikationer vil forsøgsdeltagerne blive informeret. Endvidere vil forsøgsdeltagerne blive informeret hvis studiets design ændres væsentligt hvis det skønnes relevant for forsøgedeltagernes sikkerhed.

Forsøgsdeltageren vil såfremt denne ønsker det, modtage informationen om sin helbredstilstand såfremt der under forsøget fremkommer væsentlige oplysninger om denne.

Forsøgsdeltageren, vil såfremt denne ønsker dette, efter afslutning af forsøget blive informeret om resultaterne af studiet og de mulige konsekvenser dette kunne afføre for deltageren.

Såfremt at forsøges afbrydes før tid vil forsøgedeltageren blive informeret om årsagen hertil.

Væsentlige helbredsoplysninger vil blive meddelt til forsøgsdeltagerne.

Litteraturliste

[1]. Parkinsons sygdom. Klinisk vejledning 2 udgave. Danmodis; 2011
[2]. Lees AJ, Hardy J, Revisz T. Parkinson's disease. Lancet 2009;373:2055-6
[3]. Bretlau L, Lunde MA, Lindberg L, Unden M, Bech P. Repetitive Transcranial Magnetic Stimulation (rTMS) in combination with escitalopram in patients with treatment-resistant major depression. A double-blind, randomised, sham-controlled trial. Pharmacopsychiatry. 2008;41:41-47
[4]. Martiny K et al. Pulsed electromagnetic fields (PEMF) in treatment of resistant depression. A pilot study. International Society of Affective Disorders (Abstract). Second Biennial International Conference. Cancun, Mexico March 5th -10th, 2004
[5]. Centonze D, Koch G, Versace V, Mori F, Rossi S, Brusa L, et al. Repetitive transcranial magnetic stimulation of the motor cortexameliorates spasticity in multiple sclerosis. Neurology 68, 1045-1050, 2007
[6]. Krause P, Edrich T, Straube A. Lumbar repetitive magnetic stimulation reduces spastic tone
increase of the lower limbs. Spinal cord 42, 67-72. 2004
[7]. Nielsen J.F, Sinkjaer T, Jakobsen J. Treatment of spasticity with repetitive magnetic stimulation; a double-blind placebo-controlled study. Mult Scler 2, 227-32,1996
[8]. Sherafat MA, Heibatollahi M, Mongabadi S, Moradi F, Javan M, Ahmadiani A. Electromagnetic Field Stimulation Potentiates Endogenous Myelin Repair by Recruiting Subventricular Neural Stem Cells in an Experimental Model of White Matter Demyelination. J Mol Neurosci.2012 Sep;48(1):144-53
[9] Borsalino G, Bagnacani M, Bettati E et al. Electrical stimulation of human femoral intertrochanteric osteotomies. Double-blind study. Clin Orthop Relat Res 1988; (237):256-63
[10] Sandyk R. Speech impairment in Parkinson's disease is improved by transcranial application of electromagnetic fields. Int J Neurosci.1997 Nov;92(1-2):63-72
[11] Sandyk R. Treatment with electromagnetic fields improves dual-task performance (talking while walking) in multiple sclerosis. Int J Neurosci.1997 Nov;92(1-2):95-102
[12]. Arias-Carrión O. Basic mechanisms of rTMS: Implications in Parkinson's disease. Int Arch Med.2008 Apr 15;1(1):2
[13]. Randhawa BK, Farley BG, Boyd LA. Repetitive transcranial magnetic stimulation improves handwriting in Parkinson's disease. Parkinsons Dis. 2013;2013:751925
[14]. Martiny K, Lunde M, Bech P. Transcranial low voltage pulsed electromagnetic fields in patients with treatment-resistant depression. Biol Psychiatry. 2010 Jul 15;68(2):163-9. doi: 10.1016/j.biopsych.2010.02.017. Epub 2010 Apr 10
[15].Bech P, Gefke M, Lunde M, Lauritzen L, Martiny K. The pharmacopsychometric triangle to illustrate the effectiveness of T-PEMF concomitant with antidepressants in treatment resistant patients: A double-blind, randomised, sham-controlled trial revisited with focus on the patientreported outcomes. Depression Research and Treatment. Volume 2011, Article ID 806298, 6 pages
[16]. Andreasson K, Liest V, Lunde M, Martiny K, Unden M, Dissing S et al. Identifying patients with therapy-resistant depression by using factor analysis. Pharmacopsychiatry 2010;43:252-256

[17]. Santos-García Det al. Impact of non-motor symptoms on health-related and perceived quality of life in Parkinson's disease. J Neurol Sci.2013 Jul 25. pii: S0022-510X(13)00316-X
[18]. Ulrik L Rahbek, Katerina Tritsaris, Steen Dissing. Interaction of Low-frequency, Pulsed Electromagnetic Fields with Living Tissue: Biochemical Responses and Clinical Results. Vol 2. No 1. 2005. Oral Biosciences & Medicine. 1-12
[19]. Oren M. Tepper, Matthew J. Callaghan, Edward I. Chang, Robert D. Galiano, Kirit A. Bhatt, Samuel Baharestani, et al. Electromagnetic fields increase in vitro and in vivo angiogenesis through endothelial release of FGF-2. The FASEB Journal express article10.1096/fj.03-0847fje. Published online June 18, 2004
[20] Macias MY, Battocletti JH, Sutton CH, Pintar FA, Maiman DJ.Directed and enhanced neurite growth with pulsed magnetic field stimulation. Bioelectromagnetics, 21 (2000), pp. 272–286
[21] Longo FM, Yang T, Hamilton S, Hyde JF, Walker J, Jennes L et al. Electromagnetic fields influence NGF activity and levels following sciatic nerve transection. J Neurosci Res 1999, 55:230-237
[22] Macias MY, Battocletti JH, Sutton CH, Pintar FA, Maiman DJ. Directed and enhanced neurite growth with pulsed magnetic field stimulation. Bioelectromagnetics 2000, 21:272-286
[23] Sisken BF, Kanje M, Lundborg G, Herbst E, Kurtz W. Stimulation of rat sciatic nerve regeneration with pulsed electromagnetic fields. Brain Res 1989. 485:309-316
[24] Pezetti F, De MM, Caruso A, Cadossi R, Zucchini P, Carinci F. Effect of pulsed electromagnetic fields om human chodrocytes: an in vitro study. Calcif Tissue Int 1999, 65:396-401
[25] SmithTL, Wong-Gibbons D, Maultsby J. Microcirculatory effects of pulsed electromagnetic fields. J Orthop Res. 2004Jan;22(1):80-4.
[26]. Dethlefsen G, Gredal O, Wermuth L. Effect of pulsed electro magnetic fields in patients with idiopathic Parkinsons disease: A pilot study. Fremlagt som poster ved ”8th International Conference on Alzheimer's and Parkinson's Diseases AD/PD. March 14-18, 2007, Salzburg, Austria
[27]. Martiny K, Lunde M, Bech Transcranial low voltage pulsed electromagnetic fields in patients with treatment-resistant depression. Psychiatry. 2010 Jul 15;68(2):163-9. doi: 10.1016/j.biopsych.2010.02.017. Epub 2010 Apr 10.

Skriftlig deltagerinformation:

Odense d. xxx

Kære fulde navn

Jeg tillader mig hermed at kontakte dig vedrørende deltagelse i et forsøgsprojekt omhandlende Parkinsons sygdom og behandling med elektrisk feltterapi forkortet T-PEMF.

Der anmodes om lov til at spørge om du vil deltage i et videnskabeligt forsøgsprojekt vedrørende elektrisk feltterapi behandling til patienter med Parkinsons sygdom.

For alle interesserede vil der blive afholdt et fælles informationsmøde. Såfremt man vælger at deltage i forsøget vil der inden at du afgiver endelig accept blive afholdt et individuelt informationsmøde for dig og din pårørende med den forsøgsansvarlige.

For praktiske spørgsmål vedrørende det mundtlige informationsmøde og spørgsmål til forsøget se venligst sidste side i dette brev.

Information om forsøget kan læses herunder

Titel: Behandling af hjernen med elektromagnetiske felter til patienter med Parkinsons sygdom.

**Transkranielle Pulserende ElektroMagnetiske Felter (T-PEMF) ved Parkinsons sygdom (PD).**
Forskning på dette område er meget vigtig, da det undersøges om elektromagnetisk behandling kan opretholde og om muligt forøge mængden af dopamin i hjernen. Resultaterne fra studiet vil kunne være med til at forbedre de symptomer patienter med Parkinsons sygdom har i både bevægeapparatet og af psykisk karakter; vandladningsgener og søvnbesvær. Forskning på området tegner et billede af, at elektromagnetiske felter kan medvirke til, at der går længere tid før man får symptomer på Parkinsons sygdom samt at hjernen kan ændre sig positivt ved behandling med elektromagnetiske felter. Dette forsøg vil bidrage til denne forskning og til fremtidig behandling patienter med Parkinsons sygdom. Du kan være medvirkende til at der forskes yderligere på området,og at det muligvis vil give adgang for en ny type behandling til patienter med Parkinsons sygdom.
Som deltager i forsøget vil du blive udvalgt tilfældigt til enten at modtage aktiv behandling eller inaktiv behandling. De forsøgsdeltagere der kommer i aktiv gruppen vil kunne drage nytte af at være med i forsøget, da det skønnes at behandlingen vil have positiv effekt på parkinson symptomer.
Behandling med elektromagnetiske felter herunder T-PEMF er stort set uden gener eller bivirkninger. Dette bekræftes af tidligere undersøgelser der ikke har vist bivirkninger. I undersøgelserne har antallet af behandlinger været mange og behandlingen har strakt sig over lang tid. Ved andre behandlinger med elektromagnetiske bølger af større styrke end dem der anvendes ved T-PEMF, har der ikke været observeret langtidsbivirkninger. Foreløbige rapporter om bivirkninger for T-PEMF behandling tyder på at behandlingerne kan give let og forbigående kvalme og hovedpine og i nogle tilfælde et ubehag i nakkemusklerne, grundet hjelmens vægt. Der er for nuværende ikke kendskab til andre bivirkninger.

Behandlingen foregår hjemme hos dig selv og forløber i 8 uger. Under behandlingen vil du kunne varetage dagligdags funktioner af rolig karakter for eksempel læse en bog, se fjernsyn, løse kryds og tværs eller strikke. Behandlingen har en varighed på 30 min éngang om dagen. Selve apparatet er udformet som en hjelm, se vedlagte foto, der bæres på hovedet de 30 min dagligt som behandlingen varer. Hjelmen skal opbevares sådan at den ikke udsættes for slag, for eksempel i et skab, når den ikke anvendes. Du vil blive grundigt vejledt i anvendelsen af apparatet ved udlevering af dette. Der vil endvidere blive taget telefonisk kontakt ugentligt til dig for at sikre at apparatet kan anvendes. Du vil få udleveret kontakt data på den forsøgsansvarlige således at du frit kan kontakte denne ved problemer under behandlingen. Det skønnes at behandlingen ikke vil blive til gene for dig. Det er vigtigt at understrege at deltagelse i forsøget ikke har indflydelse på de rettigheder du har til behandling for din Parkinsons sygdom. Der vil i forbindelse med udlevering af apparatur og test skulle påregnes transport til København to gange i løbet af forsøget samt tre gange til OUH når du skal testes. Transportudgifter svarende til billigste offentlige transport eller som kørselsgodtgørelse ved transport i privat bil dækkes af forsøgsmidler. Der dækkes også økonomisk for transport af én pårørende. Der vil blive taget 2 blodprøver i løbet af forsøget for at se hvorledes behandlingen påvirker hjerne og blodkar. Mængden af blod der udtages er på samlet 10 ml hvilket i mængde svarer til at få taget en blodprøve hos egen læge. Blodprøverne tages af den forsøgsansvarlige der har rutine i blodprøvetagning, og vil blive destrueret efter de er analyseret.

Forsøget har muligheden for at give en nytteværdi til fremtidens Parkinson patienter. Hvis forsøget viser en betydelig effekt af elektromagnetisk feltterapi vil dette kunne være med til at danne grundlag for den fremtidige behandling af Parkinson patienter.

Du kan udelukkes fra behandlingen såfremt du ikke har været ærlig omkring de kriterier der skal opfyldes ved indlemmelse i forsøget. Du kan ligeledes udelukkes fra forsøget, såfremt at du skulle blive så mentalt svækket, at du ikke kan tage ansvar for egen behandling. Ligeledes vil du blive udelukket såfremt apparatet misvedligeholdes eller udlånes til anden part. Hvis du skulle blive alvorligt syg i forsøgsperioden kan du blive udelukket fra forsøget. Som deltager i forsøget må du ikke omtale nogle forhold vedrørende apparatet eller behandlingen for undersøgerne. Overtrædelse af dette medfører at du ikke længere kan være med i forsøget. Såfremt at du er kvinde i den fødedygtige alder kræves det for deltagelse i forsøget at du anvendersikker prævention. Såfremt dette er tilfældet vil du blive informeret nærmere herom af den forsøgsansvarlige, der følger Sundhedsstyrelsens anbefalinger for sikker prævention.
Forsøget kan afbrydes hvis det ikke længere menes sandsynligt at den indsamlede mængde data giver et validt forsøgsresultat, eller hvis der opstår uventede alvorlige bivirkninger til behandlingen.

Din journal vil blive gennemset med hensyn til opfyldelse af inklusionskriterier samt medicinering, din almene helbredstilstand og din Parkinsons sygdom. Ved din deltagelse i forsøget søges der at den medicinske behandling er stabil så længe at behandlingen pågår i de 8uger. Såfremt du skulle opleve symptomer på overdosering vil din medicinske behandling blive ændret.

Ved at du giver samtykke til deltagelse i forsøget giver du tilladelse til adgang til videregivelse og behandling af nødvendige oplysninger om dit helbred fra patientjournalen, øvrige rent private forhold og andre fortrolige oplysninger som led i sponsors og myndigheders kvalitetskontrol og monitorering.

Din journaloplysninger og personlige data vil blive behandlet i henhold til persondataloven og loven om tavshedspligt.

Initiativet til forskningsprojektet er taget af en styregruppe bestående af:

Styregruppen: Overlæge, klinisk lektor Lene Wermuth (Ph.d.-vejleder)
Overlæge, dr. med Ole Gredal
Lektor, ph.d. Bente Rona Jensen (Ph.d.-vejleder)
Professor, dr.med. Per Bech (Ph.d.-vejleder)
Læge Bo Mohr Morberg (forsøgsansvarlig)

Der foreligger økonomisk bevilling til såvel gennemførelse af projektet samt Ph.d. forløb fra kommer alene fra DEN A. P. MØLLERSKE STØTTEFOND. Der udbetales driftsomkostninger til den forsøgsansvarlige og drifts omkostninger til den resterende del af projektet. Projektet er forankret i neurologisk afdeling OUH. Der vil kører to studie et i København og et i Odense men begge hører under OUH og der er kun 1 forsøgsansvarlig på begge centre. Den forsøgsansvarlige og styregruppen er ikke tilknyttet DEN A.P MØLLERSKE STØTTEFOND og arbejder således uafhængigt af denne. Fonden har bevilliget kr. 4.962.000til gennemførelse af forsøget. Du kan få adgang til budget såfremt dette ønskes.

Den forsøgsansvarlige og styregruppen modtager ingen vederlag for gennemførelse af forsøget. Hverken den forsøgsansvarlige eller styregruppen har økonomisk interesse i det firma der leverer apparaterne. Endvidere har den forsøgsansvarlige og styregruppen ingen tilknytning til støttegiveren DEN A.P. MØLLERSKE STØTTEFOND.

Forsøgsansvarlig Bo Mohr Morberg vil stå til rådighed ved yderligere spørgsmål og generel information om forsøget.

Kontakt oplysninger:
Bo Mohr Morberg
Adresse: Kløvervænget 24 b st. 2 5000 Odense C
Mail: bomohrmorberg@gmail.com
Mobil: 28746323

Du opfordres til at læse det vedhæftede tillæg, ”Forsøgspersonens rettigheder i et sundhedsvidenskabeligt forskningsprojekt”,

Oplysninger om tid og sted for mundtligt informationsmøde XXXXX

Du opfordres til at tage en pårørende med til informationsmødet og skrive eventuelle spørgsmål ned som du måtte have og tage dem med til informationsmødet, eller kontakte den forsøgsansvarlige via mail eller telefon inden mødet.

Med venlig hilsen

Læge og forsøgsansvarlig

Bo Mohr Morberg

# Revised edition of the Danish study protocol (S-20130114)

Version of Dec 4th 2015

Approved by The Regional Scientific Ethical Committees for Southern Denmark Dec 8th 2015

Forsøgsprotokol:

## Forsøgets formål:

Formålet med forsøget er at undersøge, om 8 ugers behandling med transkranielle pulserende elektromagnetiske felter (T-PEMF) kan reducere sværhedsgraden af såvel de motoriske som non-motoriske symptomer hos patienter med idiopatisk Parkinsons sygdom (IPD).

Endvidere er formålet, at afklare hvorvidt patienter med Idiopatisk Parkinsons sygdom oplever en klinisk relevant og statistisk signifikant effekt af den aktive T-PEMF behandling når de behandles dagligt i 8 uger, i forhold til en gruppe der modtager inaktiv T-PEMF.

I forsøget anvendes 3 forskellige effektmål: Kliniske, neuropsykologiske og motoriske. Disse gennemgåes senere i protokollen.

## Hypotese

Vi forventer, af behandlingen med aktiv T-PEMF er signifikant bedre end placebo T-PEMF i forhold til at nedsætte sværhedsgraden af de motoriske og non-motoriske symptomer, som patienter med Parkinsons sygdom oplever. Vi forventer, at regressionen af symptomerne vil kunne give patienterne en markant bedre livskvalitet. Vi forventer, at kunne få et validt statisk materiale der kan understøtte vores hypotese.

## Baggrund og litteratur

## Parkinsons sygdom

Parkinsons sygdom (PD) er en fremadskridende neurodegenerativ sygdom, hvor der sker et omfattende tab af dopaminproducerende nerveceller i bestemte områder i hjernen, specielt i substantia nigra. Årsagen til idiopatisk PD kendes ikke, men det formodes at den skyldes en kombination af genetisk disposition og udefra kommende faktorer som eksempelvis livsomstændigheder, opvækstvilkår, påvirkning fra pesticider, tungmetaller og andre mulige toksiske stoffer. Der er påvist en familiær ophobning af PD i visse familier, specielt hvis sygdommen debuterer meget tidligt, før 40 års alderen. På nuværende tidspunkt er der beskrevet mere end 10 forskellige ændringer i arveanlæggene, som alle kan medføre at sygdommen kommer til udtryk. Der er en livslang risiko på 1 ‰ for at få PD, den stiger i takt med alderen (mere end 3 % i alderen +85) [1]. Gennemsnitsalderen for en nydiagnosticeret parkinson-patient er 60 år. Patienterne har et gennemsnitligt sygdomsforløb på mere end 15 år, med kun lidt reduceret livslængde i forhold til baggrundsbefolkningen. Parkinson-patienter dør ofte af følgesygdomme som for eksempel lungebetændelse [2]. Da patienterne ofte skal leve mange år med sygdommen, vil en påvirkning af behandlingen og dermed sygdomsforløbet være essentiel for livskvaliteten.

Den nedsatte dopaminproduktion i hjernen er ofte fremskreden, når de typiske symptomer fra det motoriske nervesystem, i form af bradykinesi/akinesi (langsom/manglende bevægelse) ledsaget af muskulær rigiditet, stivhed og hviletremor, præger sygdomsbilledet. Behandlingen af symptomerne ved PD er symptomatisk med dopaminerge lægemidler.

Sygdomsforløbet ved PD er progressivt, og der sker en aftagende virkning af den medicinske behandling efter års terapi. Patienterne oplever den aftagende effekt af medicinen som svingninger i symptombilledet. For nogle patienter ses en tendens til svingninger mellem dyskinesier (ufrivillige bevægelser) som tegn på overdosering af medicin, og parkinson-symptomer, som tegn på manglende effekt. Den medicinske behandling har store konsekvenser for parkinson-patientens motoriske funktionsniveau, og en forværring af PD manifesterer sig ved reduceret gangfunktion, faldtendens og

nedsat finmotorik, alle forhold der påvirker ADL-funktioner.

De relativt få parkinsonpatienter med mere komplicerede motoriske symptomer kan behandles med Deep Brain Stimulation (f.eks. tremor-dominant Parkinsons sygdom eller patienter med svære svingninger i behandlingseffekt). En anden behandlingsmetode for disse patienter er anlæggelse af en mavesonde. Ved denne behandlingsmetode gives den dopaminerge medicin direkte ind i tarmen via en pumpe, således at det motoriske funktionsniveau udjævnes (on-off Parkinson, der veksler mellem ufrivillige bevægelser og Parkinson-symptomer).

Parkinson-patienter vil, sammen med de motoriske symptomer, have non-motoriske symptomer. Disse symptomer kan være meget forskellige fra patient til patient men ligeså invaliderende som de motoriske symptomer. De non-motoriske symptomer omfatter påvirkning af det autonome nervesystem med udvikling af for eksempel ortostatisk hypotension, obstipation, vandladningsgener, impotens samt en mere udbredt påvirkning af hjernens generelle funktion, der medfører træthed, søvnforstyrrelser, depression, hallucinationer og demensudvikling. De non-motoriske symptomer responderer ikke på samme gunstige måde på den dopaminerge behandling, og derfor kan en række andre farmaka være indiceret, for eksempel tillæg af anti-depressiv medicin og demensmedicin. Denne polyfarmaci giver selvsagt øget risiko for interaktioner og bivirkninger. Selv med adækvat medicinsk behandling kan de non-motoriske symptomer være behandlingsrefraktære.

Parkinson-patienter beretter at de har nedsat helbredsrelateret livskvalitet. Livskvaliteten forringes ved forværring af de motoriske og non-motoriske symptomer. PD kan således have stor indflydelse for patientens og dennes pårørendes livsomstændigheder.

**Anvendelse af pulserende elektromagnetiske felter (PEMF) i lægevidenskaben**

Der har i litteratursøgningen været lagt vægt på at undersøge den fysiologiske virkning af T-PEMF, samt at undersøge i hvilke patologiske tilstande T-PEMF anvendes i klinikken herunder osteoartrose samt begyndende mod depression. Endvidere er der søgt litteratur på studier vedrørende TMS der grundlæggende anvender de samme behandlingsprincipper som T-PEMF.

Magnetstimulation anvendes både indenfor psykiatrien og neurologien til diagnostik og behandling. Neurologien har anvendt transkraniel magnetisk stimulation (TMS) til undersøgelse af nerveledningshastighed. Magnetteknologi er også basis for anvendelsen af magnetskanneren (MR-scanner) til visualisering af indre organer. Ved transkraniel magnet stimulation (TMS) appliceres via en spole hurtige ændringer i et magnetfelt over det ønskede stimulationsområde hvorved større elektriske felter udløses i hjernen. I psykiatrien er TMS ved at vinde indpas i den kliniske hverdag i behandlingen af depressive lidelser [3-4], i neurologien er der påvist dokumenteret effekt af TMS på spasticitet [5-7]. Da apparaturet til TMS ikke er flytbart kan behandlingen kun gives i hospitalsregi og på grund af den korte virkningsvarighed, til f.eks. spasticitet, skal den gives flere gange om ugen for at være effektiv.

Transkranial pulserende elektromagnetisk feltbehandling (benævnt T-PEMF) er på mange måder sammenlignelig med TMS-behandling. Men hvor TMS metoden anvender kraftige pulserende elektriske felter nær en styrke som de der udløser aktionspotentialer i nerver, så bygger den her anvendte T-PEMF teknologi på anvendelsen af elektriske felter som er mange størrelsesordner mindre i styrke og ikke ændrer cellers excitabilitet. T-PEMF udnytter pulser på 50 Hz i længere tid, typisk 30 min, medens TMS metoden til sammenligning anvender få pulser. T-PEMF behandlingen er baseret på en aktivering af hjernebarken ved hjælp af små pulserende elektriske felter af en størrelse og med en frekvens, som er vist at aktivere en række celletyper og enzymatiske processer blandt andet i hjernevæv.

Elektromagnetiske felter (EMF) har vist effekt på endogene neurale stamceller i hjernen, hvor det ser ud som om EMF kan potentiere proliferation og migration af neurale stamceller og ændre reparationen af myelin ved demyelisering [8]. PEMF er endvidere undersøgt ved knogleheling [9] i klinikken for Parkinsons sygdom [10] og multiple sclerose [11] Ved anvendelse af repetitive Transkranial magnetisk stimulation (rTMS) til Parkinsons sygdom undersøgt i dyrestudier synes dette at kunne ændre neurotransmitter frigørelse, synapse effekt og kan inducere neurogenese [12]. Anvendelse af 5 Hz rTMS til Parkinsonpatienter givet over supplementary motor area (SMA) viser effekt og bedring af håndskriften. Denne effekt forklares som ændring af den corticale excitability i det neurale netværk [13]. Til patienter med depression er der i Danmark gennemført et klinisk studie på 50 patienter med behandlingsrefraktær depression, hvor behandlingen med T-PEMF var eklatant i forhold til placebo-behandlingen[14].

En re-analyse af dette T-PEMF arbejde [15] viser, at effekten ikke alene var på de egentlige depressionssymptomer, men også på de symptomer man ser ved den medicinsk terapiresistente patient, nemlig træthed, manglende initiativ, koncentrationsforstyrrelser og søvnforstyrrelser [16]. Disse non-motor symptomer er også dominerende hos parkinsonpatienter og har stor betydning for deres livskvalitet [17].

Der er belæg for at PEMF har en biologisk effekt på forskellige cellulære niveauer. Den biologiske virkningsmekanisme ved T-PEMF er specielt studeret på endothelcellerne i blodhjernebarrieren [18]. Endvidere synes T-PEMF at kunne øge angiogenese [19], neurit vækst [20], øge perifer nerve regeneration [21-23], proliferation af kondrocytter [24] og vasodilatation [25].

På nuværende tidspunkt udfører professor Per Bech yderligere et studie med T-PEMF på depressive patienter, hvor effekten testes på to patientgrupper. En gruppe behandles en gang om dagen og testes overfor en gruppe der behandles to gange om dagen (en såkaldt dosis-respons-undersøgelse).

## T-PEMF til Parkinson patienter

Præliminære resultater med T-PEMF viser, at en positiv effekt kan forventes ved PD [26] I 2006 gennemførte overlæge Ole Gredal og overlæge Lene Wermuth et åbent pilotstudie på 8 patienter med PD. Formålet med pilotstudiet var at undersøge, om patienter behandlet med T-PEMF kunne opnå en bedre motorisk funktionsevne (målt ved UPDRS-skalaen), en forbedret livskvalitet (målt ved hjælp af et spørgeskema "PDQ-8") samt en reducering af depressionssymptomer (målt ved hjælp af spørgeskemaet "MDI") gennem behandling med T-PEMF.

Resultaterne i pilotstudiet viste at livskvaliteten blev signifikant forbedret ($p = 0{,}009$) og ligeledes blev depressionssymptomerne reduceret signifikant ($p = 0{,}011$). Endvidere kunne der konstateres en lille forbedring i den samlede score for den motoriske funktion (UPDRS). Resultaterne fra dette åbne pilotstudium, samt en statistisk analyse af data, førte til ønsket om at gennemføre en større klinisk kontrolleret og dobbeltblindet randomiseret undersøgelse på 120 patienter med PD som der nu søges godkendelse til.

Studiet vurderes at være klinisk relevant da behandlingen med T-PEMF til Parkinson-patienter mangler yderligere evidens, og vil såfremt behandlingen har signifikant effekt, have stor indflydelse på fremtidens behandling af Parkinson patienter.

## Forsøgets metode

Undersøgelsen med T-PEMF udføres som dobbeltblindt placebokontrolleret forsøg, hvor halvdelen af parkinson-patienterne randomiseres til aktiv T-PEMF behandling via en randomiseringsproces hvor randomization.com anvendes. Randomiseringsprocessen foretages af en person der ellers ikke

er involveret i forsøget. Randomiseringen stratificeres ud fra randomiseringskriterierne: alder, køn, parkinsonstadiet (Hoehn and Yahr Scale) for at minimere bias i studiet.

Endvidere vil en rask referencegruppe på op til 90 personer blive inkluderet for at kunne vurdere den relative størrelse af en eventuel effekt af aktiv og inaktiv behandling. Referencegruppen modtager ingen behandling.

De aktive og inaktive T-PEMF apparater vil fremtræde identiske, og under behandlingen vil en kontrollampe blinke. Placebobehandlingen foregår uden dannelse af et elektrisk felt. Deltagerne vil ikke under selve behandlingen kunne mærke eller høre om de modtager aktiv eller inaktiv behandling. Der vil i hvert apparat være et hukommelseskort der aflæser al aktivitet vedrørende anvendelse af apparatet, dette aflæses i et computerprogram hvorved det kan kontrolleres hvorvidt der er compliance i forhold til behandlingen.

Undersøgerne og patienterne er uvidende om hvilken behandling der gives. Patienterne må ikke omtale nogle forhold vedrørende apparatet eller behandlingen for undersøgerne. Overtrædelse af dette medfører eksklusion fra forsøget. Alle procedurer inklusiv information og instruktion i brug af apparaterne udføres af sundhedsfagliguddannet personale.

Statiske beregninger udført ud fra det ikke blindede pilotstudiets resultater, viste at 120 parkinson-patienter skal inkluderes i undersøgelsen, for at det kan forventes at vise en signifikant effekt på effektmålet livskvalitet (målt med PDQ-8). De 120 patienter fordeles ligeligt henholdsvis i en interventionsgruppe og en placebogruppe. For at opnå en virkning på de enzymatiske processer i hjernen, og dermed en klinisk effekt for patienten af behandlingen, forventes en behandlingsvarighed på mindst 4-5 uger med 1 daglig behandling, hvorfor 8 uger er valgt for at være sikker på at der opnås en maksimal effekt.

For at studiet skal kunne gennemføres praktisk under hensynstagen til eventuel variation af specielt motoriske symptomer i løbet af dagen, vil grupper af 6 patienter blive randomiseret og indgå i undersøgelsen med ca. 6 ugers mellemrum. Der er fornuværende 14 apparater til rådighed således at der vil være 2 apparater i reserve.

Patienterne skal søges at være optimalt medicinsk behandlet for deres Parkinsons sygdom før de indgår i studiet. Medicinen tilstræbes at blive holdt uændret under undersøgelsen. Skulle der vise sig tegn til overdosering i løbet af studiet grundet en gunstig virkning af T-PEMF reduceres dosis.

I forsøget anvendes 3 forskellige effektmål: Kliniske, neuropsykologiske og motoriske.

Behandlingseffekten ønskes evalueret ved at registrere og analysere de ændringer der sker på det motoriske og non-motoriske område fra baseline til undersøgelsens afslutning, samt efter en followup periode. I projektet anvendes 3 forskellige effektmål: Kliniske, neuropsykologiske og motoriske.

Til måling af parkinsonpatienternes kliniske symptomer vil "Unified Parkinson's Disease Rating Scale" (UPDRS) blive anvendt. Udførelsen af "Parkinson's Disease Questionnary 39" (PDQ-39) blive benyttet til kategorisering af parkinsonpatienternes helbredsrelaterede livskvalitet.

Neuropsykologiske effektmål: Depressionstendens vil blive vurderet ud fra Major (ICD-10) Depression Spørgeskemaet, HAM-D6, Apati skalaen og WHO-5. Kognitionen vurderes via MoCA og MMSE. Til overordnet måling af non-motor symptomer anvendes NMS. Til måling af kognitive og neurologiske processer anvendes Stroop. Prøven bygger på antagelsen om, at mennesker læser ord hurtigere end de kan identificere farvenavne. De kognitive funktioner, der måles i testen kan anvendes til at give information om deltagerens evne til at håndtere kognitivt stress og komplekse kognitive stimuli.

Motoriske funktionsmål: Hvile tremor, gangkapacitet (f.eks. 6 Minute walk test), modificeret Timed Up and Go, dynamisk og statisk balance.

Referencegruppen testes én gang og kun i de motoriske funktionsmål samt Stroop testen.

T-PEMF apparatet kan ligeledes dagligt opsamle subjektive patientoplysninger i forbindelse med de to daglige behandlinger, og disse registreringer kan sammenholdes med data fra besøgene på klinikkerne. Dette for at vurdere om behandlingen rent faktisk har været anvendt. Ligeledes registreres patienternes antal skridt pr. dag med skridttællere.

Alle forsøgsdata registreres i patientmapper med angivelse af forløb, eventuelle bivirkninger og komplikationer. Umiddelbart efter foretages indtastning i Databasen under Dansk Selskab for Bevægeforstyrrelser (DANMODIS) eller anden database. I forbindelse med projektet udtages en 5 ml veneblodprøve før og efter behandlingsinterventionen. Denne analyseres for BDNF som markør for nervevækst og for VEGF som markør for angiogenese. Blodprøver analyseres løbende og der oprettes ikke en biobank. Blodprøven tages to gange i studiet af den forsøgsansvarlige og analyseres straks og destrueres øjeblikkeligt herefter.

De motoriske funktionsmål testes på Institut for idræt og ernæring Integreret fysiologi Københavns Universitet. Blodprøvetagning foretages ligeledes på instituttet.

På de to centre beliggende på OUH og i Taastrup foretages måling af de kliniske og de neuropsykologiske effektmål.

**Statistiske overvejelser**
Resultaterne fra forsøget bearbejdes statistisk, interventionsgruppen og placebogruppens resultater sammenlignes med hinanden, og hvor relevant med referencegruppen. Statistikerfunktionen ved institut for regional Sundhedsforskning vil bidrage med vejledning. De statistiske beregninger vil fastslå validiteten af forsøgsresultaterne.
Statistiske beregninger udført ud fra det ikke blindede pilotstudiets resultater, viste at 120 parkinson -patienter skal inkluderes i undersøgelsen, for at det kan forventes at vise en signifikant effekt på effektmålet livskvalitet (målt med PDQ-8). De 120 patienter fordeles ligeligt henholdsvis i en interventionsgruppe og en placebogruppe. For at opnå en virkning på de enzymatiske processer i hjernen, og dermed en klinisk effekt for patienten af behandlingen, forventes en behandlingsvarighed på mindst 4-5 uger med 1 daglig behandling, hvorfor 8 uger er valgt for at være sikker på at der opnås en maksimal effekt. Det estimeres at der skal være 60 projektdeltagere pr. center. I forhold de præliminære resultater fra effekten af T-PEMF på depression kan et deltager antal interventionsgruppen være 40-50 og stadigvæk vil kunne producere et validt statistisk datasæt. Der er lavet styrkeberegning for primære effektmål UPDRS i dette kommende studie der viser at ved en styrke på 90% og et signifikans niveau på 5% vil 84 deltagere være statistisk signifikant. Det vil sige 42 deltagere i placebogruppen og interventionsgruppen. Derfor vurderes det at en interventionsgruppe på 60 er sufficient for at sikre validiteten af data og samtidigt tage højde for dropouts der estimeres til 10%.

Det skønnes ikke nødvendigt at foretage interim analyse, da der ud fra tidligere studier med behandlingsvarighed på 8 uger ikke er observeret bivirkninger. Da behandlingstiden kun er 8 uger skønnes det ikke etisk uforsvarligt at undlade at udføre interimanalyse.

Designet af studiet er et randomiseret klinisk studie med ét kontinuerligt primært effektmål.
Data vil blive anonymiseret og analyseret af en statistiker, som vil være blindet for hvilken behandling patienterne har modtaget dvs. aktiv eller placebo. Deskriptiv statistik for de to grupper vil blive genereret.

Ved intention to treat analyser anvendes LOCT og mixed model. Denne model sikrer at missing data ikke vil skabe bias, hvis missing data er tilfældigt fordelt. Således at der når studiet er afsluttet kan afgøres om "missing data" er tilfældigt fordelt.
Da Re5 behandling er en ny indfaldsvinkel til behandling af Parkinsons sygdom vil det være værdifuldt at evaluere en mulig effekt af Re5 behandling, hvis behandlingen tages som specificeret dvs. compliance ≥ 80%. Derfor vil per protokol analyser også blive udført.
For at reducere forekomsten af missing data vil primær investigator eller en kvalificeret stedfortræder sikre at alle spørgeskemaer udfyldes på de planlagte tidspunkter. Hvis projektdeltagerne ikke møder op til de aftalte testtidspunkter vil de blive kontaktet telefonisk af primær investigator eller en kvalificeret stedfortræder.
Data vil blive analyseret med SPSS og SAS. Succeskriterierne for afprøvningen er en signifikant forskel til den positive side hos den aktive gruppe sammenlignet med placebogruppen vedrørende primært og sekundære effektmål.

**Inklusion- og eksklusion for patientgruppen**

*Inklusionskriterier til forsøget er:*

- Patienter med Idiopatisk Parkinsons sygdom H & Y 1-3 defineret efter standardkriterier.
- MMSE > 22 (anvendes til at screene for begyndende demensudvikling).
- Alder > 18 år.
- Patienten er i stand til at forstå, acceptere og gennemføre de planlagte procedurer.

*Eksklusionskriterier til forsøget er:*

- Patienten har på inklusionstidspunktet tidligere været i T-PEMF behandling.
- Ændringer i den farmakologiske anti-parkinson behandling indenfor de sidste 6 uger.
- Udtalt demens eller anden hjerneskade, der kan influere på evnen til at afgive informeret samtykke, eller vanskeliggøre vurderingen af patientens tilstand.
- Psykotisk lidelse, eller andre psykopatologiske tilstande, der nødvendiggør anden intervention.
- Misbrug af alkohol eller stoffer.
- I behandling med Deep Brain stimulation.
- Graviditet eller amning.
- Epilepsi.
- Aktive implantater i form af pacemakere og andre som f.eks. cochlear implantater.
- Aktivt medicinsk udstyr f.eks. insulinpumper, baklofenpumper og andet.
- Deltagelse i andre forsøg eller afprøvninger i projektperioden.
- Aktuel eller tidligere kræftsygdom i hjernen, leukæmi, modermærkekræft, pladecellekræft, eller andre kræftformer i hoved/hals region.
- Autoimmun sygdom.
- Åbne sår i hovedbund.

Der stilles ingen krav til patienternes køn og alder så længe at de opfylder inklusionskriterier.

**Inklusions og eksklusionskriterier for referencegruppen**

*Inklusionskriterier for referencegruppen er:*

- 20-85 år
- Deltageren er i stand til at forstå, acceptere og gennemføre de planlagte procedure
- Føler sig sund og rask

*Eksklusionskriterier for referencegruppen er:*

- Kendte neurologiske eller neuromuskulære sygdomme
- Nylig operation, der har nedsat det aktuelle fysiske funktionsniveau

Den forsøgsansvarlige, dennes kvalificerede stedfortræder og styregruppen forbeholder sig ret til at afvise interesserede referencedeltagere selvom disse opfylder inklusionskriterierne, således at den endelige referencegruppe kommer til at matche pateintgruppen i henhold til fordeling af alder og køn.

**Bivirkninger, risici og ulemper**

Ved transkraniel magnetisk stimulation (TMS) givet i højere doser end dem, der anvendes i dette studie, har der ikke været observeret langtidsbivirkninger.

Behandling med elektromagnetiske felter herunder T-PEMF er stort set uden gener eller bivirkninger [19]. Dette bekræftes af tidligere undersøgelser der ikke har vist bivirkninger vurderet ved bivirkningsskalaen UKU (udvalg for kliniske undersøgelser). I de undersøgelser har antallet af behandlinger været mange og behandlingen har strakt sig over lang tid. Foreløbige rapporter om bivirkningsprofilen for T-PEMF behandling indikerer at behandlingerne kan give let og forbigående kvalme og hovedpine. Endvidere er der i nogle tilfælde rapporteret om ubehag i nakkemusklerne, grundet hjelmens vægt. Der er fornuværende ikke rapporteret om andre bivirkninger.

Da forsøgsdeltagerne kan modtage behandlingen derhjemme, og kan varetage rolige dagligdags funktioner, som for eksempel læsning og strikning, mens de modtager behandlingen og behandlingen har en varighed på 30 min 1 gang om dagen, skønnes det ikke at behandlingen bliver til gene for patienten. Det er også vigtigt at understrege, at deltagelse i forsøget ikke har indflydelse på de rettigheder patienten har til behandling for sin Parkinsons sygdom.

Patienterne skal komme til kontrol og testning to gange på institut for idræt og ernæring, integreret fysiologi Københavns Universitet for alle patienter og tre gange på testcentrene henholdsvis i Taastrup og Odense (OUH) alt afhængigt af geografisk inklusionsområde. Det skønnes ikke til væsentlig gene for forsøgsdeltagerne. Transportomkostninger vil blive dækket også for én pårørende. Det skønnes ikke at en veneblodprøvetagning 2 gange i løbet af forsøget vil være til gene for forsøgsdeltagerne da indgrebet er lille og forsøgsdeltageren kun tappes for 10 ml blod samlet under hele forsøget, samt at den forsøgsansvarlige har rutine i blodprøvetagning.

I undersøgelsens forløb registreres ved alle kontakter eventuelle hændelser med en angivelse af, om disse er alvorlige eller mindre alvorlige, samt en angivelse af om de ifølge undersøgerens skøn er relateret til T-PEMF behandlingen. Oplysningerne indsamles ved hjælp af UKU. I tilfælde af alvorlige uønskede hændelser, hvor T-PEMF behandlingen er medvirkende årsag til hændelsen, vil den lokale Videnskabsetiske komité, Sundhedsstyrelsen samt styregruppen omgående blive underrettet.

Endvidere vil Sundhedsstyrelse, den lokale Videnskabsetiske komité og styregruppen ligeledes blive orienteret om alle alvorlige hændelser og næsten-hændelser, som er indtruffet i perioden som helhed, samt give oplysning om forsøgspersonernes sikkerhed. Ved alle hændelser forstås både de forventede og uventede hændelser.

Følgende definitioner gælder for medicinsk udstyr:

En hændelse, der opfylder følgende tre kriterier er indberetningspligtig til Sundhedsstyrelsen:

**Adverse Event (AE):** enhver uønsket hændelse hos en forsøgsperson i en klinisk afprøvning ved/efter brug af et medicinsk udstyr, uden at der nødvendigvis er sammenhæng mellem denne brug og den uønskede hændelse

**Adverse Device Effect (ADE):** en uønsket hændelse relateret til brugen af det medicinske udstyr

**Serious Adverse Event (SAE/næsten hændelse):** en hændelse der medfører

- død
- livstruende skade eller sygdom
- varig skade på kroppen eller kropsfunktioner
- hospitalsindlæggelse eller forlængelse af hospitalsophold
- at medicinsk eller kirurgisk behandling er nødvendig for at undgå ovenstående
- fosterdød, en medfødt anomali eller misdannelse hos fosteret eller anden negativ påvirkning af fosteret

En næsten-hændelse er en udstyrsrelateret hændelse, som ikke har en alvorlig udgang, fordi der er grebet ind, inden hændelsen har udviklet sig.

**Herunder er også:** alvorlige hændelser og næsten-hændelser der skyldes unøjagtige eller mangelfulde resultater fra diagnostisk udstyr for eksempel:

- fejldiagnose
- forsinket diagnose
- forsinket behandling
- forkert behandling
- og hvor fejlen skyldes tekniske fejl eller mangler ved udstyret, brugsanvisningen, mærkningen, brugen eller vedligeholdelsen af udstyret

**Serious Adverse Device effect (SADE):** en alvorlig hændelse, som er relateret til brugen af det medicinske udstyr.

Studiet vil blive udført i henhold til ISO 14155 og monitoreret af GCP enheden ved Odense Universitetshospital.

Det skønnes ikke, at der er risici eller ulemper ved deltagelse i referencegruppen.

**Respekten for forsøgspersonernes fysiske og mentale integritet samt privatlivets fred**.
Oplysninger om forsøgsdeltagerne beskyttes efter lov om behandling af personoplysninger samt sundhedsloven. Projektet anmeldes til datatilsynet og persondataloven overholdes i forsøget.
Den forsøgsansvarlige ønsker at anvende oplysninger fra patientjournaler på de forsøgsdeltagere der deltager i forsøget. Det drejer sig om oplysninger vedrørende farmakologisk behandling, sygdomsstadie, almene helbredsoplysninger og speciale specifikke oplysninger her tænkes på Parkinsons sygdom. Oplysninger anvendes til at inkludere patienter i forsøget, samt for at kunne udarbejde statistisk valide data ved endpoint. Der vil kun blive læst de dele af journalen der omfatter ovenstående. Det skønnes at disse oplysninger er relevante og nødvendige for forskningsprojektet. Forsøgsdeltagere vil blive grundigt informeret om omfanget af journal gennemgangen og grundlaget for denne.
Der indhentes ingen journaloplysninger på deltagere i den raske referencegruppe.

**Økonomiske forhold.**
Den forsøgsansvarlige og styregruppen modtager ingen vederlag for gennemførelse af forsøget. Hverken den forsøgsansvarlige eller styregruppen har økonomisk interesse i det firma der leverer apparaterne. Endvidere har den forsøgsansvarlige og styregruppen ingen tilknytning til støttegiveren DEN A.P. MØLLERSKE STØTTEFOND.

Initiativet til forskningsprojektet er taget af en styregruppe bestående af:

Overlæge, klinisk lektor Lene Wermuth (Ph.d.-hovedvejleder)

Overlæge, dr. med Ole Gredal

Lektor, ph.d. Bente Rona Jensen (Ph.d.-vejleder)

Professor, dr.med. Per Bech (Ph.d.-vejleder)

Læge Bo Mohr Morberg (forsøgsansvarlig)

Der foreligger økonomisk bevilling til såvel gennemførelse af projektet samt Ph.d. forløb fra DEN A. P. MØLLERSKE STØTTEFOND med et beløb på kr. 4.962.000 se budget bilag 13.

Det kliniske studie ledes af Neurologisk forskningsenhed OUH. Bevillingen administreres af OUH og er under statsrevision.

Støtten udbetales som en fast sum og der oprettes to forskningskonti i OUH regi der vil være økonomisk administrator. Én konto der dækker lønomkostninger til den forsøgsansvarlige og én konto der dækker omkostninger til øvrig drift.

Et eventuelt overskydende støttebeløb vil blive tilbageført til fonden.

**Relevante klausuler i kontrakten mellem sponsor og forsøgsstedet**
Der er ingen klausuler mellem den A.P. Møllerske Støttefond og den forskningsvarlige og styregruppen vedrørende publicering. Den forsøgsansvarlige har fuld adgang til data og de fulde rettigheder til offentliggørelse af data, producenten af apparatet har ingen adgang til forsøgsdata.

**Vederlag eller andre ydelser til forsøgspersoner**.
Forsøgspersonerne i patientgruppen modtager transportgodtgørelse efter gældende takst vedrørende kørselsgodtgørelse ved kørsel i egen bil eller billigste offentlige transport. Dette gælder for de gange patienter fra Fyn og Sjælland skal komme til testcentrene på OUH og Taastrup til kontrol og tests. På turen til Taastrup dækkes desuden transportudgifter således at hver forsøgsdeltager kan få én pårørende med.
Deltagere i referencegruppen modtager ingen ydelser eller godtgørelser.

**Hvervning af deltagere.**
Projektet forventes at rekruttere patienter fra to geografiske områder dels fra Region Syddanmark dels Region Hovedstaden. Via kontakt til de respektive neurologiske afdelinger, praktiserende speciallæger i neurologi samt Dansk Parkinsonforenings lokalkredse vil parkinsonpatienter blive inviteret til informationsmøder med henblik på en senere inklusion i undersøgelsen. Der planlægges to inklusions centre dels på neurologisk afdeling OUH og Handicaporganisationernes Hus i Tåstrup.

Referencedeltagere søges hvervet gennem pårørende til forsøgsdeltagere i patientgruppen og gennem kontakt til foreninger i Taastrup og nærområde. Der hverves primært fra Sjælland for at mindske referencedeltagernes egne udgifter ved deltagelse. Andre interesserede, der ikke har deltagende pårørende, eller er medlem i de kontaktede foreninger, kan også deltage. Potentielle referencedeltagere og foreninger kontaktes via e-mail vedhæftet den skriftlige deltagerinformation. I deltagerinformationen er opgivet kontaktoplysninger til videnskabelige assistent Anne Sofie Bøg Malling, der kan kontaktes ved interesse for deltagelse.

**Tilgængeligheden af oplysninger for forsøgspersoner.**
Forsøgspersoner får udleveret kontaktoplysninger på relevant sundhedspersonale der kan assistere med at besvare de forespørgsler som forsøgsdeltagerne måtte have. Forsøgsdeltagerne vil have adgang til kontakt data på ansat projekt sygeplejerske samt forsøgsansvarlig. Den enkelte forsøgsdeltager vil i den aktive del af forsøget være tilknyttet en af ovenstående kontaktpersoner. Forsøgsdeltagerne vil, såfremt at de har givet samtykke til dette jævnfør anvendte samtykkeerklæring fra det videnskabsetiske komite system, blive informeret om resultaterne af forsøget skriftligt i let læseligt sprog, når forsøget er afsluttet og det videnskabelige arbejde er færdiggjort.

**Offentliggørelse af forsøgsresultater.**
Resultaterne fra projektet forventes at bidrage med 2-4 videnskabelige artikler, der kan publiceres internationalt peer-reviewed, samt ved møder såvel nationalt, som internationalt. Resultaterne søges offentliggjort uanset om resultaterne er positive, negative eller inkonklusive. Der vil løbende under forsøget, når resultaterne tillader det, blive udarbejdet præliminære artikler og endelige artikler efter

afslutningen af forsøget.

**Videnskabsetisk redegørelse.**

Studiet udføres i henhold til gældende dansk lovgivning med anmeldelse til Datatilsynet og Videnskabs Etisk Komité samt sundhedsstyrelsen. Opbevaring af data sker forsvarligt efter gældende regler. Det sikres at persondataloven overholdes.

Forsøgsprotokollen fra 2006 er tidligere godkendt af Videnskabsetisk Komité (journalnummer 01 278045) og af Datatilsynet, men der skal genansøges ligesom Sundhedsstyrelsen skal ansøges om tilladelse til brug af T-PEMF apparatet.

Såfremt en behandling mod Parkinsons sygdom skal være klinisk effektfuld og forsvarlig skal behandlingen leve op til at følgende kriterier opfyldes: behandlingen vil have en effekt på sygdommen herunder at behandlingen samlet set kan øge pateintens livskvalitet, acceptable bivirkninger ud fra patientsynspunkt og behandlersynspunkt.

T-PEMF har ved et tidligere forsøg med Parkinsons sygdom [26] demonstreret, der synes at være en effekt på livskvalitet og depression. Det er væsentligt at påvise om de resultater fundet ved det tidligere forsøg kan verificeres ved en større og randomiseret undersøgelse. Det er tillige væsentligt at få et indblik i, om patienter, som én gang har profiteret af T-PEMF kan gøre dette igen. Samt hvorvidt at parkinson-patienter via behandlingen kan få afhjulpet de motoriske funktioner og de non-motoriske symptomer af både behandlelig samt  behandlingsrefraktær karakter.

Undersøgelser indikerer at bivirkningsprofilen er positiv med relativt beskedne og forbigående bivirkninger. Indtil nu har T-PEMF behandlinger, således som også beskrevet i afsnittet om bivirkninger, kun resulteret i meget få og kortvarige bivirkninger. Imidlertid er det af afgørende betydning at undersøge, om de foreløbige rapporter om bivirkninger er i overensstemmelse med den faktiske forekomst af bivirkninger eller hændelser som følge af T-PEMF behandling.

Da studiet designet bygger på anvendelse af en interventionsgruppe og en placebogruppe er det meget relevant at undersøge hvorvidt anvendelse af placebo udgør et etisk dilemma. For at undersøge om en behandlingsmetode er virksom mod en sygdom er det nødvendigt at anvende placebobehandling. I om med at der ved T-PEMF behandling ikke hidtil er set alvorlige bivirkninger, og der er videnskabelig evidens for at T-PEMF har en fysiologisk effekt som også vurderes at have symptomlindrende effekt hos patienter med Parkinsons sygdom skønnes det at det er etisk forsvarligt at anvende en placebogruppe i studiet. Endvidere stilles de patienter der er med i forsøget ikke dårligere i deres rettigheder i forhold til specialiseret behandling mod Parkinsons sygdom. Således vil alle deltagere både i interventionsgruppe og placebogruppen modtage behandling som alle andre parkinson-patienter.

Det vurderes ikke at deltagelse i projektet vil have en betydning for hvorledes patienter kan udføre dagligdags gøremål da T-PEMF giver minimal negativ indflydelse i dagligdagen.

Alle forsøgsdeltagere bliver informeret om, at de ved tilfældig udvælgelse bliver allokeret enten til en placebo gruppe eller interventionsgruppe. Forsøgsdeltagerne har således mulighed for at fravælge deltagelse i studiet.

Såfremt resultaterne af denne undersøgelse viser at der er en effekt vurderet ud fra subjektive og objektive kriterier samtidig med at der er en acceptabel bivirkningsprofil, indebærer dette at et muligt nyt behandlingstiltag for patienter med Parkinsons sygdom.

**Oplysning om erstatnings- eller godtgørelsesordninger.**

Forsøgsdeltagerne er dækket af patientforsikringsordninger der dækker de skader der måtte blive påført forsøgspersoner i forbindelse med sundhedsvidenskabelige forskningsprojekter. Forsøget udføres under direkte ansvar fra OUH.

**Protokolresumé.**

**Titel**

T-PEMF (Transkranielle Pulserende ElektroMagnetiske Felter) ved Parkinsons sygdom.

**Forsøgsansvarlig**

Bo Mohr Morberg Neurologisk afdeling OUH.

**Forsøgets formål**

Formålet er at undersøge, om 8 ugers behandling med elektromagnetiske felter der gives i hjernen via en hjelm kan mindske sværhedsgraden af symptomer patienter med Parkinsons sygdom oplever. Her tænkes der på forstyrrelser at patientens bevægelser og psykiske påvirkninger samt problemer med tale og vandladning.

Endvidere er formålet at afklare hvorvidt patienter med Parkinsons sygdom oplever en statistisk bedring af symptomerne ved behandling med aktiv T-PEMF, når de behandles dagligt i 8 uger i forhold til en gruppe der modtager inaktiv T-PEMF .

**Forsøgets metode**

Undersøgelsen med T-PEMF udføres som dobbeltblindet placebokontrolleret, det vil sige at hverken patient eller undersøger er vidende om hvem der modtager den aktive behandling eller den inaktive behandling. Halvdelen af de 120 parkinson-patienter udvælges tilfældigt til aktiv T-PEMF behandling via et randomiseringsprogram resten vil modtage inaktiv T-PEMF behandling. Randomiseringen foregår ud fra randomiseringskriterierne: alder, køn, parkinsonstadiet (Hoehn and Yahr Scale) for at minimere fejlkilder i studiet. De aktive og inaktive T-PEMF apparater vil fremtræde identiske, og under behandlingen vil en kontrollampe blinke. Den inaktive behandling foregår uden dannelse af et magnetfelt. Deltagerne vil ikke under selve behandlingen kunne mærke om de modtager aktiv eller inaktiv behandling. Patienterne må ikke omtale nogle forhold vedrørende apparatet eller behandlingen for undersøgerne. Overtrædelse af dette medfører udelukkelse fra forsøget. Alle procedurer inklusiv information og instruktion i brug af apparaterne udføres af sundhedsfagliguddannet personale.

De 120 patienter fordeles ligeligt henholdsvis i en behandlingsgruppe der modtager aktiv T-PEMF og en placebogruppe der modtager inaktiv T-PEMF behandling. Varigheden af behandlingen er 8 uger da nervevævet i hjernen tager tid om at forbedre sig. Grupper af 6 patienter vil indgå i forsøget med ca. 6 ugers mellemrum.

Der inddrages yderligere en rask referencegruppe, der ikke modtager behandling.

I forbindelse med projektet udtages en 5 ml veneblodprøve før og efter behandlingsinterventionen. Denne analyseres for BDNF som markør for nervevækst og for VEGF som markør for angiogenese. Blodprøver analyseres løbende og der oprettes ikke en biobank. Blodprøven tages to gange i studiet af den forsøgsansvarlige og analyseres straks og destrueres øjeblikkeligt herefter.

Patienterne skal være optimalt medicinsk behandlet før de indgår i studiet. Medicinen tilstræbes at blive holdt uændret under undersøgelsen.

**Statistiske overvejelser**

Resultaterne fra forsøget bearbejdes statistisk, interventionsgruppen og placebogruppens resultater sammenlignes med hinanden, og hvor relevant med referencegruppen. Statistikerfunktionen ved institut for regional Sundhedsforskning vil bidrage med vejledning. De statistiske beregninger vil fastslå validiteten af forsøgsresultaterne.

Statiske beregninger udført ud fra det ikke blindede pilotstudiets resultater, viste at 120 parkinson -patienter skal inkluderes i undersøgelsen, for at det kan forventes at vise en signifikant effekt på effektmålet livskvalitet (målt med PDQ-8). De 120 patienter fordeles ligeligt henholdsvis i en interventionsgruppe og en placebogruppe. For at opnå en virkning på de enzymatiske processer i hjernen, og dermed en klinisk effekt for patienten af behandlingen, forventes en behandlingsvarighed på mindst 4-5 uger med 1 daglig behandling, hvorfor 8 uger er valgt for at være sikker på at der opnås en maksimal effekt. Det estimeres at der skal være 60 projektdeltagere pr. center. I forhold de præliminære resultater fra effekten af T-PEMF på depression kan et deltager antal interventionsgruppen være 40-50 og stadigvæk vil kunne producere et validt statistisk datasæt. Der er lavet styrkeberegning for UPDRS der viser at ved en styrke på 90% og et signifikans niveau på 5% vil 84 deltagere være statistisk signifikant. Det vil sige 42 deltagere i placebogruppen og interventionsgruppen. Derfor vurderes det at en interventionsgruppe på 60 er sufficient for at sikre validiteten af data og samtidigt tage højde for dropouts der estimeres til 10%.

**Forsøgspersoner, herunder inklusions- og eksklusionskriterier**

Krav til forsøgsdeltagerne i patientgruppen i forhold til deltagelse i forsøgsprojektet

*Følgende skal opfyldes for at komme med i forsøget:*

- Patienter med Parkinsons sygdom i mildt til moderat stadie
- Test for demens hvor der skal opnås en score på over 22
- Alder > 18 år
- Patienten er i stand til at forstå, acceptere og gennemføre de planlagte procedurer.

*Hvis én af følgende emner opfyldes kan man ikke deltage i forsøget*

- Patienten har på tidspunktet for rekruttering tidligere været i T-PEMF behandling.
- Der må ikke være ændringer af den medicin mod parkinson patienten modtager indenfor de sidste 6 uger.
- Udtalt demens eller anden hjerneskade, der kan påvirke evnen til at afgive informeret samtykke, eller vanskeliggøre vurderingen af patientens tilstand.

- Sindslidelse der nødvendiggør anden intervention.
- Misbrug af alkohol eller stoffer.
- Pacemaker.
- I behandling med dyb hjernestimulering.
- Graviditet/amning
- Epilepsi
- Aktive implantater i form af pacemakere og andre som f.eks. implantater i ørerne.
- Aktivt medicinsk udstyr f.eks. medicinpumper og andet.
- Deltagelse i andre forsøg eller afprøvninger i projektperioden.
- Aktuel eller tidligere kræftsygdom i hjernen, leukæmi, modermærkekræft, hudkræft, eller andre kræftformer i hoved/hals region.
- Autoimmun sygdom.
- Åbne sår i hovedbund.

Der stilles ingen krav til patienternes køn og alder så længe at de opfylder kriterierne for at deltage.

Krav til forsøgsdeltagerne i referencegruppen i forhold til deltagelse

*Følgende skal opfyldes for at kunne deltage:*

- 20-85 år
- Deltageren er i stand til at forstå, acceptere og gennemføre de planlagte procedure
- Føler sig sund og rask

*Hvis ét af følgende kriterier er opfyldt, kan man ikke deltage i forsøget:*

- Kendte neurologiske eller neuromuskulære sygdomme
- Nylig operation, der har nedsat det aktuelle fysiske funktionsniveau

Den forsøgsansvarlige, dennes kvalificerede stedfortræder og styregruppen forbeholde sig ret til at afvise interesserede referencedeltagere selvom disse opfylder inklusionskriterierne, således at den endelige referencegruppe kommer til at matche patientgruppen i henhold til fordeling af alder og køn.

**Bivirkninger, risici og ulemper**

Behandling med elektromagnetiske felter herunder T-PEMF er stort set uden gener eller bivirkninger. Dette bekræftes af tidligere undersøgelser der ikke har vist bivirkninger vurderet ved bivirkningsskalaen UKU (udvalg for kliniske undersøgelser). I undersøgelserne har antallet af behandlinger været mange og behandlingen har strakt sig over lang tid. Foreløbige rapporter om

bivirkningsprofilen for T-PEMF behandling indikerer at behandlingerne kan give let og forbigående kvalme og hovedpine. I nogle tilfælde et ubehag i nakkemusklerne, grundet hjelmens vægt. Der er fornuværende ikke rapporteret om andre bivirkninger.

Da patienten kan modtage behandlingen derhjemme og kan varetage dagligdags funktioner mens de modtager behandlingen, samt at behandlingen har en varighed på 30 min 1 gang om dagen skønnes det ikke at behandlingen bliver til gene for patienten. Det er også vigtigt at understrege, at deltagelse i forsøget ikke har indflydelse på de rettigheder patienten har til behandling for sin Parkinsons sygdom. Det skønnes ikke at en veneblodprøve hvor der samlet udtages 10 ml. blod, foretaget af den forsøgsansvarlige med rutine i dette, vil være til væsentlig gene for forsøgsdeltageren.

Ved andre behandlinger med elektromagnetiske bølger af større styrke end dem der anvendes ved PEMF, har der ikke været observeret langtidsbivirkninger.

I undersøgelsens forløb registreres ved alle kontakter eventuelle hændelser med en angivelse af, om disse er alvorlige eller mindre alvorlige, samt en angivelse af om de ifølge undersøgerens skøn er relateret til T-PEMF behandlingen. I tilfælde af alvorlige uønskede hændelser, hvor T-PEMF behandlingen er medvirkende årsag til hændelsen, vil den lokale Videnskabsetiske komité omgående blive underrettet.

Endvidere vil Sundhedsstyrelse, den lokale Videnskabsetiske komité og styregruppen ligeledes blive orienteret om alle alvorlige hændelser og næsten-hændelser, som er indtruffet i perioden som helhed, samt give oplysning om forsøgspersonernes sikkerhed. Ved alle hændelser forstås både de forventede og uventede hændelser.

Det skønnes ikke, at der er risici eller ulemper ved deltagelse i referencegruppen.

**Økonomiske forhold**

Den forsøgsansvarlige og styregruppen modtager ingen vederlag for gennemførelse af forsøget. Hverken den forsøgsansvarlige eller styregruppen har økonomisk interesse i det firma der leverer apparaterne. Endvidere har den forsøgsansvarlige og styregruppen ingen tilknytning til støttegiveren DEN A.P. MØLLERSKE STØTTEFOND.

Initiativet til forskningsprojektet er taget af en styregruppe bestående af:

| | |
|---|---|
| Styregruppen: | Overlæge, klinisk lektor Lene Wermuth (Ph.d.-vejleder) |
| | Overlæge, dr. med Ole Gredal |
| | Lektor, ph.d. Bente Rona Jensen (Ph.d.-vejleder) |
| | Professor, dr.med. Per Bech (Ph.d.-vejleder) |
| | Læge Bo Mohr Morberg (forsøgsansvarlig) |

Der foreligger økonomisk bevilling til såvel gennemførelse af projektet samt Ph.d. forløb fra Den A. P. Møllerske Støttefond.

Det kliniske studie ledes af Neurologisk forskningsenhed OUH.

Støtten udbetales som en fast sum og der oprettes to forskningskonti i OUH regi der vil være økonomisk administrator. Én konto der dækker lønomkostninger til den forsøgsansvarlige og én konto der dækker omkostninger til øvrig drift.

Et eventuelt overskydende støttebeløb vil blive tilbageført til fonden.

**Offentliggørelse af forsøgsresultater**

Resultaterne fra projektet forventes at bidrage med 2-4 videnskabelige artikler, der kan publiceres internationalt, samt ved møder såvel nationalt, som internationalt. Resultaterne søges offentliggjort uanset om resultaterne er positive, negative eller inkonklusive. Der vil løbende under forsøget når resultaterne tillader det blive udarbejdet data til fremlæggelse i offentlig regi og endelige artikler efter afslutningen af forsøget.

**Videnskabsetisk redegørelse.**

Studiet udføres i henhold til gældende dansk lovgivning med anmeldelse til Datatilsynet og Videnskabs Etisk Komité samt sundhedsstyrelsen. Opbevaring af data sker forsvarligt efter gældende regler. Det sikres at persondataloven overholdes.

Forsøgsprotokollen fra 2006 er tidligere godkendt af Videnskabsetisk Komité (journalnummer 01 278045) og af Datatilsynet, men der skal genansøges ligesom Sundhedsstyrelsen skal ansøges om tilladelse til brug af PEMF apparatet.

Såfremt en behandling mod Parkinsons sygdom skal være klinisk effektfuld og forsvarlig skal behandlingen leve op til at følgende kriterier opfyldes: behandlingen vil have en effekt på sygdommen herunder at behandlingen samlet set kan øge pateintens livskvalitet, acceptable bivirkninger ud fra patientsynspunkt og behandlersynspunkt.

T-PEMF har ved et tidligere forsøg med Parkinsons sygdom [18] demonstreret, der synes at være en effekt på livskvalitet og depression. Det er væsentligt at påvise, at dette fund kan genfindes ved en ny undersøgelse. Det er tillige væsentligt at få et indblik i, om patienter, som én gang har profiteret af T-PEMF kan gøre dette igen. Samt hvorvidt at parkinson-patienter via behandlingen kan få afhjulpet de motoriske og non-motoriske symptomer af både behandlelig samt behandlingsrefraktær karakter.

Undersøgelser indikerer at bivirkningsprofilen er positiv med relativt beskedne og forbigående bivirkninger. Indtil nu har T-PEMF behandlinger, således som også beskrevet i afsnittet om bivirkninger, kun resulteret i meget få og kortvarige bivirkninger. Imidlertid er det af afgørende betydning at undersøge, om de foreløbige rapporter om bivirkninger er i overensstemmelse med den faktiske forekomst af bivirkninger eller hændelser som følge af T-PEMF behandling.

Da studiet designet bygger på anvendelse an en interventionsgruppe og en placebogruppe er det meget relevant at undersøge hvorvidt anvendelse af placebo udgør et etisk dilemma. For at undersøge om en behandlingsmetode er virksom mod en sygdom er det nødvendigt at anvende placebobehandling. I om med at der ved T-PEMF behandling ikke hidtil er set alvorlige bivirkninger, og der er videnskabelig evidens for at T-PEMF har en fysiologisk effekt som også vurderes at have symptomlindrende effekt hos patienter med Parkinsons sygdom skønnes det at det er etisk forsvarligt at anvende en placebogruppe i studiet. Endvidere stilles de patienter der er med i forsøget ikke dårligere i deres rettigheder i forhold til specialiseret parkinson behandling som helhed. Således vil

alle deltagere både i interventionsgruppe og placebogruppen modtage behandling som alle andre parkinson-patienter.

Det vurderes ikke at deltagelse i projektet vil have en betydning for hvorledes patienter kan udføre dagligdags gøremål da T-PEMF giver minimal negativ indflydelse i dagligdagen.

Alle forsøgsdeltagere bliver informeret om, at de ved tilfældig udvælgelse bliver allokeret enten til en placebo gruppe eller interventionsgruppe. Forsøgsdeltagerne har således mulighed for at fravælge deltagelse i studiet.

Såfremt resultaterne af denne undersøgelse viser at der er en effekt vurderet ud fra subjektive og objektive kriterier samtidig med at der er en acceptabel bivirkningsprofil, indebærer dette at et muligt nyt behandlingstiltag for patienter med Parkinsons sygdom.

**Hvervning af deltagere**

Projektet forventes at rekruttere patienter fra to geografiske områder dels fra Region Syddanmark dels Region Hovedstaden. Via kontakt til de respektive neurologiske afdelinger, praktiserende speciallæger i neurologi samt Dansk Parkinsonforenings lokalkredse vil parkinsonpatienter blive inviteret til informationsmøder mhp en senere inklusion i undersøgelsen. Der planlægges 2 inklusions centre dels på neurologisk afd. OUH og Handicaporganisationernes Hus Høje Tåstrup.

Referencedeltagere søges hvervet gennem pårørende til forsøgsdeltagere i patientgruppen og gennem kontakt til foreninger i Taastrup og nærområde. Der hverves primært fra Sjælland for at mindske referencedeltagernes egne udgifter ved deltagelse. Andre interesserede, der ikke har deltagende pårørende, eller er medlem i de kontaktede foreninger, kan også deltage. Potentielle referencedeltagere og foreninger kontaktes via e-mail vedhæftet den skriftlige deltagerinformation. I deltagerinformationen er opgivet kontaktoplysninger til videnskabelige assistent Anne Sofie Bøg Malling, der kan kontaktes ved interesse for deltagelse.

**Mundtlig deltagerinformation**

Den mundtlige information gives af den forsøgsansvarlige. Kontakten til mulige forsøgsdeltagere i patientgruppen er formidlet via brev indeholdt den skriftlige deltagerinformation, hvor de inviteres til et fælles mundtligt informationsmøde. Forsøgsdeltagerne har mulighed for at tage en pårørende med til informationsmødet som bisidder. De deltagere der accepterer at være med i forsøget vil modtage individuel mundtlig information inden de underskriver informeret samtykke erklæringen.
Deltagere i referencegruppen informeres individuelt over telefon af forsøgsansvarlige eller dennes stedfortræder og tilsendes den skriftlige deltagerinformation for referencegruppen, hvis ikke denne er erhvervet gennem rekrutteringsbrevet. Den mundtlige information vil blive repeteret før underskrivelse af informeret samtykke erklæring på undersøgelsesdagen.

**Følgende vil være indholdet i invitationen til patientgruppen (udover indholdet af skriftlig deltagerinformation)**

- Dato og tid for informationsmødet
- Der informeres om at indholdet er vedrørende en forespørgsel om deltagelse i et sundhedsvidenskabeligt forskningsprojekt.
- Der informeres om at det er muligt for patienten at tage en bisidder med til mødet.

Der gøres opmærksomt på at der er efter informationsmødet vil være en betænkningstid på 2 dage til at tage stilling til om hvorvidt man ønsker at være med i projektet, samt at der ved accept på at være forsøgsdeltager vil være et individuelt informationsmøde med den forsøgsansvarlige hvor endelig accept gives fra forsøgsdeltageren. Således at der indhentes samtykke efter skriftlig og individuel mundtlig information.

Selve informationsmødet afholdes en et lokale på afdeling N OUH og i et lokale i Handicaporganisationernes Hus i Taastrup. Mødet afholdes sidst på eftermiddagen således at der er færrest mulige personer på afdelingen og i Handicaporganisationernes Hus derved sikres at forstyrrelser minimeres. Mødetidspunktet tilrettelægges ikke senere end deltagerne skønnes at være kognitivt friske. Der vil være forfriskninger til deltagerne på informationsmødet.

Efter det mundtlige informationsmøde kan patienterne tage hjem og tale med pårørende om hvorvidt patienten ønsker at deltage i forsøget. Patienten får samtykkeerklæringen fra Videnskabsetisk Komité udleveret til det mundtlige informationsmøde, og kan således tage det med hjem i betænkningsfasen efter informationsmødet. Der forventes at der vil være 2 dages betænkningstid. Forsøgsdeltagerne får udleveret kontakt data på den forsøgsansvarlige således at deltagerne kan kontakte denne ved tvivlsspørgsmål.

Deltagere vil få tilsendt den skriftlige deltagerinformation på skrift senest 14 dage ingen mødet således at der er god tid til at få læst materialet igennem således at mulige spørgsmål kan tages med til mødet. Patienterne skal melde tilbage senest 7 dage før mødet om de ønsker at deltage således at forplejning og det rette lokale størrelse kan bookes. Der vil efter mødet være mulighed for at tale med den forsøgsansvarlige under mere uformelle rammer såfremt at nogle patienter skulle ønske dette.

Informationen leveres via let forståelige og let læselige PowerPoint slides som også udleveres til deltagerne i starten af mødet. Således vil lix tallet være tilpasset patientgruppen samt vil der ikke være lægefagtermer, disse vil være oversat til almindelig dansk således tilpasset til denne patientgruppes formodede demografi.

Der vil blive informeret om mulige bivirkninger, muligheden for uforudsete hændelser, mulige ulemper ved deltagelse i forsøget, og at der stilles krav til deltagerne i forsøget i forhold til compliance.

Deltagerne i forsøget vil få gennemlæst deres journal i forhold til aktuel behandling samt andre sygdomme. Deltagerne vil få at vide, at de deltager i et videnskabeligt forsøg der kan afgøre om der i fremtiden vil være en ny behandlingsmetode til dem.

Ved informeret samtykke anvendes skema fra Videnskabsetisk Komité.

Endvidere vil der i henhold til loven om medicinsk udstyr i tilfælde af inspektion gives fuldmagt til Sundhedsstyrelsen med henblik på at få adgang til patientens journal.

Findes der i løbet af forsøget informationer om effekten af behandlinger, bivirkninger fra behandlingen eller komplikationer vil forsøgsdeltagerne blive informeret. Endvidere vil forsøgsdeltagerne blive informeret hvis studiets design ændres væsentligt hvis det skønnes relevant for forsøgedeltagernes sikkerhed.

Forsøgsdeltageren vil såfremt denne ønsker det, modtage informationen om sin helbredstilstand såfremt der under forsøget fremkommer væsentlige oplysninger om denne.

Forsøgsdeltageren, vil såfremt denne ønsker dette, efter afslutning af forsøget blive informeret om resultaterne af studiet og de mulige konsekvenser dette kunne afføre for deltageren.

Såfremt at forsøges afbrydes før tid vil forsøgedeltageren blive informeret om årsagen hertil.

Væsentlige helbredsoplysninger vil blive meddelt til forsøgsdeltagerne.

Litteraturliste

[1]. Parkinsons sygdom. Klinisk vejledning 2 udgave. Danmodis; 2011

[2]. Lees AJ, Hardy J, Revisz T. Parkinson's disease. Lancet 2009;373:2055-6

[3]. Bretlau L, Lunde MA, Lindberg L, Unden M, Bech P. Repetitive Transcranial Magnetic Stimulation (rTMS) in combination with escitalopram in patients with treatment-resistant major depression. A double-blind, randomised, sham-controlled trial. Pharmacopsychiatry. 2008;41:41-47

[4]. Martiny K et al. Pulsed electromagnetic fields (PEMF) in treatment of resistant depression. A pilot study. International Society of Affective Disorders (Abstract). Second Biennial International Conference. Cancun, Mexico March 5th - 10th, 2004

[5]. Centonze D, Koch G, Versace V, Mori F, Rossi S, Brusa L, et al. Repetitive transcranial magnetic stimulation of the motor cortex ameliorates spasticity in multiple sclerosis. Neurology 68, 1045-1050, 2007

[6]. Krause P, Edrich T, Straube A. Lumbar repetitive magnetic stimulation reduces spastic tone increase of the lower limbs. Spinal cord 42, 67-72. 2004

[7]. Nielsen J.F, Sinkjaer T, Jakobsen J. Treatment of spasticity with repetitive magnetic stimulation; a double-blind placebo-controlled study. Mult Scler 2, 227-32,1996

[8]. Sherafat MA, Heibatollahi M, Mongabadi S, Moradi F, Javan M, Ahmadiani A. Electromagnetic Field Stimulation Potentiates Endogenous Myelin Repair by Recruiting Subventricular Neural Stem Cells in an Experimental Model of White Matter Demyelination. J Mol Neurosci. 2012 Sep;48(1):144-53

[9] Borsalino G, Bagnacani M, Bettati E et al. Electrical stimulation of human femoral intertrochanteric osteotomies. Double-blind study. Clin Orthop Relat Res 1988; (237):256- 63

[10] Sandyk R. Speech impairment in Parkinson's disease is improved by transcranial application of electromagnetic fields. Int J Neurosci. 1997 Nov;92(1-2):63-72

[11] Sandyk R. Treatment with electromagnetic fields improves dual-task performance (talking while walking) in multiple sclerosis. Int J Neurosci. 1997 Nov;92(1-2):95-102

[12]. Arias-Carrión O. Basic mechanisms of rTMS: Implications in Parkinson's disease. Int Arch Med. 2008 Apr 15;1(1):2

[13]. Randhawa BK, Farley BG, Boyd LA. Repetitive transcranial magnetic stimulation improves handwriting in Parkinson's disease. Parkinsons Dis. 2013;2013:751925

[14]. Martiny K, Lunde M, Bech P. Transcranial low voltage pulsed electromagnetic fields in patients with treatment-resistant depression. Biol Psychiatry. 2010 Jul 15;68(2):163-9. doi: 10.1016/j.biopsych.2010.02.017. Epub 2010 Apr 10

[15]. Bech P, Gefke M, Lunde M, Lauritzen L, Martiny K. The pharmacopsychometric triangle to illustrate the effectiveness of T-PEMF concomitant with antidepressants in treatment resistant patients: A double-blind, randomised, sham-controlled trial revisited with focus on the patient-reported outcomes. Depression Research and Treatment. Volume 2011, Article ID 806298, 6 pages

[16]. Andreasson K, Liest V, Lunde M, Martiny K, Unden M, Dissing S et al. Identifying patients with therapy-resistant depression by using factor analysis. Pharmacopsychiatry 2010;43:252-256

[17]. Santos-García D et al. Impact of non-motor symptoms on health-related and perceived quality of life in Parkinson's disease. J Neurol Sci. 2013 Jul 25. pii: S0022-510X(13)00316-X

[18]. Ulrik L Rahbek, Katerina Tritsaris, Steen Dissing. Interaction of Low-frequency, Pulsed Electromagnetic Fields with Living Tissue: Biochemical Responses and Clinical Results. Vol 2. No 1. 2005. Oral Biosciences & Medicine. 1-12

[19]. Oren M. Tepper, Matthew J. Callaghan, Edward I. Chang, Robert D. Galiano, Kirit A. Bhatt, Samuel Baharestani, et al. Electromagnetic fields increase in vitro and in vivo angiogenesis through endothelial release of FGF-2. *The FASEB Journal* express article10.1096/fj.03-0847fje. Published online June 18, 2004

[20] Macias MY, Battocletti JH, Sutton CH, Pintar FA, Maiman DJ. Directed and enhanced neurite growth with pulsed magnetic field stimulation. Bioelectromagnetics, 21 (2000), pp. 272–286

[21] Longo FM, Yang T, Hamilton S, Hyde JF, Walker J, Jennes L et al. Electromagnetic fields influence NGF activity and levels following sciatic nerve transection. J Neurosci Res 1999, 55:230-237

[22] Macias MY, Battocletti JH, Sutton CH, Pintar FA, Maiman DJ. Directed and enhanced neurite growth with pulsed magnetic field stimulation. Bioelectromagnetics 2000, 21:272-286

[23] Sisken BF, Kanje M, Lundborg G, Herbst E, Kurtz W. Stimulation of rat sciatic nerve regeneration with pulsed electromagnetic fields. Brain Res 1989. 485:309-316

[24] Pezetti F, De MM, Caruso A, Cadossi R, Zucchini P, Carinci F. Effect of pulsed electromagnetic fields om human chodrocytes: an in vitro study. Calcif Tissue Int 1999, 65:396-401

[25] Smith TL, Wong-Gibbons D, Maultsby J. Microcirculatory effects of pulsed electromagnetic fields. J Orthop Res. 2004 Jan;22(1):80-4.

[26]. Dethlefsen G, Gredal O, Wermuth L. Effect of pulsed electro magnetic fields in patients with idiopathic Parkinsons disease: A pilot study. Fremlagt som poster ved ”8th International Conference on Alzheimer's and Parkinson's Diseases AD/PD. March 14-18, 2007, Salzburg, Austria

[27]. Martiny K, Lunde M, Bech Transcranial low voltage pulsed electromagnetic fields in patients with treatment-resistant depression. Psychiatry. 2010 Jul 15;68(2):163-9. doi: 10.1016/j.biopsych.2010.02.017. Epub 2010 Apr 10.

Skriftlig deltagerinformation til patientgruppe:

Odense d. xxx

Kære fulde navn

Jeg tillader mig hermed at kontakte dig vedrørende deltagelse i et forsøgsprojekt omhandlende Parkinsons sygdom og behandling med elektrisk feltterapi forkortet T-PEMF.

Der anmodes om lov til at spørge om du vil deltage i et videnskabeligt forsøgsprojekt vedrørende elektrisk feltterapi behandling til patienter med Parkinsons sygdom.

For alle interesserede vil der blive afholdt et fælles informationsmøde. Såfremt man vælger at deltage i forsøget vil der inden at du afgiver endelig accept blive afholdt et individuelt informationsmøde for dig og din pårørende med den forsøgsansvarlige.

For praktiske spørgsmål vedrørende det mundtlige informationsmøde og spørgsmål til forsøget se venligst sidste side i dette brev.

Information om forsøget kan læses herunder

Titel: Behandling af hjernen med elektromagnetiske felter til patienter med Parkinsons sygdom.

**Transkranielle Pulserende ElektroMagnetiske Felter (T-PEMF) ved Parkinsons sygdom (PD).**

Forskning på dette område er meget vigtig, da det undersøges om elektromagnetisk behandling kan opretholde og om muligt forøge mængden af dopamin i hjernen. Resultaterne fra studiet vil kunne være med til at forbedre de symptomer patienter med Parkinsons sygdom har i både bevægeapparatet og af psykisk karakter; vandladningsgener og søvnbesvær. Forskning på området tegner et billede af, at elektromagnetiske felter kan medvirke til, at der går længere tid før man får symptomer på Parkinsons sygdom samt at hjernen kan ændre sig positivt ved behandling med elektromagnetiske felter. Dette forsøg vil bidrage til denne forskning og til fremtidig behandling patienter med Parkinsons sygdom. Du kan være medvirkende til at der forskes yderligere på området, og at det muligvis vil give adgang for en ny type behandling til patienter med Parkinsons sygdom.

Som deltager i forsøget vil du blive udvalgt tilfældigt til enten at modtage aktiv behandling eller inaktiv behandling. De forsøgsdeltagere der kommer i aktiv gruppen vil kunne drage nytte af at være med i forsøget, da det skønnes at behandlingen vil have positiv effekt på parkinson symptomer.

Behandling med elektromagnetiske felter herunder T-PEMF er stort set uden gener eller bivirkninger. Dette bekræftes af tidligere undersøgelser der ikke har vist bivirkninger. I undersøgelserne har antallet af behandlinger været mange og behandlingen har strakt sig over lang tid. Ved andre behandlinger med elektromagnetiske bølger af større styrke end dem der anvendes ved T-PEMF, har der ikke været observeret langtidsbivirkninger. Foreløbige rapporter om bivirkninger for T-PEMF behandling tyder på at behandlingerne kan give let og forbigående kvalme og hovedpine og i nogle tilfælde et ubehag i nakkemusklerne, grundet hjelmens vægt. Der er fornuværende ikke kendskab til andre bivirkninger.

Behandlingen foregår hjemme hos dig selv og forløber i 8 uger. Under behandlingen vil du kunne varetage dagligdags funktioner af rolig karakter for eksempel læse en bog, se fjernsyn, løse kryds og tværs eller strikke. Behandlingen har en varighed på 30 min én gang om dagen. Selve apparatet er udformet som en hjelm, se vedlagte foto, der bæres på hovedet de 30 min dagligt som behandlingen varer. Hjelmen skal opbevares sådan at den ikke udsættes for slag, for eksempel i et skab, når den ikke anvendes. Du vil blive grundigt vejledt i anvendelsen af apparatet ved udlevering af dette. Der vil endvidere blive taget telefonisk kontakt ugentligt til dig for at sikre at apparatet kan anvendes. Du vil få udleveret kontakt data på den forsøgsansvarlige således at du frit kan kontakte denne ved problemer under behandlingen. Det skønnes at behandlingen ikke vil blive til gene for dig. Det er vigtigt at understrege at deltagelse i forsøget ikke har indflydelse på de rettigheder du har til behandling for din Parkinsons sygdom. Der vil i forbindelse med udlevering af apparatur og test skulle påregnes transport til Købehavn to gange i løbet af forsøget samt tre gange til OUH når du skal testes. Transportudgifter svarende til billigste offentlige transport eller som kørselsgodtgørelse ved transport i privat bil dækkes af forsøgsmidler. Der dækkes også økonomisk for transport af én pårørende. Der vil blive taget 2 blodprøver i løbet af forsøget for at se hvorledes behandlingen påvirker hjerne og blodkar. Mængden af blod der udtages er på samlet 10 ml hvilket i mængde svarer til at få taget en blodprøve hos egen læge. Blodprøverne tages af den forsøgsansvarlige der har rutine i blodprøvetagning, og vil blive destrueret efter de er analyseret.

Forsøget har muligheden for at give en nytteværdi til fremtidens Parkinson patienter. Hvis forsøget viser en betydelig effekt af elektromagnetisk feltterapi vil dette kunne være med til at danne grundlag for den fremtidige behandling af Parkinson patienter.

Du kan udelukkes fra behandlingen såfremt du ikke har været ærlig omkring de kriterier der skal opfyldes ved indlemmelse i forsøget. Du kan ligeledes udelukkes fra forsøget, såfremt at du skulle blive så mentalt svækket, at du ikke kan tage ansvar for egen behandling. Ligeledes vil du blive udelukket såfremt apparatet misvedligeholdes eller udlånes til anden part. Hvis du skulle blive alvorligt syg i forsøgsperioden kan du blive udelukket fra forsøget. Som deltager i forsøget må du ikke omtale nogle forhold vedrørende apparatet eller behandlingen for undersøgerne. Overtrædelse af dette medfører at du ikke længere kan være med i forsøget. Såfremt at du er kvinde i den fødedygtige alder kræves det for deltagelse i forsøget at du anvender sikker prævention. Såfremt dette er tilfældet vil du blive informeret nærmere herom af den forsøgsansvarlige, der følger Sundhedsstyrelsens anbefalinger for sikker prævention.
Forsøget kan afbrydes hvis det ikke længere menes sandsynligt at den indsamlede mængde data giver et validt forsøgsresultat, eller hvis der opstår uventede alvorlige bivirkninger til behandlingen.

Din journal vil blive gennemset med hensyn til opfyldelse af inklusionskriterier samt medicinering, din almene helbredstilstand og din Parkinsons sygdom. Ved din deltagelse i forsøget søges der at den

medicinske behandling er stabil så længe at behandlingen pågår i de 8 uger. Såfremt du skulle opleve symptomer på overdosering vil din medicinske behandling blive ændret.

Ved at du giver samtykke til deltagelse i forsøget giver du tilladelse til adgang til videregivelse og behandling af nødvendige oplysninger om dit heldbred fra patientjournalen, øvrige rent private forhold og andre fortrolige oplysninger som led i sponsors og myndigheders kvalitetskontrol og monitorering,

Dine journaloplysninger og personlige data vil blive behandlet i henhold til persondataloven og loven om tavshedspligt.

Initiativet til forskningsprojektet er taget af en styregruppe bestående af:

Styregruppen: Overlæge, klinisk lektor Lene Wermuth (Ph.d.-vejleder)

Overlæge, dr. med Ole Gredal

Lektor, ph.d. Bente Rona Jensen (Ph.d.-vejleder)

Professor, dr.med. Per Bech (Ph.d.-vejleder)

Læge Bo Mohr Morberg (forsøgsansvarlig)

Der foreligger økonomisk bevilling til såvel gennemførelse af projektet samt Ph.d. forløb fra kommer alene fra DEN A. P. MØLLERSKE STØTTEFOND. Der udbetales driftsomkostninger til den forsøgsansvarlige og drifts omkostninger til den resterende del af projektet. Projektet er forankret i neurologisk afdeling OUH. Der vil kører to studie et i København og et i Odense men begge hører under OUH og der er kun 1 forsøgsansvarlig på begge centre. Den forsøgsansvarlige og styregruppen er ikke tilknyttet DEN A.P MØLLERSKE STØTTEFOND og arbejder således uafhængigt af denne. Fonden har bevilliget kr. 4.962.000 til gennemførelse af forsøget. Du kan få adgang til budget såfremt dette ønskes.

Den forsøgsansvarlige og styregruppen modtager ingen vederlag for gennemførelse af forsøget. Hverken den forsøgsansvarlige eller styregruppen har økonomisk interesse i det firma der leverer apparaterne. Endvidere har den forsøgsansvarlige og styregruppen ingen tilknytning til støttegiveren DEN A.P. MØLLERSKE STØTTEFOND.

Forsøgsansvarlig Bo Mohr Morberg vil stå til rådighed ved yderligere spørgsmål og generel information om forsøget.

Kontakt oplysninger:
Bo Mohr Morberg
Adresse: Kløvervænget 24 b st. 2 5000 Odense C
Mail: bomohrmorberg@gmail.com
Mobil: 28746323

Du opfordres til at læse det vedhæftede tillæg, "Forsøgspersonens rettigheder i et sundhedsvidenskabeligt forskningsprojekt".

Oplysninger om tid og sted for mundtligt informationmøde XXXXX

Du opfordres til at tage en pårørende med til informationsmødet og skrive eventuelle spørgsmål ned som du måtte have og tage dem med til informationsmødet, eller kontakte den forsøgsansvarlige via mail eller telefon inden mødet.

Med venlig hilsen

Læge og forsøgsansvarlig

Bo Mohr Morberg

Skriftlig deltagerinformation til referencegruppe:

Vedr. forskningsprojektet *Behandling af Parkinsons sygdom med transkraniale pulserende elektromagnetiske felter*

# Indsamling af referenceværdier for motoriske funktionsmål.

Det følgende er en forespørgsel om deltagelse i en undersøgelse, der har til hensigt at bestemme referenceværdier for forskellige motoriske funktionsmål hos raske personer i alderen 20-85 år. Undersøgelsen skal konkret bruges som reference til eeffektvurdering af projektet *Behandling med Parkinsons sygdom med transkraniale elektromagnetiske felter* men kan i sin helhed eller i brudstykker også blive anvendt som reference i andre sammenhænge i fremtidige studier.

Motoriske funktionsmål anvendes til bedømmelse af motorisk funktionsniveau hos forskellige patientgrupper og til vurdering af effekten af forskellige interventioner som fx trænings-eller rehabiliteringsforløb, medicinsk og ikke-medicinsk behandling.

For at kunne vurdere en patientgruppe eller en effekt af en intervention, er det vigtigt at have en referenceværdi baseret på data fra alders- og kønsmatchede raske personer.

Vi indsamler derfor data for forskellige funktionsmål på en større referencegruppe og søger derfor raske mænd og kvinder i alderen 20-85 år.

Vi undersøger følgende funktionsmål:

- Sit-to-stand
  Du skal rejse dig op og sætte dig ned på en specialbygget stol 6 gange i træk så hurtigt som muligt.

- Dynamisk postural balance
  Du skal bevæge dig fra side til side, så hurtigt du kan med tilstrækkelig kraft.

- 6 minutters gangtest
  Du skal gå så langt, du kan, på 6 minutter.

- Finmotoriske computer-opgaver
  Du skal løse forskellige opgaver på en computer. Du skal reagere hurtigt, flytte musen hurtigt og præcist både under og uden en samtidig kognitiv opgave, samt klikke lige så hurtigt du kan på musen. Det er ikke en forudsætning, at du er vant til at anvende en computer.

- Fysiologisk tremor (rystelser)
  Du skal lave forskellige opgaver med sensorer klæbet på håndryggen.

- Spørgeskemaer
  Vi vil bede dig udfylde spørgeskemaer vedr. dit almindelige velbefindende samt eventuelle smerter og ubehag i bevægeapparatet.

Den samlede undersøgelse tager ca. 2 timer. Undersøgelsen foregår i Handicaporganisationernes Hus, Blekinge Boulevard 2, 2630 Taastrup. Der er gode parkeringsforhold, og Høje Taastrup st. ligger ca. 300 m derfra (S-tog linje B). Der ydes ingen godtgørelse for deltagelse eller transport i forbindelse med deltagelse.

Det er en forudsætning for deltagelse, at du

- Er 20-85 år
- Ikke lider af sygdomme, der omfatter hjerne, nerver eller muskler (fx parkinson, alzheimer, muskelsvind, neuropati, mv.)
- Ikke for nyligt har gennemgået en operation, der påvirker dit aktuelle fysiske funktionsniveau
- Føler dig sund og rask

Undersøgelsen er finansieret af Den A. P. Møllerske Støttefond samt Grosserer L. F. Foghts Fond.

Interesserede opfordres til at gennemlæse vedhæftede tillæg om forsøgspersoners rettigheder i et sundhedsvidenskabeligt forskningsprojekt.

Er du interesseret i at deltage, er du meget velkommen til at kontakte undertegnede.

Med venlig hilsen

Anne Sofie Bøgh Malling

Videnskabelig assistent

Odense Universitetshospital

Tlf: 28 90 37 33

E-mail: asmallingotmail.com

**DET VIDENSKABSETISKE KOMITESYSTEM**

## Forsøgspersoners rettigheder i et sundhedsvidenskabeligt forskningsprojekt.

Som deltager i et sundhedsvidenskabeligt forskningsprojekt skal du vide at:

- Din deltagelse i forskningsprojektet er helt frivillig og kun kan ske efter, at du har fået både skriftlig og mundtlig information om forskningsprojektet og underskrevet samtykkeerklæringen
- Du til enhver tid mundtligt, skriftligt eller ved anden klar tilkendegivelse kan trække dit samtykke til deltagelse tilbage og udtræde af forskningsprojektet. Såfremt du trækker dit samtykke tilbage påvirker dette ikke din ret til nuværende eller fremtidig behandling eller andre rettigheder, som du måtte have
- Du har ret til at tage et familiemedlem, en ven eller en bekendt med til informationssamtalen
- Du har ret til betænkningstid, før du underskriver samtykkeerklæringen
- Oplysninger om dine helbredsforhold, øvrige rent private forhold og andre fortrolige oplysninger om dig, som fremkommer i forbindelse med forskningsprojektet, er omfattet af tavshedspligt
- Opbevaring af oplysninger om dig, herunder oplysninger i dine blodprøver og væv, sker efter reglerne i lov om behandling af personoplysninger og sundhedsloven
- Der er mulighed for at få aktindsigt i forsøgsprotokoller efter offentlighedslovens bestemmelser. Det vil sige, at du kan få adgang til at se alle papirer vedrørende din deltagelse i forsøget, bortset fra de dele, som indeholder forretningshemmeligheder eller fortrolige oplysninger om andre
- Der er mulighed for at klage og få erstatning efter reglerne i lov om klage- og erstatningsadgang inden for sundhedsvæsenet

*Dette tillæg er udarbejdet af det videnskabsetiske komitésystem og kan vedhæftes den skriftlige information om det sundhedsvidenskabelige forskningsprojekt. Spørgsmål til et projekt skal rettes til den regionale komité, som har godkendt projektet.*

April 2012

# English version of the revised edition of the Danish study protocol (S-20130114)

Version of Dec 4$^{th}$ 2015

Approved by The Regional Scientific Ethical Committees for Southern Denmark Dec 8$^{th}$ 2015

Protocol:

**Aim of the trial:**

The aim of trial is to investigate whether an 8-week treatment with transcranial pulsed electromagnetic fields (T-PEMF) can reduce the severity of motor and non-motor symptoms in patients with idiopathic Parkinson's Disease (IPD).

Furthermore, the aim is to clarify if IPD experience a clinically relevant and statistical significant effect of an active T-PEMF treatment compared to an inactive placebo treatment when treated daily during 8 weeks.

Three types of effect measures are employed in the trial: Clinical, neurophysiological and motor measures. These are described later in the protocol.

**Hypothesis**

We expect that the active T-PEMF treatment is significantly superior to an inactive T-PEMF treatment in regard to decrease the severity of the motor and non-motor symptoms experienced by IPD. We expect that the regression of the symptoms will give IPD a considerably improved quality of life. We expect to collect a valid statistical material that supports our hypothesis.

**Background and literature**

**Parkinson's Disease**

Parkinson's disease (PD) is a progressive neurodegenerating disease, where a substantial loss of dopaminergic neurons in specific brain regions (substantia nigra in particular) occur. The direct cause of IPD is unknown but it is assumed that it is caused by a combination of genetic disposition and external factors such as life circumstances, home conditions, and impact from pesticides, heavy metals, and other possible toxins. Certain families have a family-related overexpression of the disease. In these cases, an early disease debut before the age of 40 is often seen. Currently, more than 10 different genetic changes associated with the disease have been identified. The life long prevalence of PD is about 1 ‰ and it increases with age (more than 3 % at the age +85) [1].

The mean age at diagnose is 60 years. On average, the patients live with the disease for more than 15 years with only a small decrement in expected life duration relative to the general population. Patients with PD often die from secondary complications as pneumonia [2]. As the patients often have to live many years with the disease, an influence of the treatment and hereby the course of disease will be essential for their quality of life.

The decremented dopamine production in the brain is often advanced already when the cardinal motor symptoms in terms of bradykinesia, muscular rigidity, and rest tremor become present. The treatment of PD is symptomatic with the use of dopaminergic medication.

The course of disease is progressive and a decreasing effect of the medical treatment usually occurs after years of therapy. The patients experience the decreasing effect of the medication as fluctuations of the symptoms. Some patients experience a fluctuations between dyskinesia as a sign

of excessive dopamine and Parkinson symptoms as a sign of lag of dopamine. The medical treatment has large consequences for the motor function of the patients and a deterioration of the disease becomes evident through reduced gait function, fall tendency, and reduced fine motor control – all aspects affecting activity-of-daily-living functions.

De relatively few patients with PD with more complicated motor symptoms can be treated with deep brain stimulation (e.g. tremor-dominant PD or patients with severe fluctuations in the treatment effect). Another treatment modality for these patients is the use of a stomach tube. By this treatment modality, the dopaminergic medicine is given directly into the intestine through a pump, which reduces the motor fluctuations.

In addition to the motor symptoms, patients with PD also experience non-motor symptoms. These symptoms can vary a lot from patient to patient but can be just as invalidating as the motor symptoms. The non-motor symptoms includes affection of the autonomic nervous system with the development of e.g. orthostatic hypotension, constipation, micturition disturbances, impotence, and a more general affection of the brain function in general leading to fatigue, sleep disturbances, depression, hallucinations and dementia. The non-motor symptoms do not respond very well on the dopaminergic medication, and several other pharmacologic agents can therefore be assigned; e.g. anti-depressive or anti-dementia medication. This polypharmacy increases the risk of interactions and adverse effects. Even with an adequate medical treatment, the non-motor symptoms can be treatment refractory.

Patients with PD experience reduced health related quality of life. The quality of life is reduced by the motor and non-motor symptoms. Thus, PD can have a large influence on the life circumstances for both the patient and his/her relatives.

**The use of pulsed electromagnetic field (PEMF) in medical science**

In the literature search, we have placed our emphasis on investigating the physiologic effect of T-PEMF and to investigate in what pathological conditions PEMF is used in the clinic including osteoarthrosis and depression. In addition, the search included studies of transcranial electromagnetic stimulation, that basically uses the same treatment principles as T-PEMF.

Magnetic stimulation is used both in psychiatry and neurology for diagnostic and treatment purposes. In neurology, transcranial electromagnetic stimulation (TMS) has been used for the investigation of nerve conduction velocity. Electromagnetic technology is also the basis of MR scans for visualization of organs. In TMS, fast changes in the magnetic field of coil placed above the brain area of interest induces electrical currents in the underlying tissue. In psychiatry, TMS is gaining ground in the clinic in treatment of depressive disorders [3-4], and in neurology TMS has a documented effect on spasticity [5-7]. As the TMS equipment is not portable, the treatment can only be given at clinics and because of the short duration of effect on e.g. spasticity, the treatment has to be repeated several times per week to efficient.

Transcranial pulsed electromagnetic fields (T-PEMF) is in many ways comparable to TMS. However, whereas TMS induces pulsed electric fields in the tissue at an intensity often large enough to induce action potentials in the neurons, T-PEMF uses electromagnetic fields of much lower intensity that does not noticeable change the excitability of the neurons. The T-PEMF device of our study uses pulses of 50 Hz for a

longer time period, usually 30 min, whereas TMS uses few pulses at a low frequency. The T-PEMF treatment is based on an activation of the cortex through small pulsed electric fields of a magnitude and frequency shown to activate multiple cell types and enzymatic processes including neural tissue.

Electromagnetic fields (EMF) have a proven effect on endogenous neural stem cells in the brain, where is seems as EMF can potentiate the proliferation and migration of the neural stem cells and change myelin repair at demyelinisation [8]. In addition, PEMF have been studied for their effect on bone healing [9], clinically for Parkinson’s disease [10] and multiple sclerosis [11]. Investigations of rTMS on animal models of PD have shown the ability of rTMS to change neurotransmitter release, synaptic effects, and induce neurogenesis [12].

The use of 5 Hz rTMS in persons with PD applied over the supplementaty motor cortex showed improved handwriting. This effect was suggested to be explained by changes of the cortical exitability in the neural network [13]. A Danish study of the effect of T-PMF on patients with treatment refractory depression showed an significantly larger effect than sham treatment [14]. A re-analysis of this study showed, that the effect was not only present on the depression symptoms but also the symptoms seen in medical therapy resistant patients such as fatigue, loss of initiative, concentration disturbances and sleep disturbances [15,16]. These non-motor symptoms are also dominant in patients with PD and has a great influence on quality of life [17].

There is evidence of the biological effect of PEMF on different cellular levels. The biological mechanism of action of PEMF is particularly investigated on endothelia cells in the blood-brain barrier [18]. In addition, PEMF has been shown to increase angiogenesis [19], neurite outgrowth [20], peripheral nerve regeneration [21-23], proliferation of chondrocytes [24], and induce vasodilation [25].

Currently, professor per Bech conducts further studies of the dose-response effect of T-PEMF in patients with depression. One group receives one active and one sham session each day, the other group receives two active sessions daily.

### T-PEMF for patients with Parkinson’s disease

Results of a pilot study shows a potential positive effect of T-PEMF in PD [26]. In 2006 consultant doctor Ole Gredal and consultant doctor Lene Wermuth conducted an open study of 8 patients with PD. The aim was to investigate if patients treated with T-PEMF could gain a better motor function (measured by the Unified Parkinson’s Disease Rating Scale), a better quality of life (measured by The Parkinson’s Disease Questionnaire 8) and a reduction in depression symptoms (Major Depression Index). The results showed an improved quality of life (p=0.009) and a reduction in the depression symptoms (p=0.011). In addition, a small improvement in the motor function was detected. The results from this pilot study founded the idea of performing a larger randomized, double blinded, placebo-controlled clinical trial.

The study is considered clinically relevant, as the treatment with T-PEMF for patients with PD lags needs further investigation to determine its effect. If the treatment has a significant effect, it may have an influence on the future treatment strategy of PD.

### Methods of the trial

The investigation of T-PEMF is conducted as double-blinded placebo controlled trial, where half of the Parkinson-patients to an active T-PEMF treatment through a randomization process where randomization.com

is used. The randomization is performed by a person otherwise uninvolved in the trial. The randomization is stratified by age, sex, disease stage (Hoehn Yahr Scale) to minimize bias.

In addition, a group of healthy control subjects of up to 90 subjects will be included to be able to estimate the relative size of an eventual effect of active and sham treatment. The reference group will not receive any treatment.

The active and sham T-EPMF devices have identical appearances and during the treatment a control light flashes. During the sham treatment, no electromagnetic fields are generated. The participants will not be able to feel or heat if they are receiving active or sham treatment. All usage of a given device will be saved on a chip card. Thus, treatment compliance can be controlled.

Investigators and participants are unfamiliar of the treatment allocation. The patients are asked not to mention their experiences of the treatment for the investigators. Violation of this will cause exclusion. Health-care professionals conduct all procedures, including information and instruction of use of the devices.

Statistical calculations based on quality of life results (PDQ-8) from the non-blinded pilot study showed that 120 persons with PD have to be included to expect to show a significant effect on quality of life. The 120 persons with PD will be distributed equally in an active T-PEMF group and a sham T-PEMF group. To obtain an effect on the enzymatic processes in the brian and a clinical effect of the treatment, a treatment duration of at least 4-5 weeks with one daily session is expected to be necessary. Thus, 8 weeks were chosen.

Members will be included and allocated to a treatment in groups of 6 temporally separated by a proximal 6 weeks. This will also enable us to take eventual fluctuations of motor symptoms into account during the test day. At present, we expect to have access to 14 treatment devices, thus having 2 in reserve.

The patients will be sought to be optimally medicated for PD before they are included in the study. The medication will be kept constant during the study. However, if signs of over dosage are seen during the intervention (as result of the T-PEMF or sham treatment), the dosage will be reduced.

The treatment effect will be evaluated by motor and non-motor measures at baseline and endpoint. Follow up evaluation will be performed. In the study three different types of measures will be conducted: Clinical, neuropsychological, and motor measures.

The clinical measures are The Unified Parknison’s Disese Rating Scale (UPDRS) for assessment of symptom severity, and The Parkinson’s Disease Questionnaire 39 (PDQ-39) to assess health related quality of life.

Neurophysiological measures: Depression tendencies will be avaluated by the Major Depression Questionnaire (ICD-10), HAM-D6, The Apathy Scale and WHO-5. Cognition is evaluated through MoCA and MMSE. For the assessment of non-motor symptom severity in general the Non Motor Symptom Scale will be used. For the assessment of cognitive and neurological processes, a Stroop-like test will be conducted. This test relies on the assumption, that people read faster than they can identify color names. The cognitive functions, that are measured in the test, gives information about the capacity of the test person to handle cognitive stress and complex cognitive stimuli.

Motor measures: Resting tremor, gait capacity (e.g. 6 minutes walk test), sit-to-stand (a modified up-and go test), dynamic and static balance.

The reference group is tested once and only in the motor measures and the Stroop test.

The T-PEMF device can store subjective information from the participants in relation to the daily sessions and the usage of the device to determine treatment compliance. In addition, the daily amount of steps will be assessed by a pedometer.

All data will be registered in patient files with the registration of the course, eventual adverse effects, and complications. Data entry in the database of The Danish Society of Movement Disorders or an other database will be performed. In relation to the project, a 5 ml venous blood sample will be taken pre and post intervention. This will be analyzed for BDNF as a marker of neural growth and VEGF as a marker of angiogenesis. The samples will be analyzed on an ongoing basis and no biobank will be established. The blood samples will be taken by the primary investigator.

The motor measurements will be performed on Department of Nutrition, Sport and Exercise, University of Copenhagen.

The clinical and neuropsychological measures will be assessed in OUH and a test center in Taastrup.

**Statistical considerations**

The results from the active and sham T-PEMF group will be compared, and where relevant also compared to the reference group. The statistical department at Department of Regional Health Research will assist with supervision.
Statistical calculations based on quality of life results (PDQ-8) from the non-blinded pilot study showed that 120 persons with PD have to be included to expect to show a significant effect on quality of life. The 120 persons with PD will be distributed equally in an active T-PEMF group and a sham T-PEMF group. To obtain an effect on the enzymatic processes in the brian and a clinical effect of the treatment, a treatment duration of at least 4-5 weeks with one daily session is expected to be necessary. Thus, 8 weeks were chosen. We estimate 60 participants at each center. In relation to the preliminary results of the effect of T-PEMF on depression an active T-PEMF group of 40-50 participant could be sufficient. A power calculation performed on the primary effect measure UPDRS with a 90 % power and a 5 % level of significance showed, that 84 participant with 42 in each group are sufficient. Thus, 60 persons in each group is more than sufficient to also take into account a drop out expected to be about 10 %.

It is not considers necessary to perform interim analysis as previous studies of 8-week T-PEMF in depression did not observe any harmful adverse effects. As the treatment duration is no longer than 8 weeks it is considered ethically responsible to omit an interim analysis.

The design of the study is a randomized clinical trial with a continuous primary effect measure. Data will be anonymized and analyzed by a statistician whom will be blinded to the treatment allocation. Descriptive statistics for the two groups will be generated. At intention to treat analysis LOCT and mixed models will be used. This model assures that missing data is not creating bias if missing data is randomly distributed.

As the T-PEMF treatment (with Re5 devices) is a new approach for treatment of PD, it is valuable to evaluate the effect of the specific treatment modality applied, that is a treatment compliance ≥ 80%.
To reduce the occurrence of missing data, the primary investigator or a qualified deputy ensure that all questionnaires are completed at the given time points. If participants do not show for the arranged tests, they will be contacted telephonically by the primary investigator or a qualified deputy.
Data will be analyzed with SPSS or SAS.

**Inclusion and exclusion criteria for the patients with PD**

*Inclusion criteria for the project are:*

- Patients with idiopathic Parkinson’s disease H & Y 1-4 defined after standard criteria.
- MMSE > 22 (screening tool for incipient dementia).

- Age > 18 years.
- Patient is capable of understanding, accepting and performing the planned procedures.

*Exclusion criteria for the project are:*

- Patient has on time of inclusion previously received T-PEMF treatment.
- Changes in the pharmacological anti-Parkinson treatment within the last 6 weeks.
- Pronounced dementia or other brain damage that can influence the capability of giving informed contest or complicate the assessment of the patient’s condition.
- Psychotic disorder or other psychopathologic conditions that necessitates other intervention.
- Alcohol or drug abuse.
- Deep Brain Stimulation treatment.
- Pregnancy or breast-feeding.
- Epilepsy
- Active implants as pacemaker, cochlear implants and others.
- Active medical equipment as insulin pumps, baclofen pumps and other.
- Participation in other studies or trials in the intervention period.
- Current or previous brain cancer, leukaemia, birthmark cancer, squamous cell cancer or other cancer types in the head/neck region.
- Autoimmune disease.
- Open wounds in the scalp.

There are no demands on the age of the gender and age of the patients as long as they fulfill the inclusion criteria.

**Inclusion and exclusion criteria for the reference group**

*Inclusion criteria for the reference group are:*

- 20-85 years of age
- Participant is capable of understanding, accepting and performing the planned procedures.
- Self-reported subjective feeling of health

*Exclusion criteria for the reference group are:*

- Known neurologic or neuromuscular diseases
- Recent operation that has reduced the current level of physical function

The primary investigator, his qualified deputy or the steering committee reserve the right to dismiss interested reference participants even though they fulfil the inclusion criteria in order to match the final reference group to the patient groups in relation to age and gender distribution.

**Adverse effects, risks and disadvantages**

By transcranial magnetic stimulation (TMS) given at higher intensities, than what is used in the present studies, no long-term adverse events have been observed.

Treatment with electromagnetic field including T-PEMF is generally speaking without discomfort or adverse effects [19]. This is confirmed by an investigations not showing any adverse events measured by the adverse event scale UKU. In the investigations, the amount of treatments has been manifold and have been given over a longer period of time. Preliminary reports on adverse effects of T-PEMF indicates that the treatment can result in mild, transient nausea and head ace. In addition, cases of discomfort of the neck muscles have been reported because of the weight of the head applicator. Currently, no other adverse effects have been reported.

Because the patients can receive the treatment at home and can perform sedentary daily functions as reading or knitting during the treatment, and because of the treatment session of 30 min one time per day, the treatment is not considered a nuisance for the patient. It is emphasized, that the participation in the study does not influence the rights of the patients to receive treatment for his/her PD.

The patients must meet for testing twice in Department of Nutrition, Exercise and Sport, University of Copenhagen (all patients) and tree times at the test center at either Taastrup or Odense (OUH) depending on their geographical inclusion area. This is not considered a considerable inconvenience for the patients. The transport costs for the patient and one relative will be covered. The venous blood sample taken twice during the study is not considered an inconvenience for the patient as only a total of 10 ml of blood is collected and as the primary investigator has great routine in the procedure.

During the treatment period, all adverse events are registered along with an indication of the severity and if the investigator consider the event related to the T-PEMF treatment. The informations are gathered by the UKU. In case of serious adverse events where the T-PEMF treatment can be considered as related, The Regional Scientific Ethical Committee, The Danish Health Authority and the steering committee will be announced immediately.

In addition, The Regional Scientific Ethical Committee, The Danish Health Authority and the steering committee will be announced of any serious events or near-events that occur during the study. By all events, both expected and un-expected events are considered.

The following definitions are applied for medical equipment:

An event meeting the following three criteria requires notification of The Danish Health Authority:

**Adverse Event (AE):** any undesirable event of a participant of a clinical trial by the use of medical equipment disregarding an eventual relation between the event and the use of the medical equipment.

**Adverse Device Effect (ADE):** an undesirable event related to the use of the medical equipment

**Serious Adverse Event (SAE/near-event):** an event causing

- death
- life threatening injury or disease
- permanent injury of the body or body functions
- hospitalizing or elongation of hospitalizing

- the necessity of medical or surgical treatment to avoid the above
- foetal death, an inherent anomaly or malformation of the foetus or other negative influence on the foetus

A near-evet is an equipment related evet that does not have a serious outcome because actions were taken before the event has progressed.

**Also including**: serious events and near-events caused by inaccurate or faulty results from diagnostic devices, e.g.:

- misdiagnosis
- delayed diagnosis
- delayed treatment
- erroneous treatment
- and where the error is due to technical errors and omissions of the equipment, manual, labelling, use, or maintenance of the equipment.

**Serious Adverse Device effect (SADE):** a serious event that is related to the use of the medical equipment.

The study will be conducted according to ISO 14155 and monitored by the Good Clinical Practice unit at Odense University Hospital.

It is deemed to be without any risk or inconvenience to participate in the reference group.

**Respect for the physical and mental integrity and the sanctity of private life of the participants**

Informations about the participants are protected by The Act on Processing of Personal Data and The Danish Health Act. The project id registered by The Danish Data Protection Agency and the study is conducted in compliance with The Danish Data Protection Act.
The primary investigator wishes to use data from the medical records of the participants in the study. This regards data on pharmacological treatment, disease stage, general statement of health, and specialty data regarding Parkinson's disease. Data is used to include the participants in the study.
Only relevant parts of the above described parts of the medical record will be read. These data are considered necessary in the study. The participants will be thoroughly informed on the extent of the readings of medical records and the foundation of the readings.
No medical records are obtained for the participants in the reference group.

**Economic conditions.**
The primary investigator and the steering committee recieves to remuneration for conducting the study. Neither the primary investigator nor the steering committee have economic interest in the company producing the T-PEMF devices (Re5). In addition, the primary investigator and the steering committee have no associations with the funding source, Den A. P. Møllerske Støttefond.

The initiative of the study was taken by the steering committee  comprising:

Consultant, clinical professor Lene Wermuth (Primary Ph.d.-supervisor)

Consultant, Dr.Med Ole Gredal

Associate professor, Ph.d. Bente Rona Jensen (Ph.d.-supervisor)

Professor, Dr.Med. Per Bech (Ph.d.-supervisor)

Doctor Bo Mohr Morberg (primary investigator)

A grant for conductance of the study and a Ph.d. programme has been given by Den A. P. Møllerske Støttefond by an amount of dkk 4 962 000 (see the budget, appendix 13).

The clinical study is lead by the Neurological Research Unit, OUH. The grant is administered by OUH and is subject to auditing of public accounts.

The grant is payed in one rate and to research accounts are established within OUH. One account covering salary expenses for the primary investigator and one account covering the running costs.

An eventual surplus of the grant will be returned to the foundation.

**Relevant clauses in the contract between sponsor and the place of study conduction**
There are no clauses between den A.P. Møllerske Støttefond and the primary investigator or the steering committee with regard to publication of study results. The primary investigator has full access to data and the full rights for publishing data. The manufacturer of the T-PEMF device has no access to data.

**Remuneration or other granting of the participants.**
The participants in the treatment groups receive transport compensation according to current tariffs of transport compensation by driving in private car or the lowest cost public transportation. This prevails the tests of participants in OUH and Taastrup. In addition, the transport expenses for one relative is covered. The participants in the reference group receives no remuneration or other granting.

**Recruitment of participants**
The project is expected to recruit patients from to geographical areas – the Region of Southern Denmark and The Capita Region of Denmark. Through contact to the respective neurological departments, private practicing neurologists, and The Local Danish Parkinson's Associations, patients with PD will be invited to participate in information meetings with particular reference to later inclusion and participation in the study. Two inclusion centers are planned – one in department of neurology, OUH and one in Handicaporganisationernes Hus, Taastrup.

Participants of the reference group are sought to be recruited through relatives of the participants with PD and through contact to associations and organizations in Taastrup and neighboring areas. Recruitment will primarily be from Zealand to reduce own expenses of the participants. Persons without relatives participating or contact with local associations or organizations can also participate. Potential participants, association and organizations are contacted by e-mail with the written participant information attached. The contact information of research assistant Anne Sofie Bøgh Malling, whom can be contacted upon interest, is found in the written participant information.

**Accessibility of information for participants.**
The participant are supplied with contact information of relevant health care personnel that can assist answering questions, that the participants might have. The participants will have access to contact information on the research assistant and primary investigator. Each participant will in his/her participation period be affiliated with one of the above mentioned contact persons. The participants will, if they have consented to it,

be informed of the results of the study written in easily readable language, when the study is terminated and the scientific evaluation is completed.

**Publication of the results**

The results from the study is expected to contribute with 2-4 scientific articles that can be published in international peer-reviewed journals, together with presentations at national and international meetings and conferences. The results will be sought to be published no matter if they show positive, negative or inconclusive results.

**Scientific ethical statement**

The study is conducted in accordance with Danish law with notification of The Danish Data Protection Agency, The Regional Scientific Ethical Committee, and The Danish Health Authority. Data management is performed properly and according to current rules and The Danish Data Protection Act.

The protocol from 2006 is previously approved by The Regional Scientific Ethical Committee (# 01 278045) and by The Danish Health Authority, but a reapplication is required as is an application for the The Danish Health Authority for the use of the T-PEMF devices.

For a treatment of PD to be clinically effective and secure, the treatment have to be in line with the following criteria: The treatment has an effect on the disease including an increase in the quality of life and has an acceptable adverse effect profile from a patient and medical consultant point of view.

T-PEMF have previously been indicated to have an effect on quality of life and depression tendencies in PD [26]. It is essential to investigate if these results can be validated through larger and randomized trial. In addition, it is essential to gain insight into whether patients having received T-PEMF once can benefit from receiving it again, and if patients with PD can get relief of their motor and non-motor symptoms.

Investigations indicate that the adverse effect profile id positive with relatively mild and transient adverse effects. Until now, T-PEMF has only resulted in few, short lasting adverse effects. However, it is crucial to investigate the current reports on adverse effects is concurrent with the actual prevalence of adverse effects caused by T-PEMF treatment.

As the study relies on the usage of an active and a sham treatment group it is very relevant to investigate if the use of sham enables an ethical dilemma. To investigate if a treatment method is effective it is necessary also to investigate the effect of a placebo (sham) treatment. As no serious adverse effects of T-PEMF has been reported and there is scientific evidence of a physiologic effect of T-PEMF, it is considered ethical responsible to include a sham group in the study. In addition, the participant in the sham still have the equal rights of medical treatment as non-participating subjects. Thus, all participating patients will receive treatment in line with non-participating subjects.

We do not find that participation in the study affects how the participants can perform their daily activities as the T-PEMF treatment gives minimal negative influence on the everyday life.

All participants will be informed that they after inclusion will be allocated to either active or sham treatment. Thus, the participants have the opportunity to reject participation.

If the results of the current study shows a positive effect of the treatment and an acceptable adverse effect profile, the treatment could be a potential new treatment modality for PD.

**Information about insurance- and remuneration arrangements**

The participants are covered by the Danish Patient Insurance Association covering the harms and injuries that participant may experience in relation to health science research projects. The study is conducted under the direct responsibility of OUH.

**Protocol resumé**

**Titel**

T-PEMF (Transcranial Pulsed ElectroMagnetic Fields) in Parkinson's disease.

**Primary investigator**

Bo Mohr Morberg, Department of Neurology, OUH.

**Aim of the study**

The aim of trial is to investigate whether an 8-week treatment with transcranial pulsed electromagnetic fields (T-PEMF) can reduce the severity of motor and non-motor symptoms in patients with idiopathic Parkinson's Disease (IPD).

Furthermore, the aim is to clarify if IPD experience a clinically relevant and statistical significant effect of an active T-PEMF treatment compared to an inactive placebo treatment when treated daily during 8 weeks.

**Method**

The T-PEMF study i conducted as adouble blinded placebo controlled randomized trial. Half of the up to 120 patients are allocated to active T-PEMF treatment, half to sham treatment. Stratification of age, gender, and disease severity (Hoehn and Yahr Scale) is applied. Active and sham devices will appear identical and a control lamp will flash during treatment. In sham treatment, no electromagnetic fields are generated. The participants will not be able to feel if they recieve active or sham treatment. The patients are asked not to mention their experiences of the treatment for the investigators. All procedures including information and instruction of use of the device is conducted of health care professionals.

The up to 120 patients are equally distributed between active and sham treatment. The duration of the treatment is 8 weeks as structural changes in brain tissue takes time. Groups of 6 patients will initiate treatment separated by approximately 6 weeks.

In addition, a healthy reference group not receiving any treatment is included.

In relation to the project, a 5 ml venous blood sample will be taken pre and post intervention. This will be analyzed for BDNF as a marker of neural growth and VEGF as a marker of angiogenesis. The samples will be analyzed on an ongoing basis and no biobank will be established. The blood samples will be taken by the primary investigator at baseline and endpoint.

Patients are sought to be optimally medicated before inclusion. The medication has to be stable throughout the intervention.

**Statistical considerations**

The results from the active and sham T-PEMF group will be compared, and where relevant also compared to the reference group. The statistical department at Department of Regional Health Research will assist with supervision.
Statistical calculations based on quality of life results (PDQ-8) from the non-blinded pilot study showed that 120 persons with PD have to be included to expect to show a significant effect on quality of life. The 120 persons with PD will be distributed equally in an active T-PEMF group and a sham T-PEMF group. To obtain an effect on the enzymatic processes in the brian and a clinical effect of the treatment, a treatment duration of at least 4-5 weeks with one daily session is expected to be necessary. Thus, 8 weeks were chosen. We estimate 60 participants at each center. In relation to the preliminary results of the effect of T-PEMF on depression an active T-PEMF group of 40-50 participant could be sufficient. A power calculation performed on the primary effect measure UPDRS with a 90 % power and a 5 % level of significance showed, that 84 participant with 42 in each group are sufficient. Thus, 60 persons in each group is more than sufficient to also take into account a drop out expected to be about 10 %.

**Participants, inclusion and exclusion criteria**

*Inclusion criteria for the project are:*

- Patients with idiopathic Parkinson's disease H & Y 1-4 defined after standard criteria.
- MMSE > 22 (screening tool for incipient dementia).
- Age > 18 years.
- Patient is capable of understanding, accepting and performing the planned procedures.

*Exclusion criteria for the project are:*

- Patient has on time of inclusion previously received T-PEMF treatment.
- Changes in the pharmacological anti-Parkinson treatment within the last 6 weeks.
- Pronounced dementia or other brain damage that can influence the capability of giving informed contest or complicate the assessment of the patient's condition.
- Psychotic disorder or other psychopathologic conditions that necessitates other intervention.
- Alcohol or drug abuse.
- Deep Brain Stimulation treatment.
- Pregnancy or breast-feeding.
- Epilepsy
- Active implants as pacemaker, cochlear implants and others.
- Active medical equipment as insulin pumps, baclofen pumps and other.

- Participation in other studies or trials in the intervention period.
- Current or previous brain cancer, leukaemia, birthmark cancer, squamous cell cancer or other cancer types in the head/neck region.
- Autoimmune disease.
- Open wounds in the scalp.

There are no demands on the age of the gender and age of the patients as long as they fulfill the inclusion criteria.

**Inclusion and exclusion criteria for the reference group**

*Inclusion criteria for the reference group are:*

- 20-85 years of age
- Participant is capable of understanding, accepting and performing the planned procedures.
- Self-reported subjective feeling of health

*Exclusion criteria for the reference group are:*

- Known neurologic or neuromuscular diseases
- Recent operation that has reduced the current level of physical function

The primary investigator, his qualified deputy or the steering committee reserve the right to dismiss interested reference participants even though they fulfil the inclusion criteria in order to match the final reference group to the patient groups in relation to age and gender distribution.

**Adverse effects, risks and disadvantages**

Treatment with electromagnetic field including T-PEMF is generally speaking without discomfort or adverse effects [19]. This is confirmed by an investigations not showing any adverse events measured by the adverse event scale UKU. In the investigations, the amount of treatments has been manifold and have been given over a longer period of time. Preliminary reports on adverse effects of T-PEMF indicates that the treatment can result in mild, transient nausea and head ace. In addition, cases of discomfort of the neck muscles have been reported because of the weight of the head applicator. Currently, no other adverse effects have been reported.

Because the patients can receive the treatment at home and can perform sedentary daily functions as reading or knitting during the treatment, and because of the treatment session of 30 min one time per day, the treatment is not considered a nuisance for the patient. It is emphasized, that the participation in the study does not influence the rights of the patients to receive treatment for his/her PD. The venous blood sample taken twice during the study is not considered an inconvenience for the patient as only a total of 10 ml of blood is collected and as the primary investigator has great routine in the procedure.

No long-term adverse effects have been reported for electromagnetic field of much larger field intensity than used in PEMF.

During the treatment period, all adverse events are registered along with an indication of the severity and if the investigator consider the event related to the T-PEMF treatment. The informations are gathered by the UKU. In case of serious adverse events where the T-PEMF treatment can be considered as related, The Regional Scientific Ethical Committee, The Danish Health Authority and the steering committee will be announced immediately.

In addition, The Regional Scientific Ethical Committee, The Danish Health Authority and the steering committee will be announced of any serious events or near-events that occur during the study. By all events, both expected and un-expected events are considered.

There are no risks by participating in the reference group.

**Economic conditions.**
The primary investigator and the steering committee recieves to remuneration for conducting the study. Neither the primary investigator nor the steering committee have economic interest in the company producing the T-PEMF devices (Re5). In addition, the primary investigator and the steering committee have no associations with the funding source, Den A. P. Møllerske Støttefond.

The initiative of the study was taken by the steering committee comprising:

Consultant, clinical professor Lene Wermuth (Primary Ph.d.-supervisor)

Consultant, Dr.Med Ole Gredal

Associate professor, Ph.d. Bente Rona Jensen (Ph.d.-supervisor)

Professor, Dr.Med. Per Bech (Ph.d.-supervisor)

Doctor Bo Mohr Morberg (primary investigator)

A grant for conductance of the study and a Ph.d. programme has been given by Den A. P. Møllerske Støttefond by an amount of dkk 4 962 000 (see the budget, appendix 13).

The clinical study is lead by the Neurological Research Unit, OUH. The grant is administered by OUH and is subject to auditing of public accounts.

The grant is payed in one rate and to research accounts are established within OUH. One account covering salary expenses for the primary investigator and one account covering the running costs.

An eventual surplus of the grant will be returned to the foundation.

**Publication of the results**
The results from the study is expected to contribute with 2-4 scientific articles that can be published in international peer-reviewed journals, together with presentations at national and international meetings and conferences. The results will be sought to be published no matter if they show positive, negative or inconclusive results.

**Scientific ethical statement**
The study is conducted in accordance with Danish law with notification of The Danish Data Protection Agency, The Regional Scientific Ethical Committee, and The Danish Health Authority. Data management is performed properly and according to current rules and The Danish Data Protection Act.

The protocol from 2006 is previously approved by The Regional Scientific Ethical Committee (# 01 278045) and by The Danish Health Authority, but a reapplication is required as is an application for the The Danish Health Authority for the use of the T-PEMF devices.

For a treatment of PD to be clinically effective and secure, the treatment have to be in line with the following criteria: The treatment has an effect on the disease including an increase in the quality of life and has an acceptable adverse effect profile from a patient and medical consultant point of view.

T-PEMF have previously been indicated to have an effect on quality of life and depression tendencies in PD [26]. It is essential to investigate if these results can be validated through larger and randomized trial. In addition, it is essential to gain insight into whether patients having received T-PEMF once can benefit from receiving it again, and if patients with PD can get relief of their motor and non-motor symptoms.

Investigations indicate that the adverse effect profile id positive with relatively mild and transient adverse effects. Until now, T-PEMF has only resulted in few, short lasting adverse effects. However, it is crucial to investigate the current reports on adverse effects is concurrent with the actual prevalence of adverse effects caused by T-PEMF treatment.

As the study relies on the usage of an active and a sham treatment group it is very relevant to investigate if the use of sham enables an ethical dilemma. To investigate if a treatment method is effective it is necessary also to investigate the effect of a placebo (sham) treatment. As no serious adverse effects of T-PEMF has been reported and there is scientific evidence of a physiologic effect of T-PEMF, it is considered ethical responsible to include a sham group in the study. In addition, the participant in the sham still have the equal rights of medical treatment as non-participating subjects. Thus, all participating patients will receive treatment in line with non-participating subjects.

We do not find that participation in the study affects how the participants can perform their daily activities as the T-PEMF treatment gives minimal negative influence on the everyday life.

All participants will be informed that they after inclusion will be allocated to either active or sham treatment. Thus, the participants have the opportunity to reject participation.

If the results of the current study shows a positive effect of the treatment and an acceptable adverse effect profile, the treatment could be a potential new treatment modality for PD.

**Recruitment of participants**

The project is expected to recruit patients from to geographical areas – the Region of Southern Denmark and The Capita Region of Denmark. Through contact to the respective neurological departments, private practicing neurologists, and The Local Danish Parkinson's Associations, patients with PD will be invited to participate in information meetings with particular reference to later inclusion and participation in the study. Two inclusion centers are planned – one in department of neurology, OUH and one in Handicaporganisationernes Hus, Taastrup.

Participants of the reference group are sought to be recruited through relatives of the participants with PD and through contact to associations and organizations in Taastrup and neighboring areas. Recruitment will primarily be from Zealand to reduce own expenses of the participants. Persons without relatives participating or contact with local associations or organizations can also participate. Potential participants, association and organizations are contacted by e-mail with the written participant information attached. The contact information of research assistant Anne Sofie Bøgh Malling, whom can be contacted upon interest, is found in the written participant information.

**Oral participant information**

The oral participant infomation is given by the primary investigator. The contact to potential participants of the patient group is conducted by letter containing the written participant information with an invitation to an oral information meeting. Participants have the opportunity to bring a relative to the oral information meeting as assessor. The participants, accepting participation in the study, will receive individual oral information before signing the informed consent.
Participants the reference group are informed individually over the phone by the primary investigator or his deputy. The written participant information for the reference group is send if not obtained through recruitment. The oral information will be repeated prior to signing informed consent on the test day.

**The following will be enclosed in the invitation for the patient group (beside the written participant information)**

- Date and time of the information meeting
- It is emphasized that the invitation concerns an enquiry about participation in a health science research project.
- It is emphasized that it is possible to bring an assessor to the meeting.

Attention is drawn to the fact that there is a 2-day reflection period after the meeting to decide if participation in the study is desired. In addition, attention is drawn to the fact that at acceptance of participation an additional individual meeting with the primary investigator will be arranged and here the final accept can be given from the participant through written informed consent.

The information meetings will be held at department of Neurology, OUH, and in Handikaporganisationernes Hus, Taastrup. The meeting will be held late afternoon where fewest disturbances are espected. The meeting time is arranged so that the participants can be expected to be cognitively game.

After the oral information meeting the patients can go home and discuss their potential participation with their relatives. The patients will be given an informed consent form from The Scientific Ethical Committee at the meeting to bring home. Patients are given contact details of the primary investigator in case of matters of dispute.

The patients will recieve the written participant information at least 14 days before the oral meeting, giving them time to read through the material beforehand and prepare eventual questions. The patients have to sign up for the meeting at least 7 days in advance. After the meeting, there will be opportunities to talk to the primary investigator in more informal settings if desired.

The oral information is given through easily understood and easily read Power Point slides that are also given to the participants at the beginning of the meeting. Thus, all medical terms will presented in regular Danish language.

Information about possible adverse effects, the possibility of unforeseen events, and possible disadvantages by participating in the study are given and the requirement of high treatment compliance was set.

The participants will have their medical record examined in relation to current treatment and other diseases. The participants are informed, that they participate in a research project that may determine if a new treatment

modality for PD can be available in the future.

The consent form from The Scientific Ethical Committee will be used for written informed consent.

In addition, a written mandate from the participant to The Danish Health Authority to review the medical journal in relation to audit of trial of medical devices is obtained.

If information about the effect, adverse effects or complications of the treatment is found during the study, the participants will be informed. In addition, the participants will be informed if the study design is changed if it is considered relevant for the patient safety.

The participant will, if he/she wishes, receive information about his/her health status if essential information about the health status is obtained during the study.

The participant will, if he/she wishes, receive information about the results of the study and the potential consequences for the participant after the termination of the study.

If the study is terminated prematurely the participants will be informed about the reason.

Essential health status data will be announced to the participant.

Literature

[1]. Parkinsons sygdom. Klinisk vejledning 2 udgave. Danmodis; 2011

[2]. Lees AJ, Hardy J, Revisz T. Parkinson's disease. Lancet 2009;373:2055-6

[3]. Bretlau L, Lunde MA, Lindberg L, Unden M, Bech P. Repetitive Transcranial Magnetic Stimulation (rTMS) in combination with escitalopram in patients with treatment-resistant major depression. A double-blind, randomised, sham-controlled trial. Pharmacopsychiatry. 2008;41:41-47

[4]. Martiny K et al. Pulsed electromagnetic fields (PEMF) in treatment of resistant depression. A pilot study. International Society of Affective Disorders (Abstract). Second Biennial International Conference. Cancun, Mexico March 5th - 10th, 2004

[5]. Centonze D, Koch G, Versace V, Mori F, Rossi S, Brusa L, et al. Repetitive transcranial magnetic stimulation of the motor cortex ameliorates spasticity in multiple sclerosis. Neurology 68, 1045-1050, 2007

[6]. Krause P, Edrich T, Straube A. Lumbar repetitive magnetic stimulation reduces spastic tone increase of the lower limbs. Spinal cord 42, 67-72. 2004

[7]. Nielsen J.F, Sinkjaer T, Jakobsen J. Treatment of spasticity with repetitive magnetic stimulation; a double-blind placebo-controlled study. Mult Scler 2, 227-32,1996

[8]. Sherafat MA, Heibatollahi M, Mongabadi S, Moradi F, Javan M, Ahmadiani A. Electromagnetic Field Stimulation Potentiates Endogenous Myelin Repair by Recruiting Subventricular Neural Stem Cells in an Experimental Model of White Matter Demyelination. J Mol Neurosci. 2012 Sep;48(1):144-53

[9] Borsalino G, Bagnacani M, Bettati E et al. Electrical stimulation of human femoral intertrochanteric osteotomies. Double-blind study. Clin Orthop Relat Res 1988; (237):256- 63

[10] Sandyk R. Speech impairment in Parkinson's disease is improved by transcranial application of electromagnetic fields. Int J Neurosci. 1997 Nov;92(1-2):63-72

[11] Sandyk R. Treatment with electromagnetic fields improves dual-task performance (talking while walking) in multiple sclerosis. Int J Neurosci. 1997 Nov;92(1-2):95-102

[12]. Arias-Carrión O. Basic mechanisms of rTMS: Implications in Parkinson's disease. Int Arch Med. 2008 Apr 15;1(1):2

[13]. Randhawa BK, Farley BG, Boyd LA. Repetitive transcranial magnetic stimulation improves handwriting in Parkinson's disease. Parkinsons Dis. 2013;2013:751925

[14]. Martiny K, Lunde M, Bech P. Transcranial low voltage pulsed electromagnetic fields in patients with treatment-resistant depression. Biol Psychiatry. 2010 Jul 15;68(2):163-9. doi: 10.1016/j.biopsych.2010.02.017. Epub 2010 Apr 10

[15]. Bech P, Gefke M, Lunde M, Lauritzen L, Martiny K. The pharmacopsychometric triangle to illustrate the effectiveness of T-PEMF concomitant with antidepressants in treatment resistant patients: A double-blind, randomised, sham-controlled trial revisited with focus on the patient-reported outcomes. Depression Research and Treatment. Volume 2011, Article ID 806298, 6 pages

[16]. Andreasson K, Liest V, Lunde M, Martiny K, Unden M, Dissing S et al. Identifying patients with therapy-resistant depression by using factor analysis. Pharmacopsychiatry 2010;43:252-256

[17]. Santos-García D et al. Impact of non-motor symptoms on health-related and perceived quality of life in Parkinson's disease. J Neurol Sci. 2013 Jul 25. pii: S0022-510X(13)00316-X

[18]. Ulrik L Rahbek, Katerina Tritsaris, Steen Dissing. Interaction of Low-frequency, Pulsed Electromagnetic Fields with Living Tissue: Biochemical Responses and Clinical Results. Vol 2. No 1. 2005. Oral Biosciences & Medicine. 1-12

[19]. Oren M. Tepper, Matthew J. Callaghan, Edward I. Chang, Robert D. Galiano, Kirit A. Bhatt, Samuel Baharestani, et al. Electromagnetic fields increase in vitro and in vivo angiogenesis through endothelial release of FGF-2. *The FASEB Journal* express article10.1096/fj.03-0847fje. Published online June 18, 2004

[20] Macias MY, Battocletti JH, Sutton CH, Pintar FA, Maiman DJ. Directed and enhanced neurite growth with pulsed magnetic field stimulation. Bioelectromagnetics, 21 (2000), pp. 272–286

[21] Longo FM, Yang T, Hamilton S, Hyde JF, Walker J, Jennes L et al. Electromagnetic fields influence NGF activity and levels following sciatic nerve transection. J Neurosci Res 1999, 55:230-237

[22] Macias MY, Battocletti JH, Sutton CH, Pintar FA, Maiman DJ. Directed and enhanced neurite growth with pulsed magnetic field stimulation. Bioelectromagnetics 2000, 21:272-286

[23] Sisken BF, Kanje M, Lundborg G, Herbst E, Kurtz W. Stimulation of rat sciatic nerve regeneration with pulsed electromagnetic fields. Brain Res 1989. 485:309-316

[24] Pezetti F, De MM, Caruso A, Cadossi R, Zucchini P, Carinci F. Effect of pulsed electromagnetic fields om human chodrocytes: an in vitro study. Calcif Tissue Int 1999, 65:396-401

[25] Smith TL, Wong-Gibbons D, Maultsby J. Microcirculatory effects of pulsed electromagnetic fields. J Orthop Res. 2004 Jan;22(1):80-4.

[26]. Dethlefsen G, Gredal O, Wermuth L. Effect of pulsed electro magnetic fields in patients with idiopathic Parkinsons disease: A pilot study. Fremlagt som poster ved ”8th International Conference on Alzheimer's and Parkinson's Diseases AD/PD. March 14-18, 2007, Salzburg, Austria

[27]. Martiny K, Lunde M, Bech Transcranial low voltage pulsed electromagnetic fields in patients with treatment-resistant depression. Psychiatry. 2010 Jul 15;68(2):163-9. doi: 10.1016/j.biopsych.2010.02.017. Epub 2010 Apr 10.
